# Supplementary material for: New Bicyclic Pyridine-Based Hybrids Linked to the 1,2,3-Triazole Unit: Synthesis via Click Reaction and Evaluation of Neurotropic Activity and Molecular Docking
Source: Molecules. 2023 Jan 17;28(3):921. doi: 10.3390/molecules28030921 (PMC9920413; doi:10.3390/molecules28030921)
Supplement: Supplementary file 1 [file molecules-28-00921-s001.zip › molecules-2106894-supplementary.pdf]

Supporting Information File

New bicyclic pyridine based hybrids linked to 1,2,3-triazole unit: synthesis *via* click reaction and evaluation of neurotropic activity and molecular docking

Samvel N. Sirakanyan[1,*], Domenico Spinelli[2,*], Anti Petrou[3], Athina Geronikaki[3], Victor G. Kartsev[4], Elmira K. Hakobyan[1], Hasmik A. Yegoryan[1], Luca Zuppiroli[5], Riccardo Zuppiroli[5], Armen G. Ayvazyan[6], Ruzanna G. Paronikyan[1], Tatevik A. Arakelyan[1], Anush A. Hovakimyan[1]

[1] Scientific Technological Center of Organic and Pharmaceutical Chemistry of National Academy of Science of Republic of Armenia, Institute of Fine Organic Chemistry of A.L.Mnjoyan, Armenia 0014, Yerevan, Ave. Azatutyan 26; shnnr@mail.ru (S.N.S.); hakobyan.elmira@mail.ru (E.K.H.); hasmik.yegoryan@mail.ru (H.A.Y.); aaa.h.87@mail.ru (A.A.H.); paronikyan.ruzanna@mail.ru (R.G.P.); tatev160396@mail.ru (T.A.A.)

[2] Dipartimento di Chimica G. Ciamician, Alma Mater Studiorum-Università di Bologna, Via F. Selmi 2, Bologna 40126, Italy; domenico.spinelli@unibo.it

[3] Aristotle University of Thessaloniki, School of Pharmacy, Thessaloniki54124, Greece; geronik@pharm.auth.gr (A.G.); anthi.petrou.thessaloniki1@gmail.com (A.P)

[4] InterBioScreen, Moscow 119019, Russia; vkartsev@ibscreen.chg.ru

[5] Department of Industrial Chemistry 'Toso Montanari', Alma Mater Studiorum-Università di Bologna, Viale del Risorgimento 4, 40136 Bologna, Italy; luca.zuppiroli@unibo.it (L.Z.); riccardo.zuppiroli@studio.unibo.it (R.Z.)

[6] Scientific Technological Center of Organic and Pharmaceutical Chemistry of National Academy of Science of Republic of Armenia, Molecule Structure Research Centre, Armenia 0014, Yerevan, Ave. Azatutyan 26; armenayv@gmail.com

*Corresponding authors Emails:

Domenico Spinelli – domenico.spinelli@unibo.it

Samvel Sirakanyan – shnnr@mail.ru

The copies of ^{1}H, ^{13}C NMR and MS spectra of all new synthesized compounds

2b

Molecular Structure Research Centre, Yerevan, Armenia, Varian Mercury-300VX H1 300.088 MHz, nt = 16, np = 32000, temp = 30.0 C, lb = -0.2, solvent = DMSO/CCl4 1/3 **Oct 4 2021**

T20-145

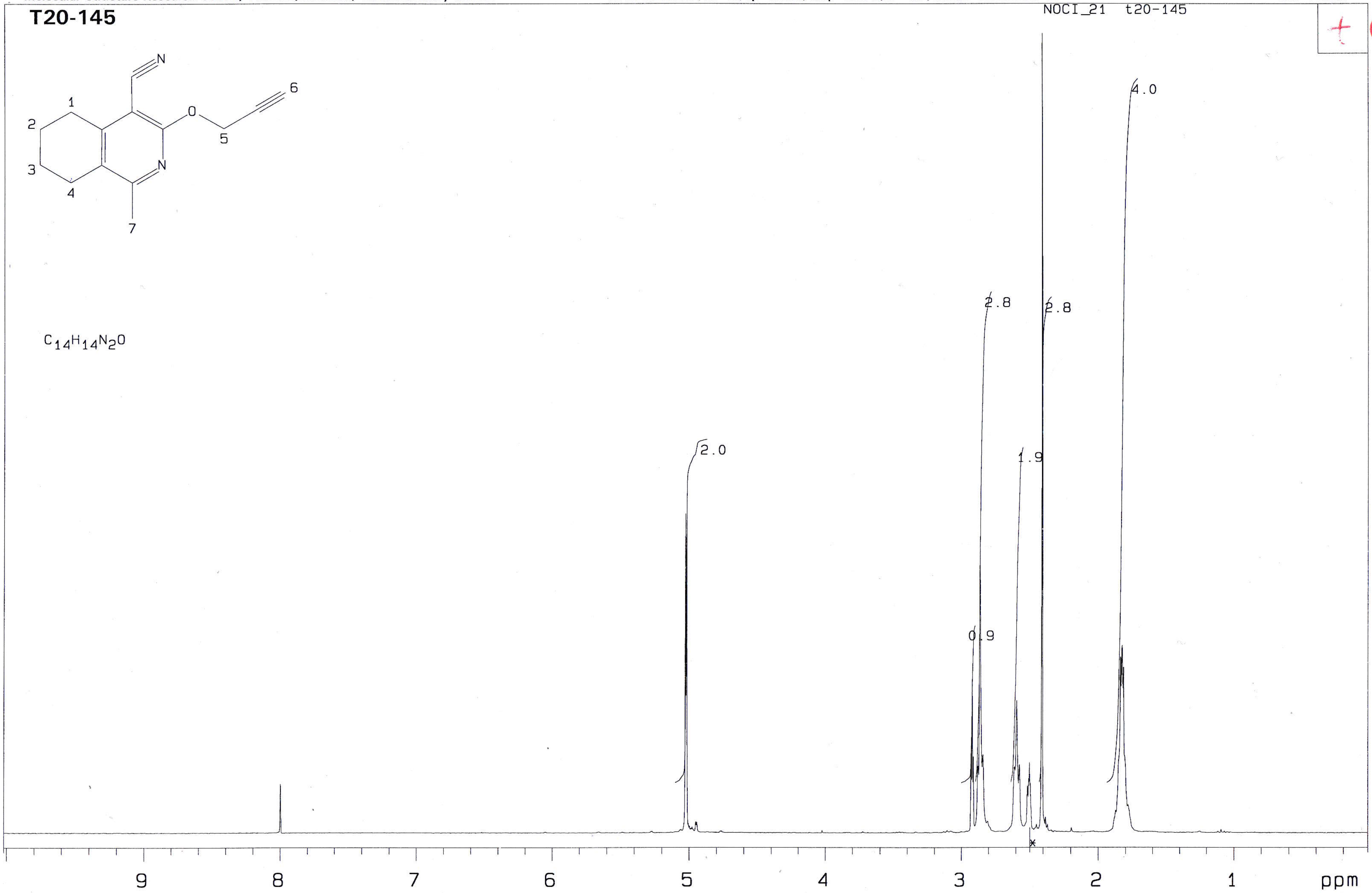

2 6

Molecular Structure Research Centre, Yerevan, Armenia, Varian Mercury-300VX

C13 75.465 MHz, nt = 288, np = 19998, temp = 30.0 C, lb = 1.0, solvent = DMSO/CCl4 1/3

Oct 4 2021

NOCI_21 t20-145

T20-145

$C_{14}H_{14}N_2O$

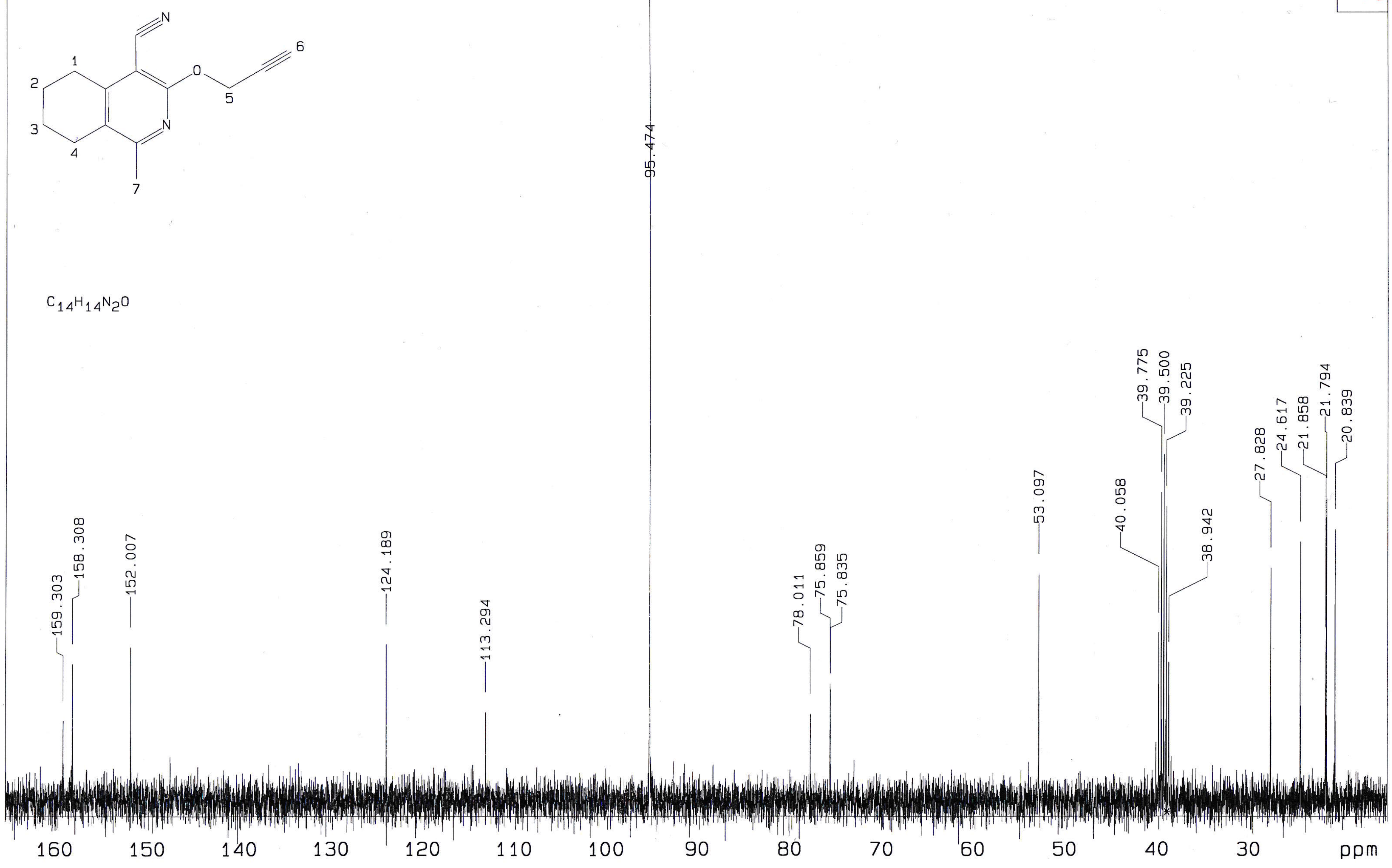

2d

Molecular Structure Research Centre, Yerevan, Armenia, Varian Mercury-300VX

H1 300.088 MHz, nt = 16, np = 32000, temp = 30.0 C, lb = -0.2, solvent = DMSO/CCl4 1/3

Sep 26 2018

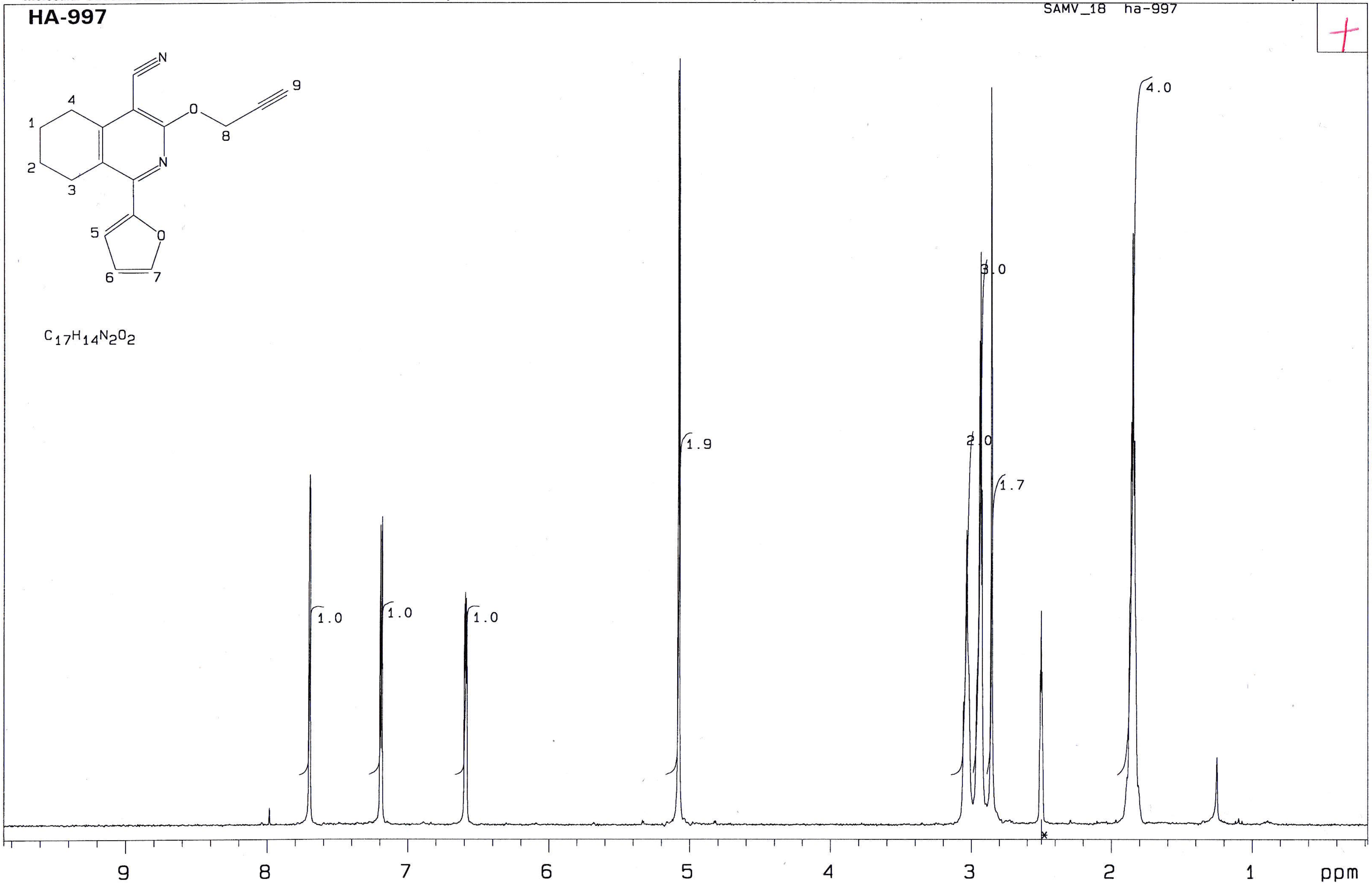

2d

Molecular Structure Research Centre, Yerevan, Armenia, Varian Mercury-300VX — C13 75.465 MHz, nt = 176, np = 19998, temp = 30.0 C, lb = 1.0, solvent = DMSO/CCl4 1/3 — Sep 26 2018

SAMV_18 ha-997

HA-997

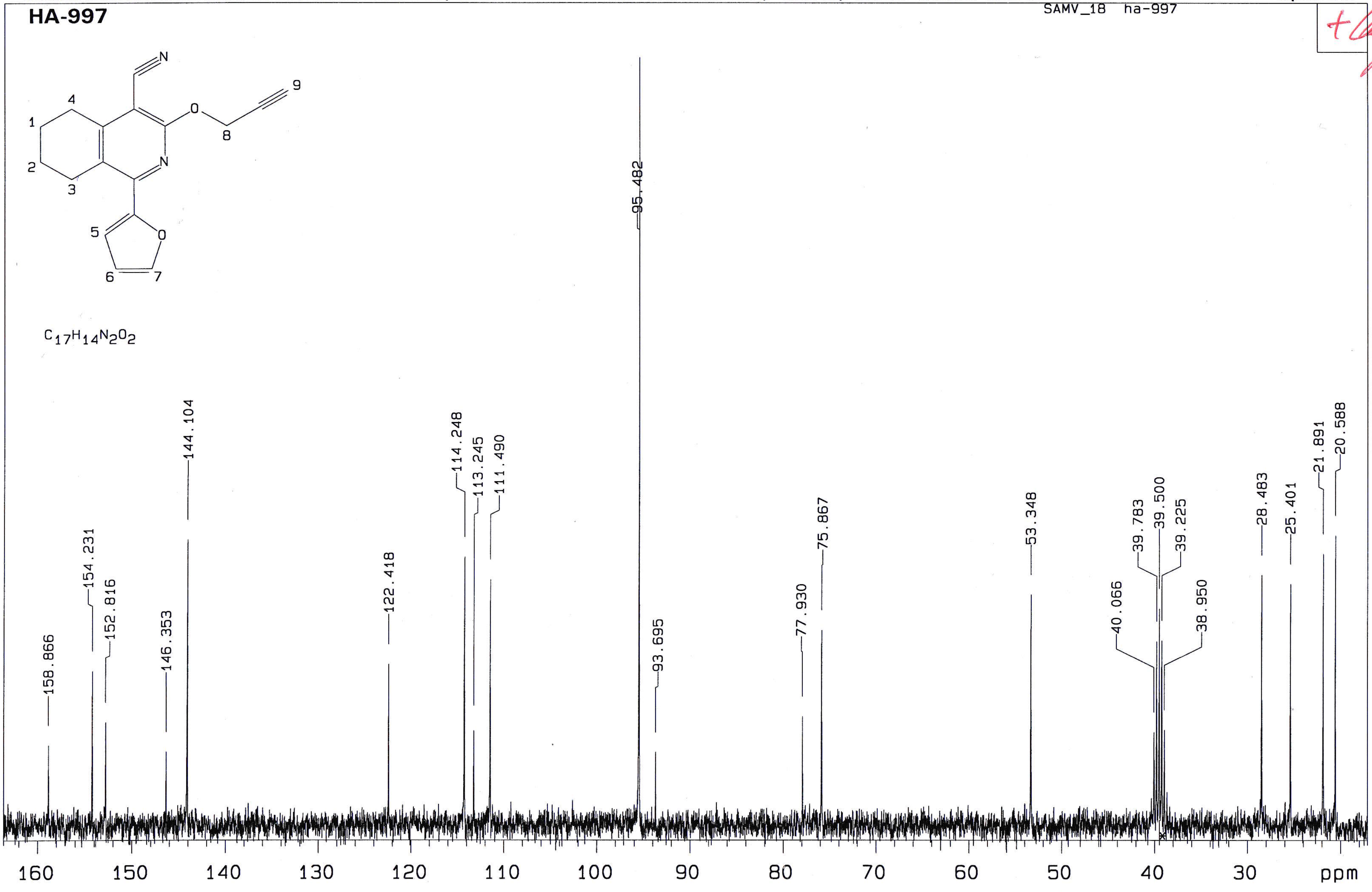

2f

Molecular Structure Research Centre, Yerevan, Armenia, Varian Mercury-300VX

H1 300.088 MHz, nt=16, np=32000, temp=30.0 C, lb=-0.2, solvent=DMSO/CCl4 1/3

Feb 27 2019

HA-1017

SAMV_19 ha-1017

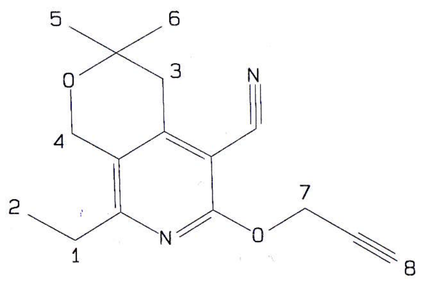

$C_{16}H_{18}N_2O_2$

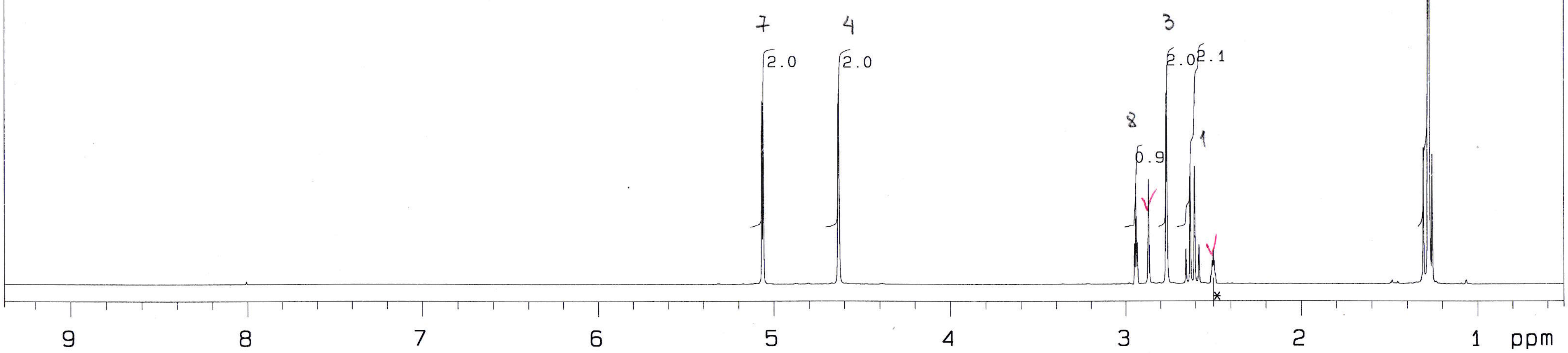

Molecular Structure Research Centre, Yerevan, Armenia, Varian Mercury-300VX

C13 75.465 MHz, nt = 288, np = 19998, temp = 30.0 C, lb = 1.0, solvent = DMSO/CCl4 1/3

Feb 27 2019

HA-1017

SAMV_19 ha-1017

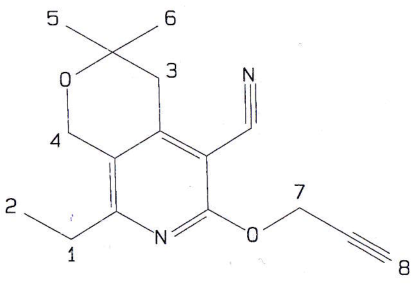

$C_{16}H_{18}N_2O_2$

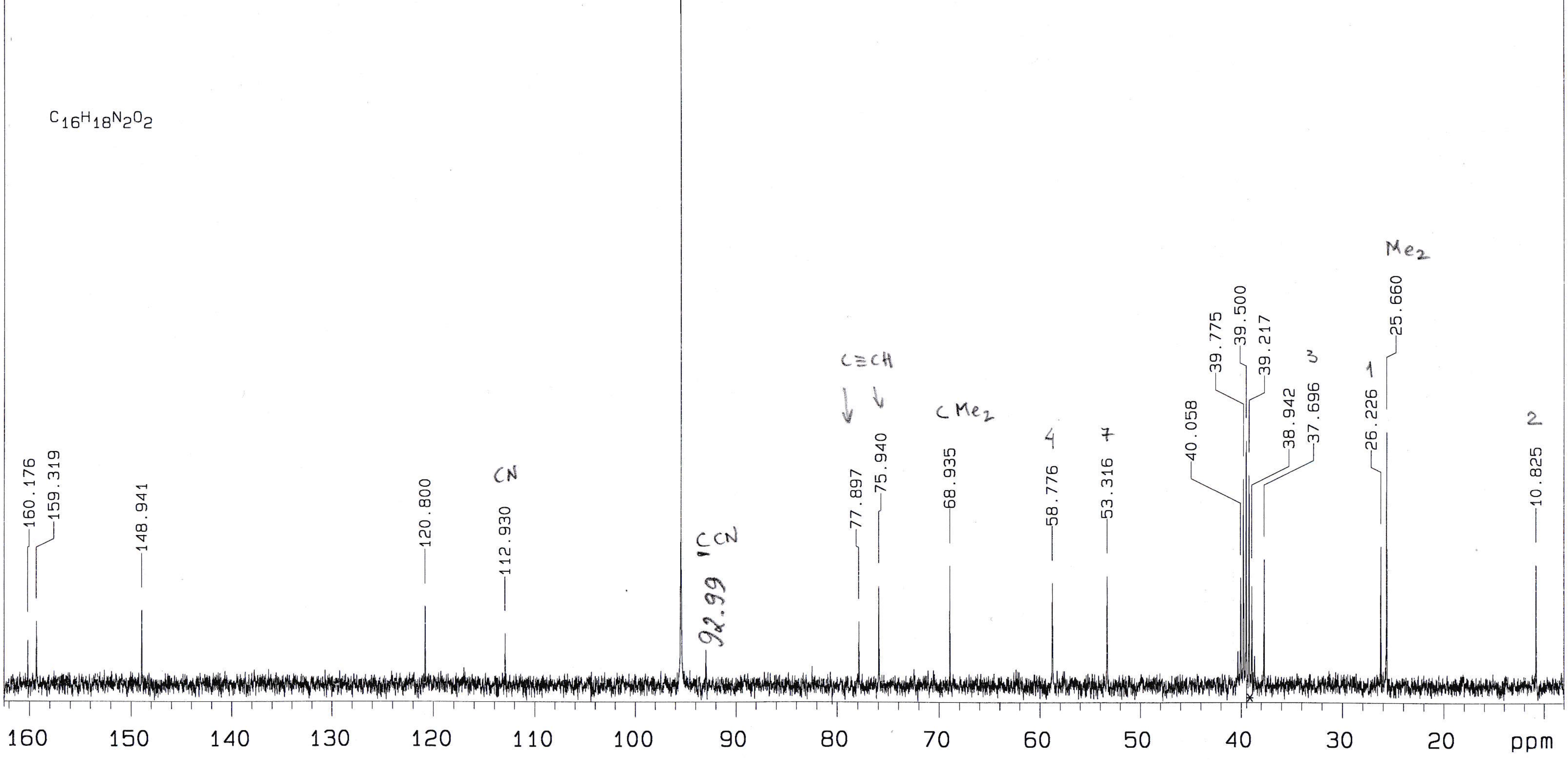

2f

2g

Molecular Structure Research Centre, Yerevan, Armenia, Varian Mercury-300VX

H1 300.088 MHz, nt=16, np=32000, temp=30.0 C, lb=-0.2, solvent=DMSO/CCl4 1/3

Mar 25 2019

HA-1023

SAMV_19 ha-1023

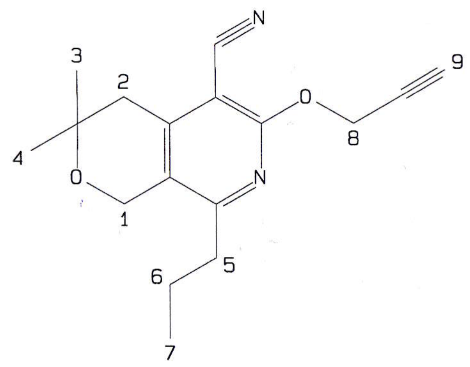

$C_{17}H_{20}N_2O_2$

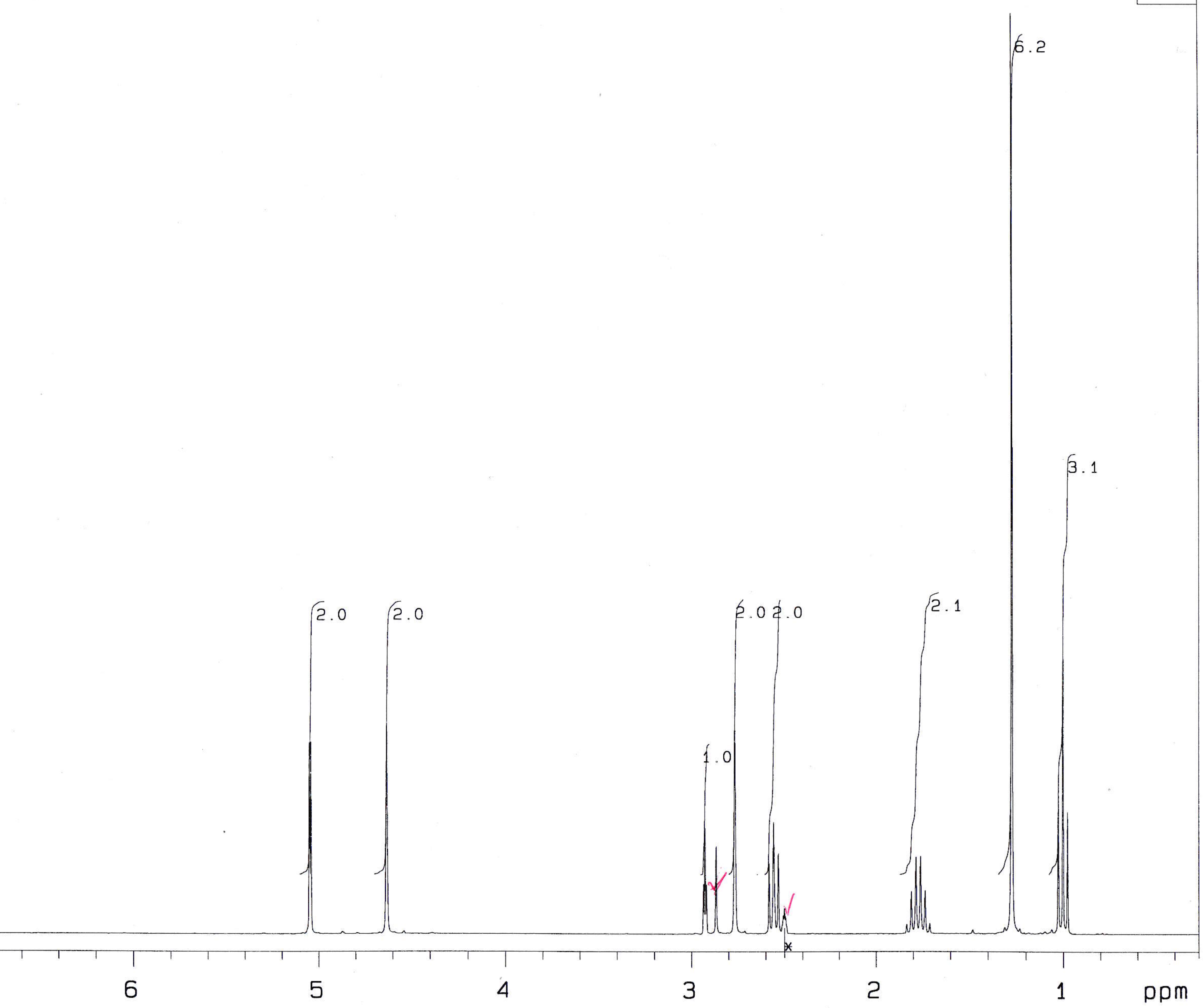

2g

Molecular Structure Research Centre, Yerevan, Armenia, Varian Mercury-300VX **C13 75.465 MHz, nt=256, np=32000, temp=30.0 C, lb=1.0, solvent=DMSO/CCl4 1/3** **Mar 25 2019**

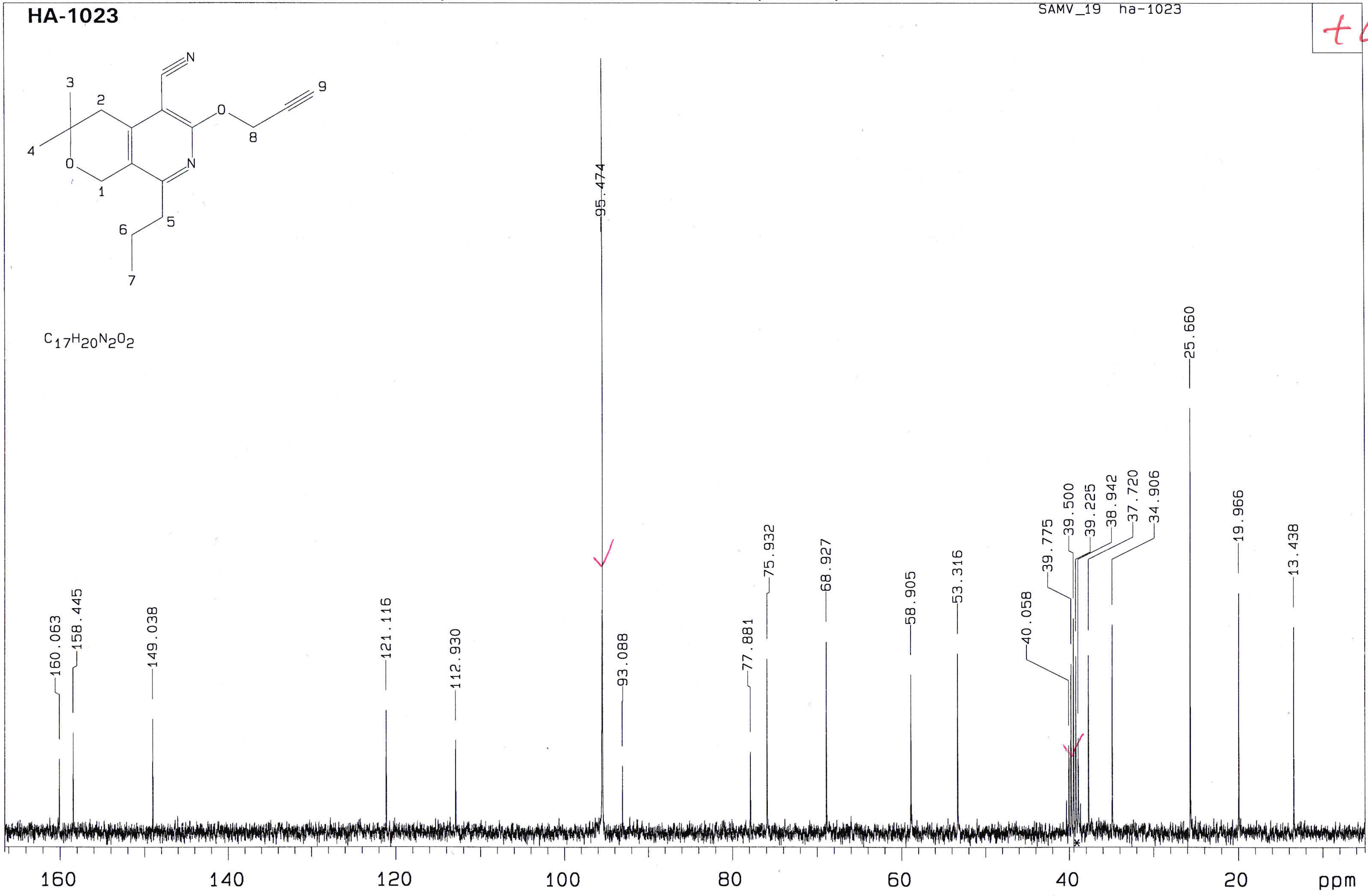

+

2k

Molecular Structure Research Centre, Yerevan, Armenia, Varian Mercury-300VX

H1 300.088 MHz, nt = 16, np = 32000, temp = 30.0 C, lb = -0.2, solvent = DMSO/CCl4 1/3

May 5 2021

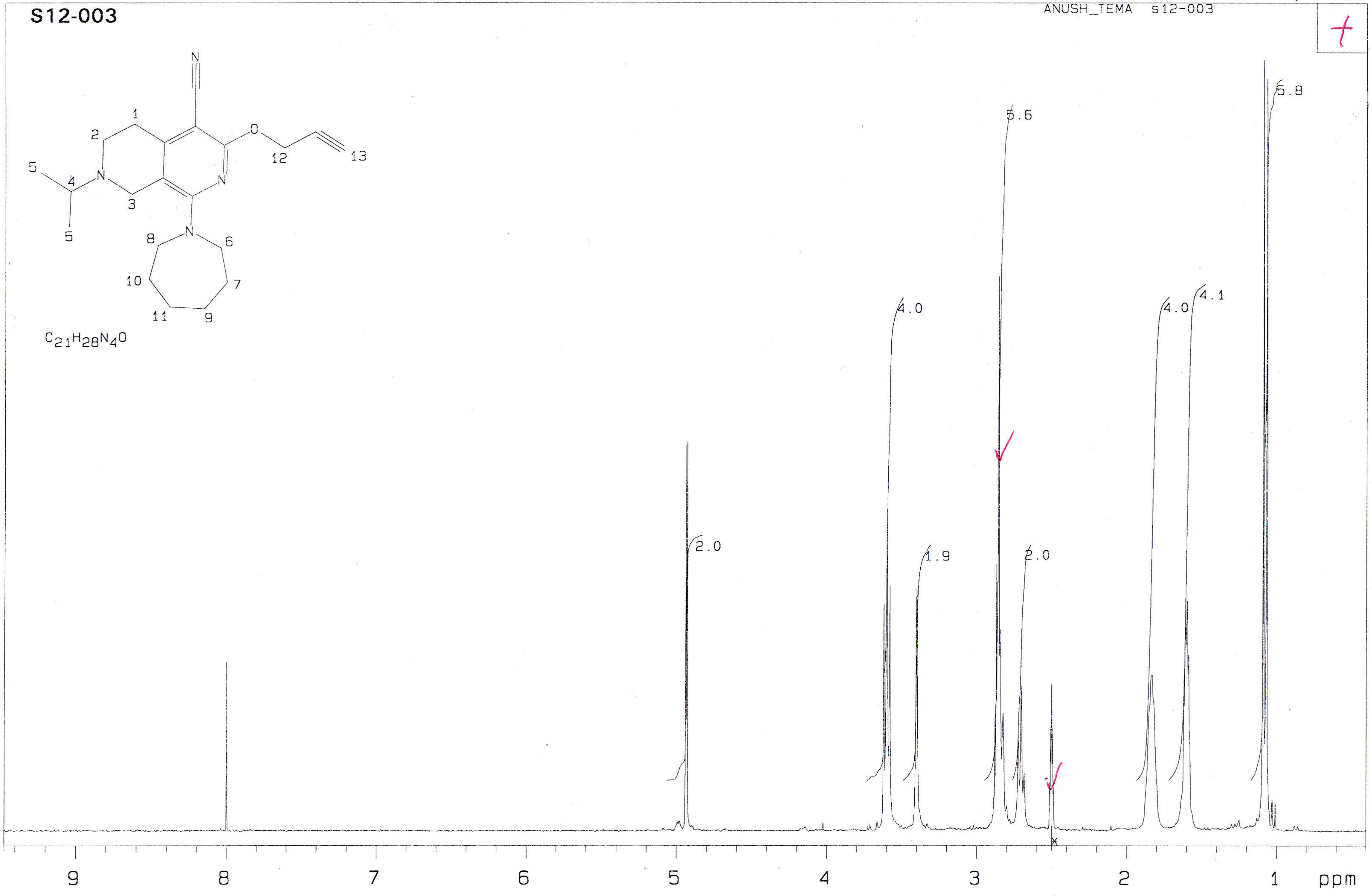

2h

Molecular Structure Research Centre, Yerevan, Armenia, Varian Mercury-300VX

C13 75.465 MHz, nt=800, np=19998, temp=30.0 C, lb=1.0, solvent=DMSO/CCl4 1/3

May 6 2021

S12-003

ANUSH_TEMA s12-003

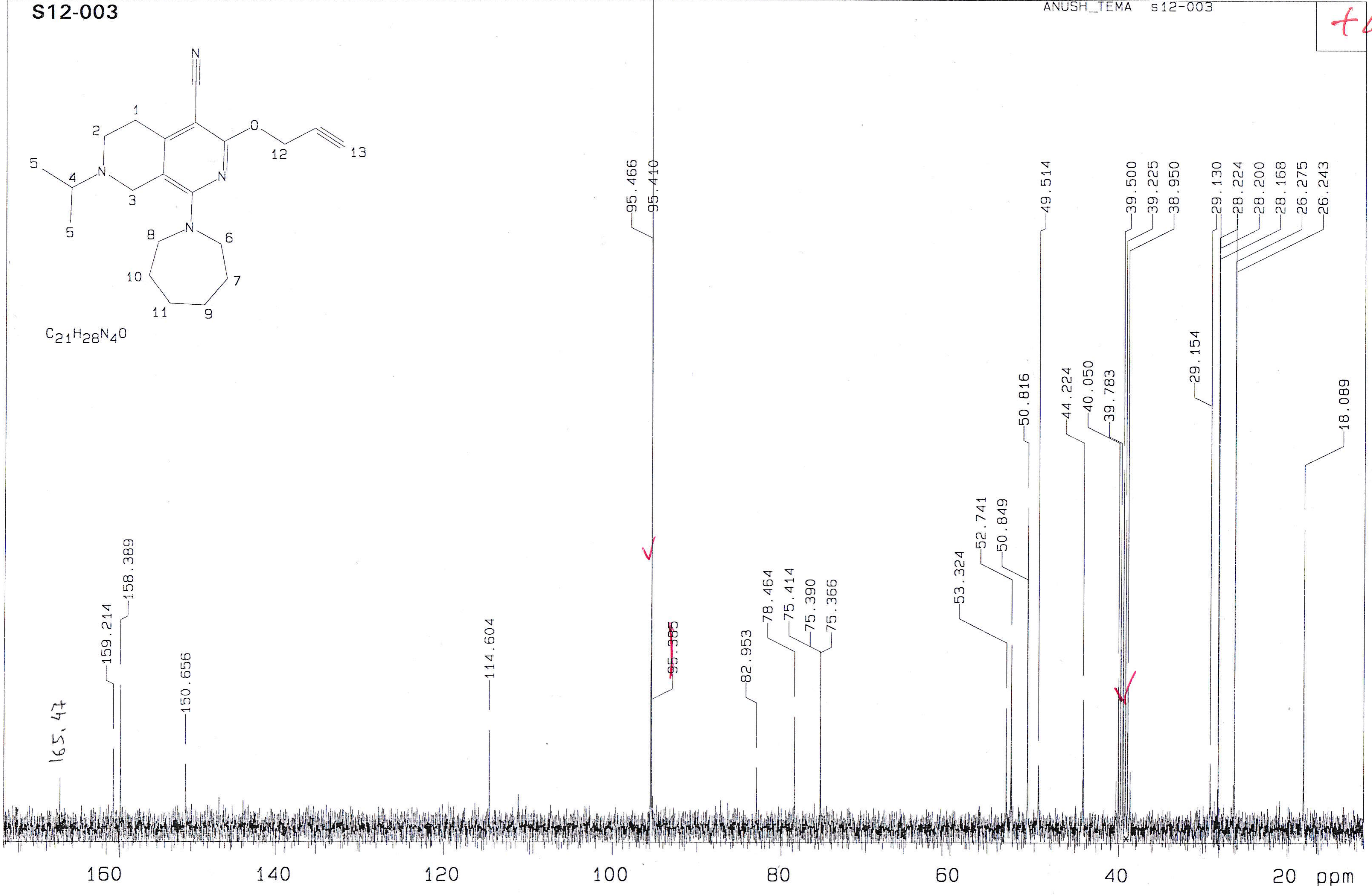

2i

Molecular Structure Research Centre, Yerevan, Armenia, Varian Mercury-300VX

H1 300.088 MHz, nt = 16, np = 32000, temp = 30.0 C, lb = -0.2, solvent = DMSO/CCl4 1/3

Aug 5 2021

S12-030

NOCI_21 s12-030

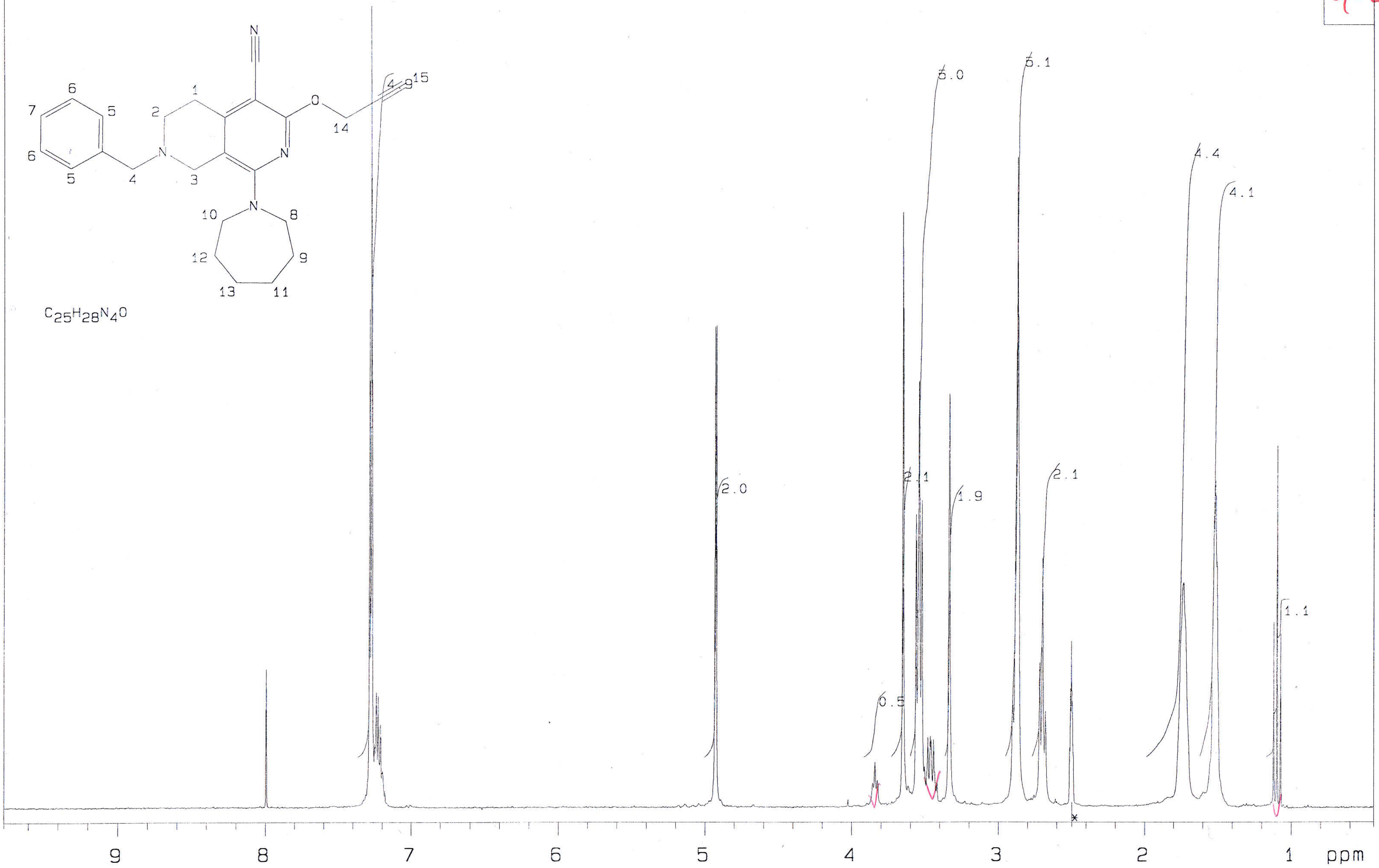

2i

Molecular Structure Research Centre, Yerevan, Armenia, Varian Mercury-300VX

C13 75.465 MHz, nt=624, np=19998, temp=30.0 C, lb=1.0, solvent=DMSO/CCl4 1/3

Aug 5 2021

NOCI_21 s12-030

S12-030

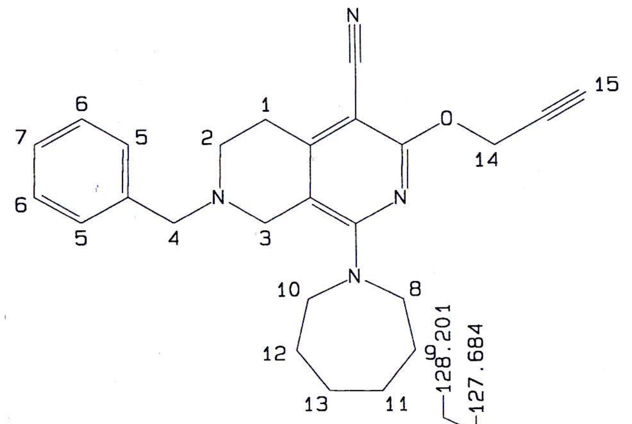

$C_{25}H_{28}N_4O$

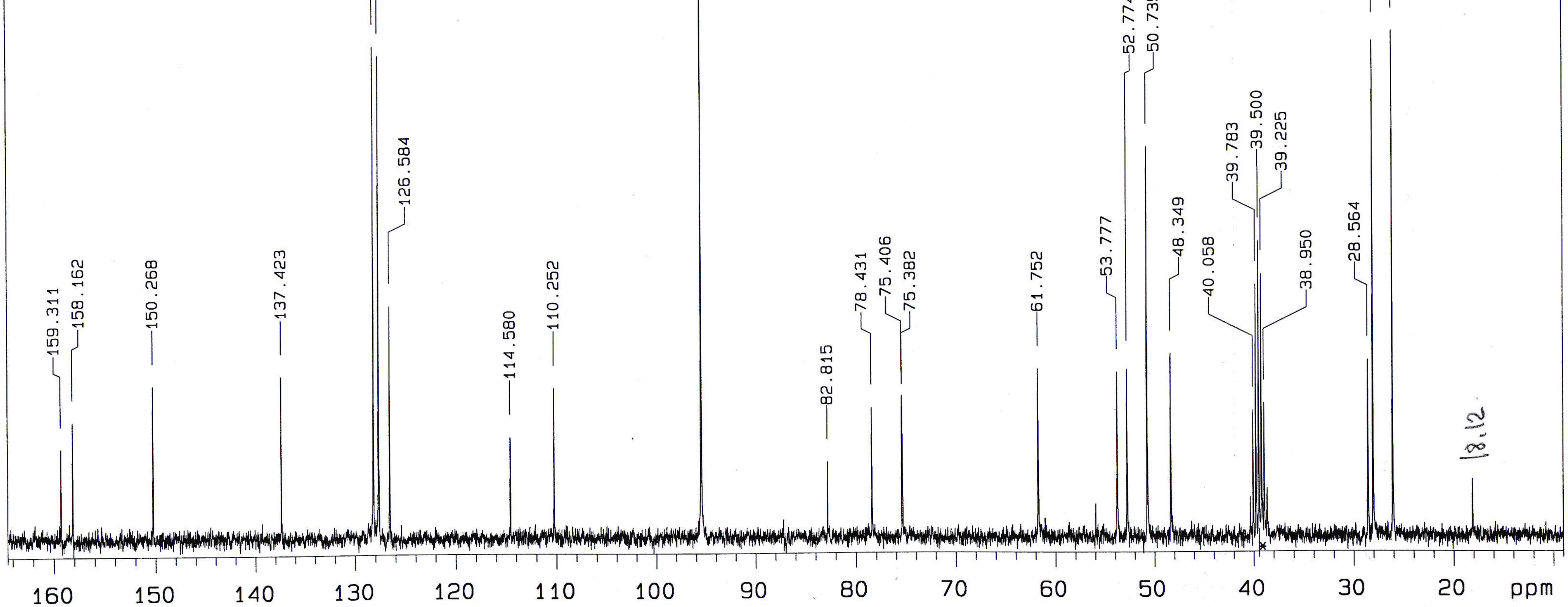

3f

Molecular Structure Research Centre, Yerevan, Armenia, Varian Mercury-300VX

H1 300.088 MHz, nt=16, np=32000, temp=30.0 C, lb=-0.2, solvent=DMSO/CCl4 1/3

Apr 22 2019

SAMV_19 ha-1017-1

HA-1017-1

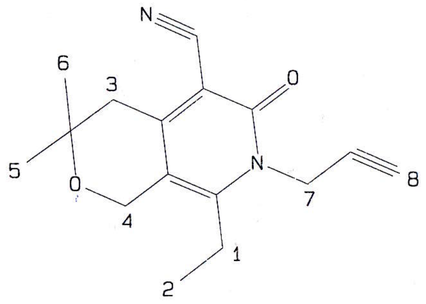

$C_{16}H_{18}N_2O_2$

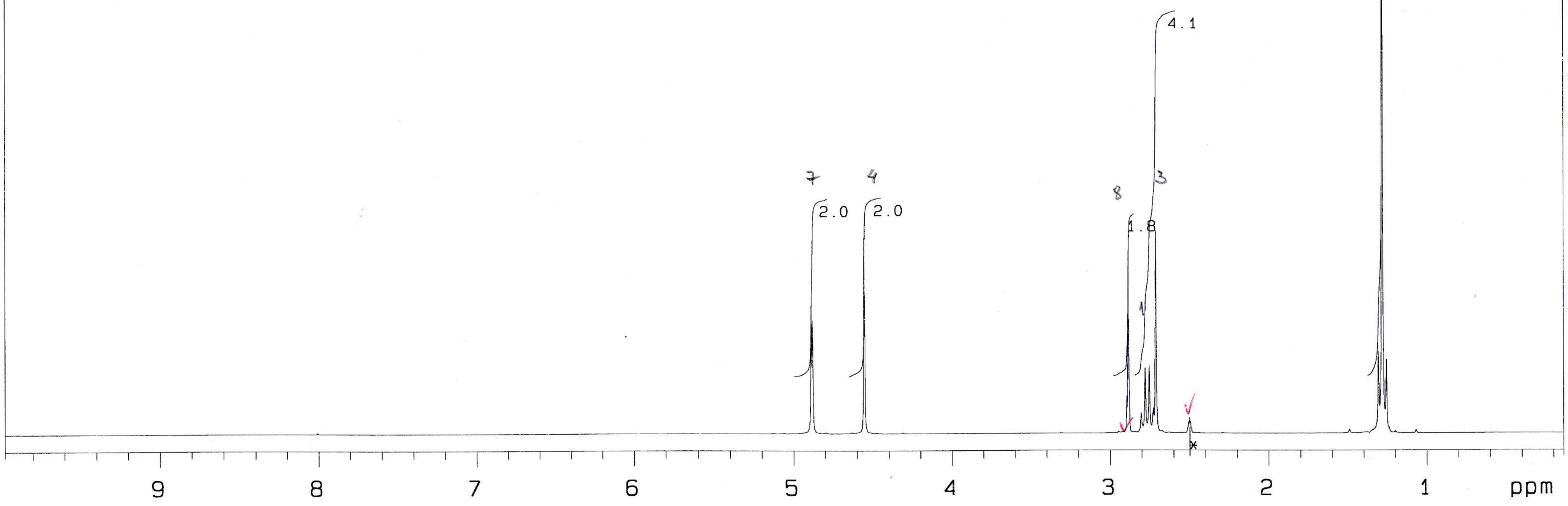

3f

Molecular Structure Research Centre, Yerevan, Armenia, Varian Mercury-300VX **C13 75.465 MHz, nt = 256, np = 19998, temp = 30.0 C, lb = 1.0, solvent = DMSO/CCl4 1/3** **Apr 22 2019**

HA-1017-1

SAMV_19 ha-1017-1

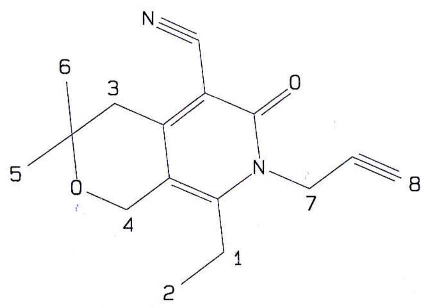

$C_{16}H_{18}N_2O_2$

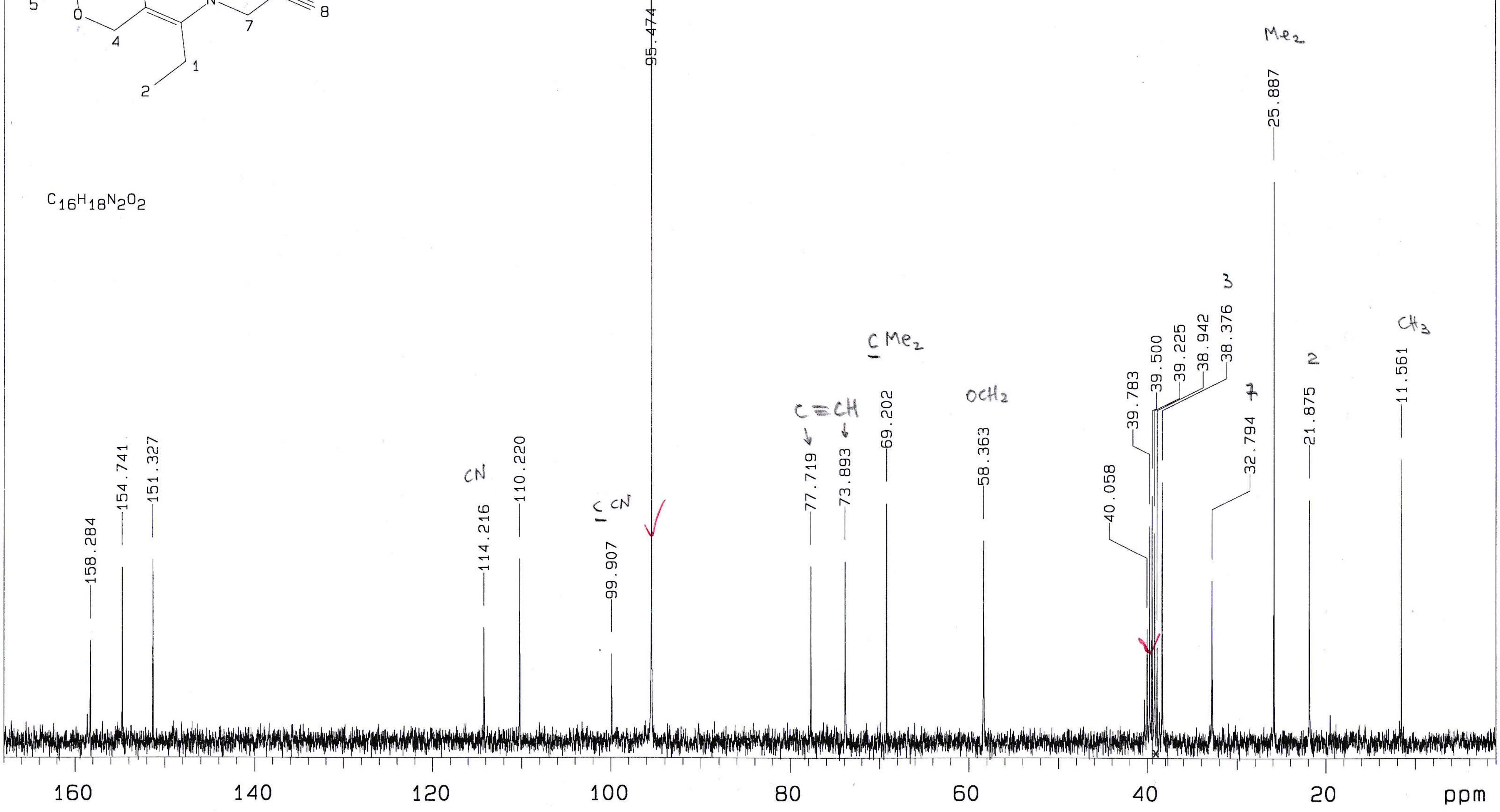

5a

Molecular Structure Research Centre, Yerevan, Armenia, Varian Mercury-300VX H1 300.088 MHz, nt = 16, np = 32000, temp = 30.0 C, lb = -0.2, solvent = DMSO/CCl4 1/3 Mar 13 2019

SAMV_19 he-439

HE-439

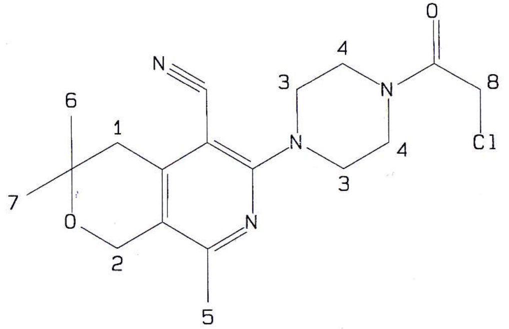

$C_{18}H_{23}ClN_4O_2$

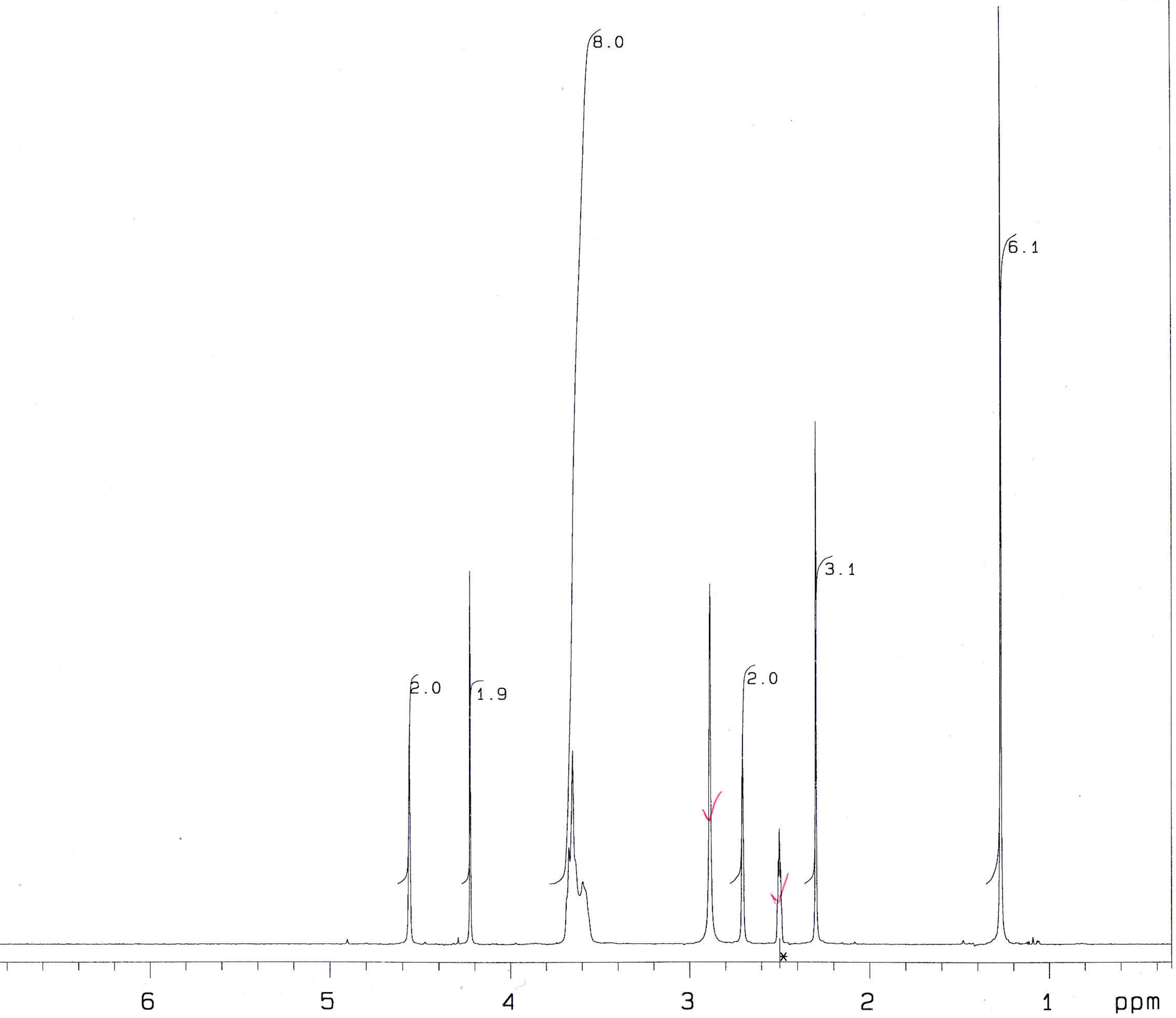

5a

Molecular Structure Research Centre, Yerevan, Armenia, Varian Mercury-300VX

C13 75.465 MHz, nt=432, np=19998, temp=30.0 C, lb=1.0, solvent=DMSO/CCl4 1/3

Mar 13 2019

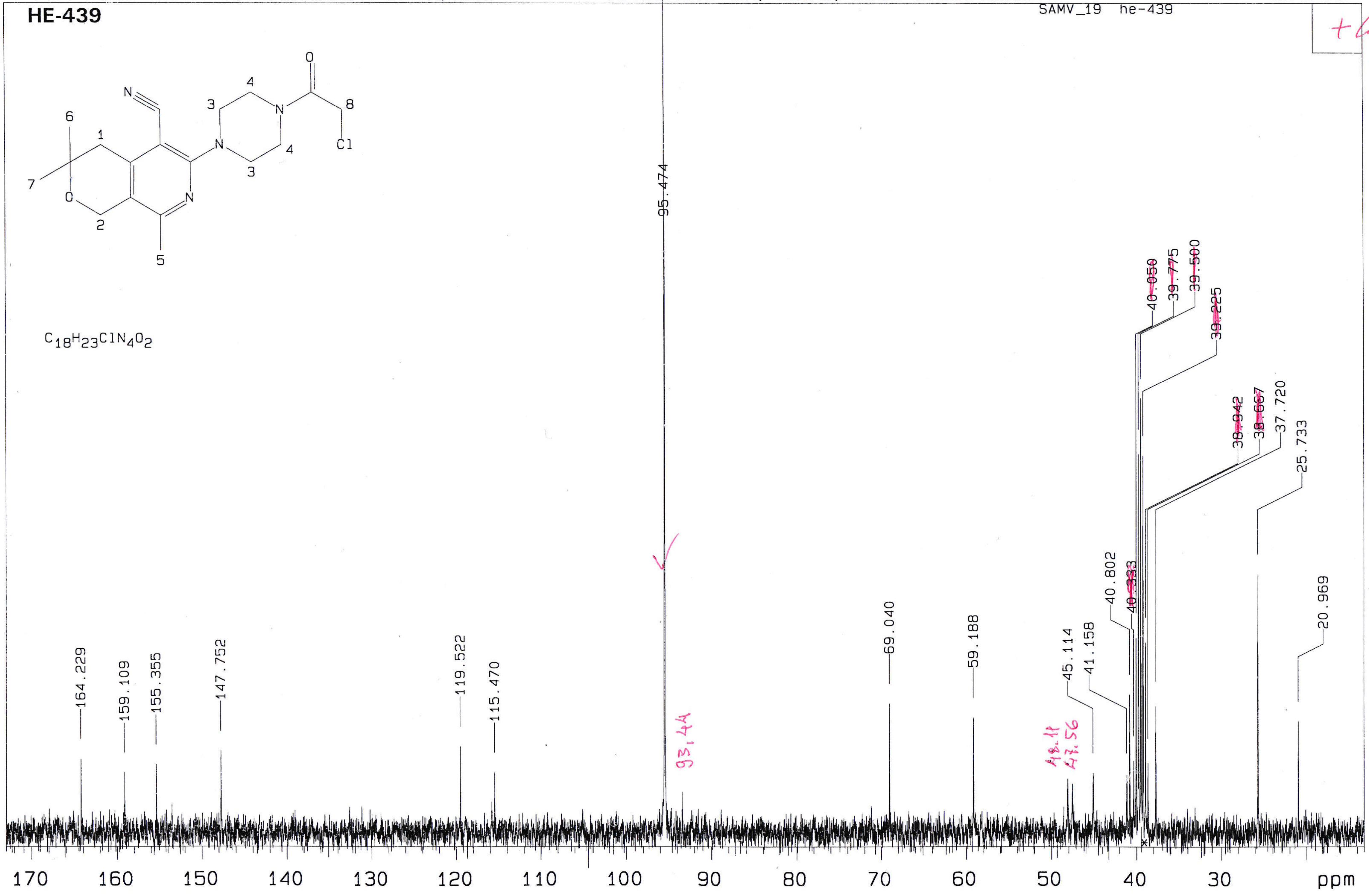

6a

Molecular Structure Research Centre, Yerevan, Armenia, Varian Mercury-300VX **H1 300.088 MHz, nt = 16, np = 32000, temp = 30.0 C, lb = -0.2, solvent = DMSO/CCl4 1/3** **Mar 29 2019**

SAMV_19 ha-1028

HA-1028

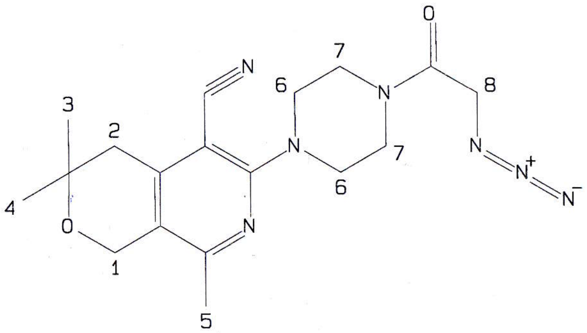

$C_{18}H_{23}N_7O_2$

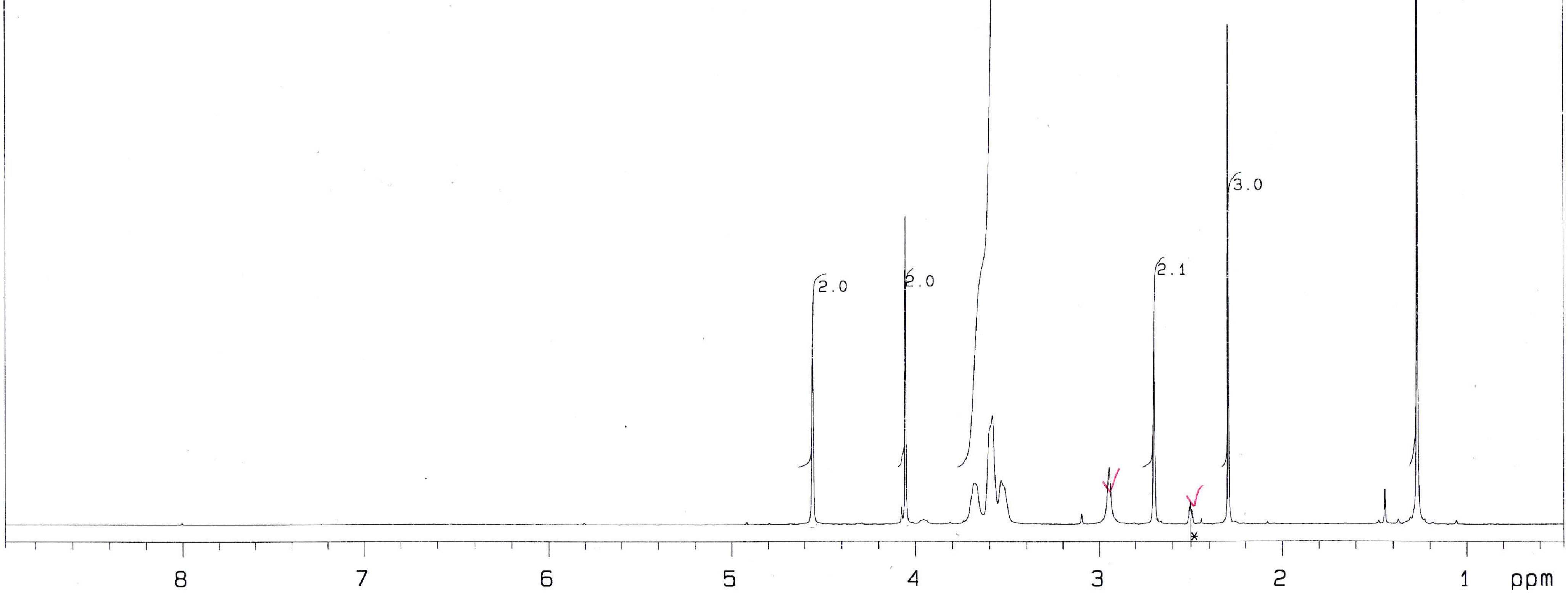

6a

Molecular Structure Research Centre, Yerevan, Armenia, Varian Mercury-300VX

C13 75.465 MHz, nt=128, np=19998, temp=30.0 C, lb=1.0, solvent=DMSO/CCl4 1/3

Mar 29 2019

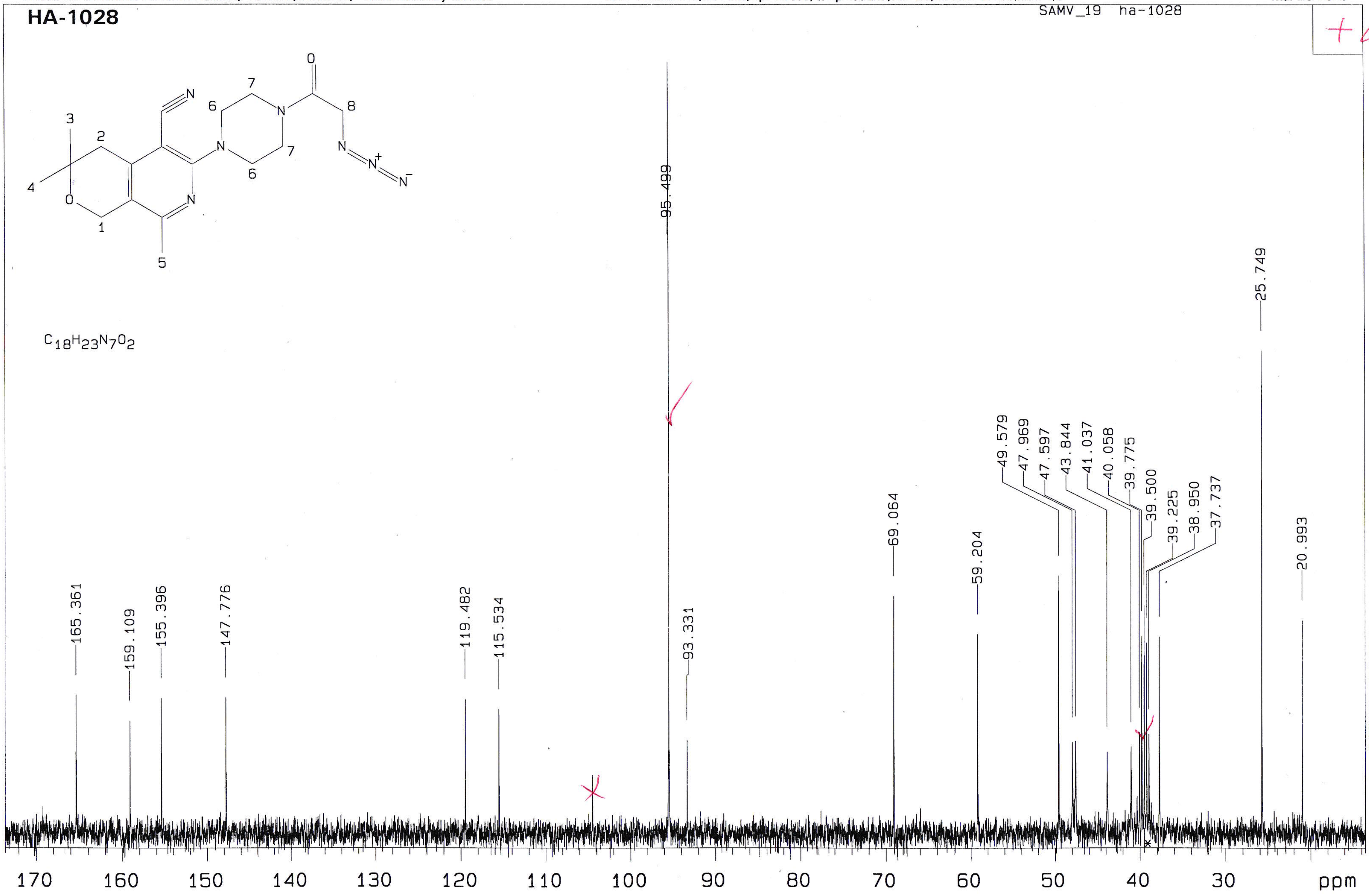

7ca

Molecular Structure Research Centre, Yerevan, Armenia, Varian Mercury-300VX

H1 300.088 MHz, nt=16, np=32000, temp=30.0 C, lb=-0.2, solvent=DMSO/CCl4 1/3

Apr 22 2019

SAMV_19 ha-1035

HA-1035

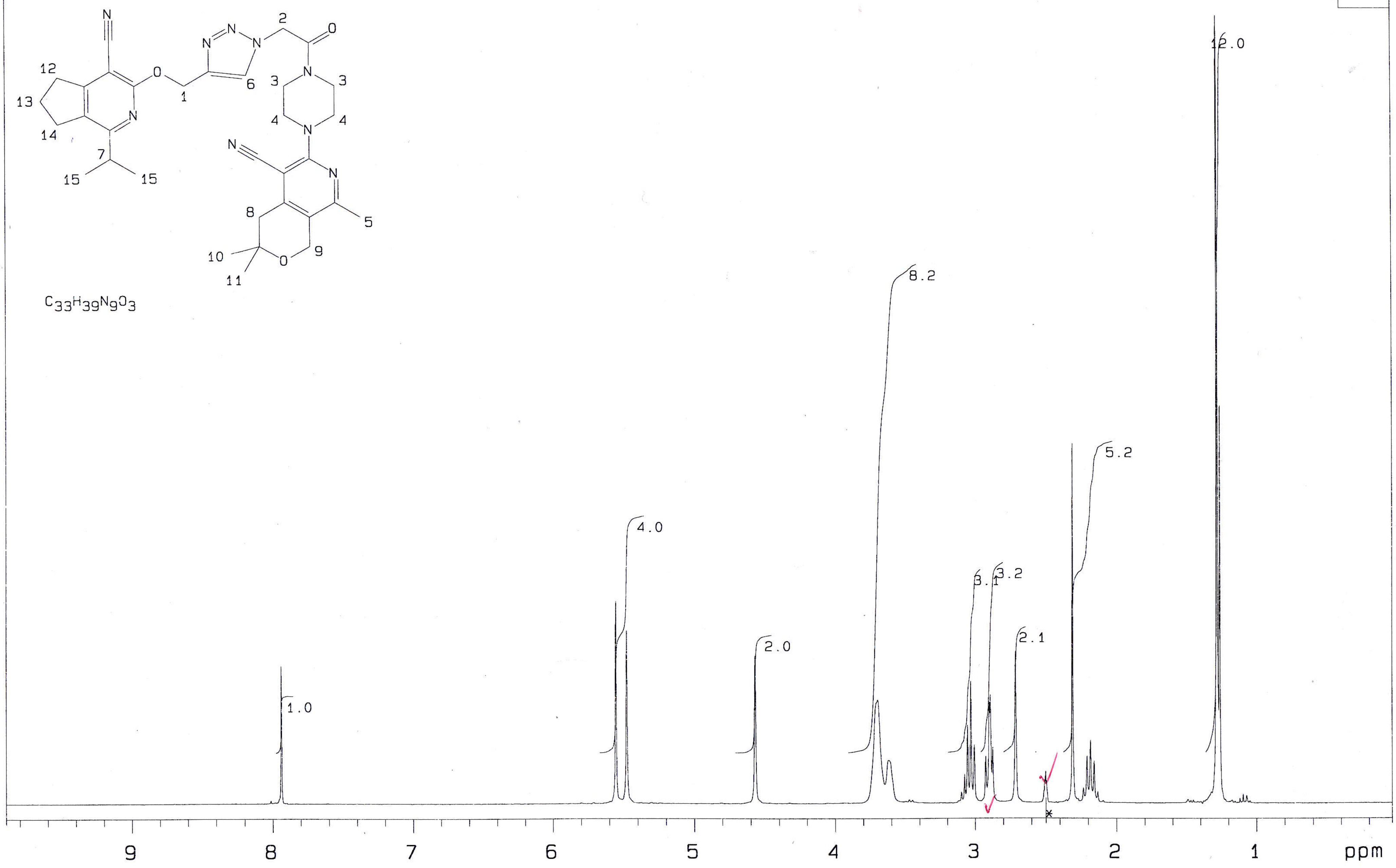

7a

Molecular Structure Research Centre, Yerevan, Armenia, Varian Mercury-300VX

C13 75.465 MHz, nt=416, np=19998, temp=30.0 C, lb=1.0, solvent=DMSO/CCl4 1/3

Apr 22 2019

SAMV_19 ha-1035

HA-1035

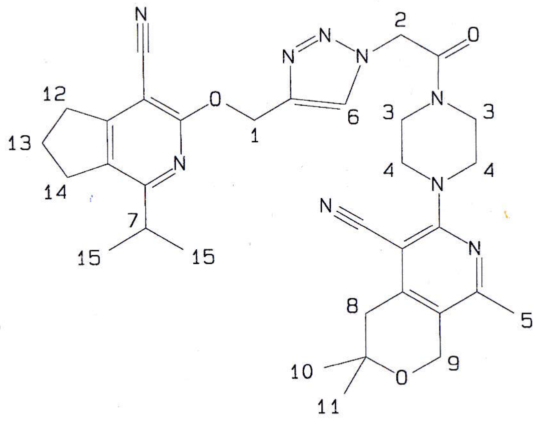

$C_{33}H_{39}N_9O_3$

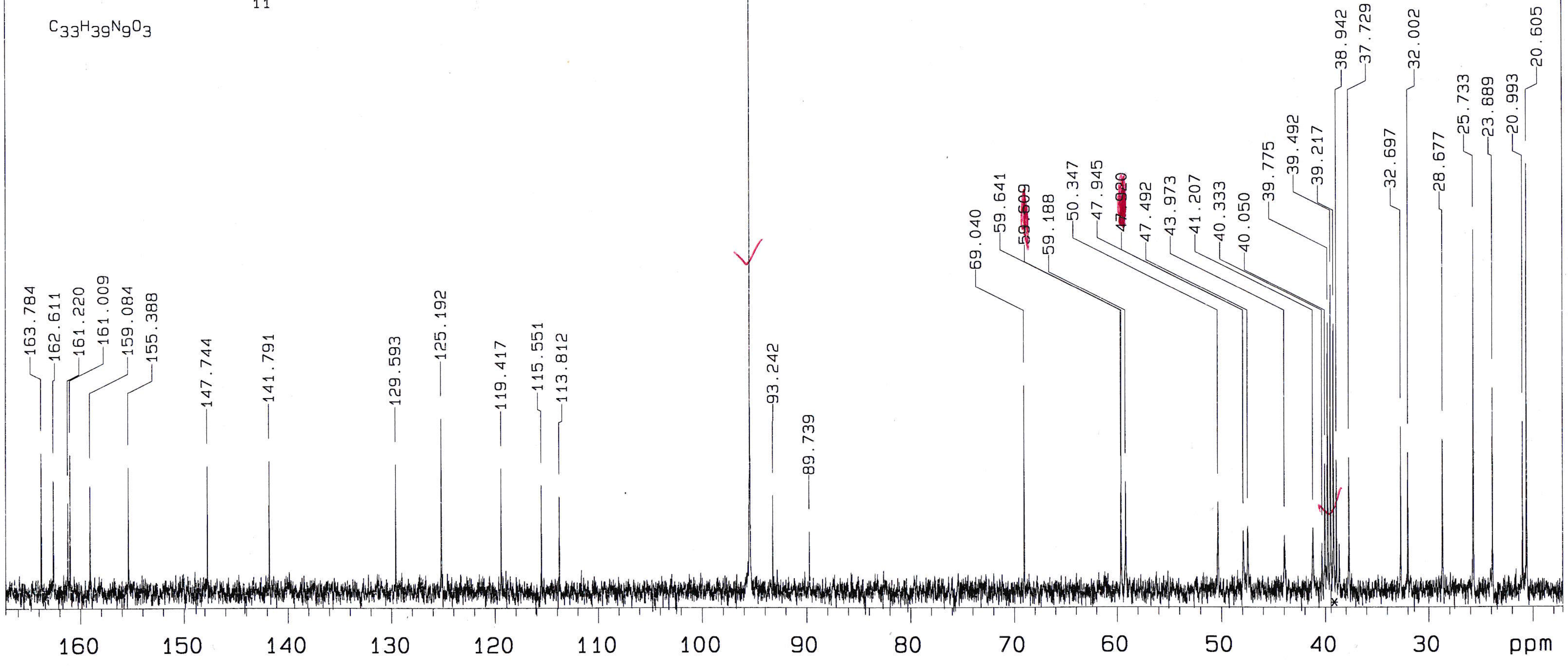

7a

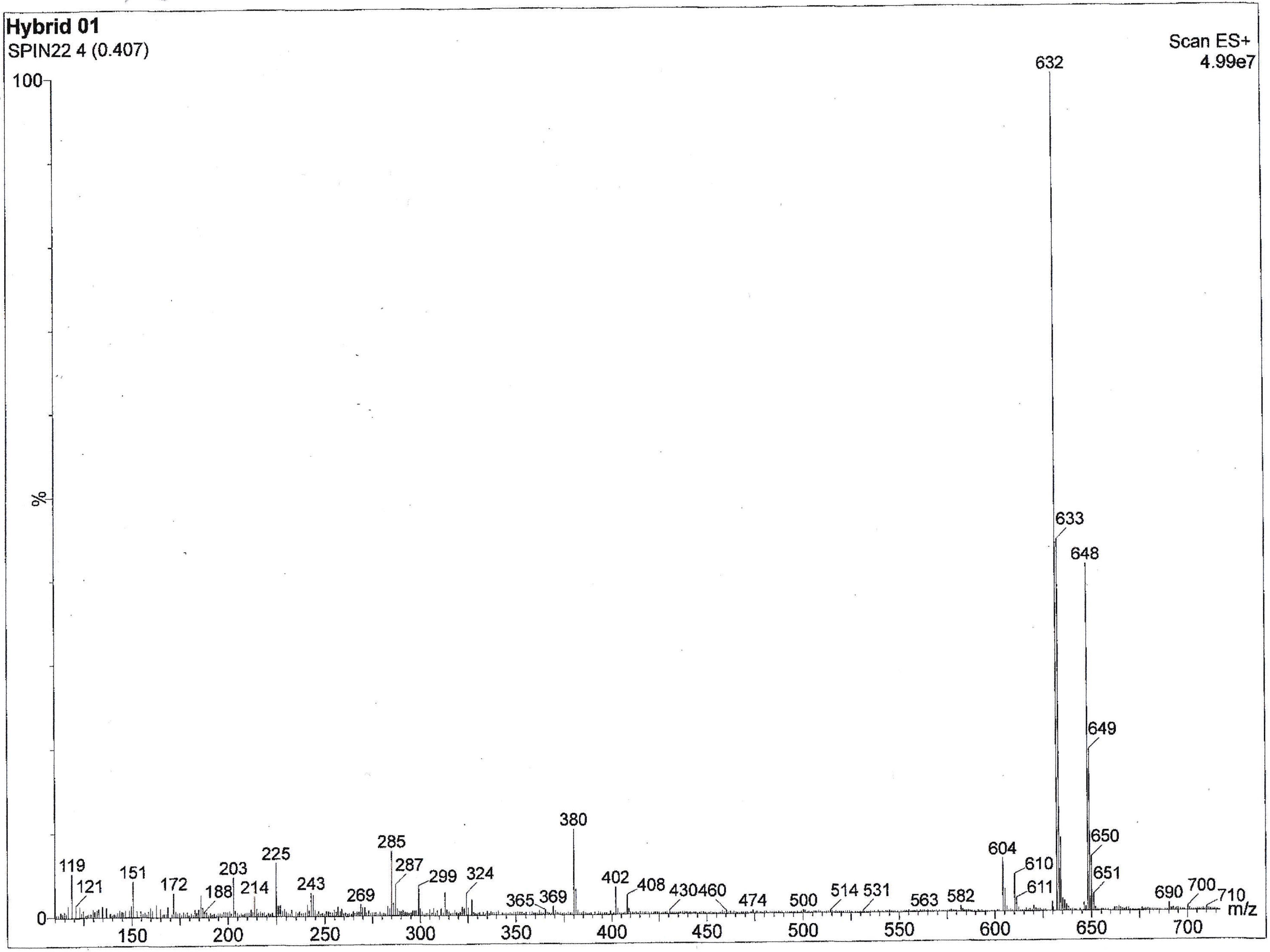
Hybrid 01
SPIN22 4 (0.407)
Scan ES+
4.99e7
100
%
0
632
633
648
649
650
651
604
610
611
380
285
287
299
324
225
243
269
119
121
151
172
188
203
214
365
369
402
408
430460
474
500
514 531
563
582
690
700
710
150
200
250
300
350
400
450
500
550
600
650
700
m/z

7a

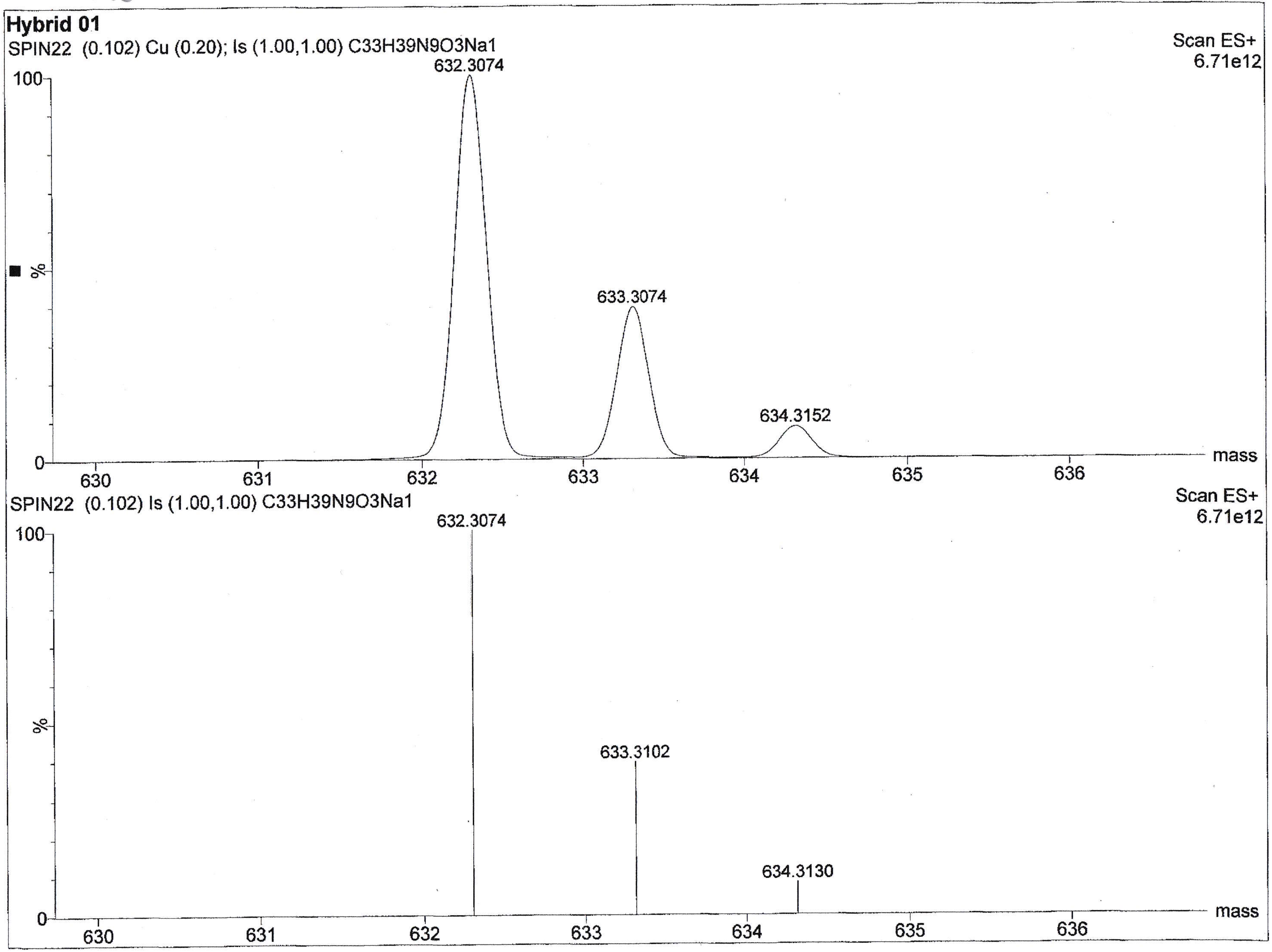
Hybrid 01
SPIN22 (0.102) Cu (0.20); Is (1.00,1.00) C33H39N9O3Na1
Scan ES+
6.71e12
632.3074
633.3074
634.3152
100
%
0
630
631
632
633
634
635
636
mass
SPIN22 (0.102) Is (1.00,1.00) C33H39N9O3Na1
Scan ES+
6.71e12
632.3074
633.3102
634.3130
100
%
0
630
631
632
633
634
635
636
mass

7c

Molecular Structure Research Centre, Yerevan, Armenia, Varian Mercury-300VX

H1 300.088 MHz, nt=16, np=32000, temp=30.0 C, lb=-0.2, solvent=DMSO/CCl4 1/3

Mar 28 2019

HA-1026

SAMV_19 ha-1026

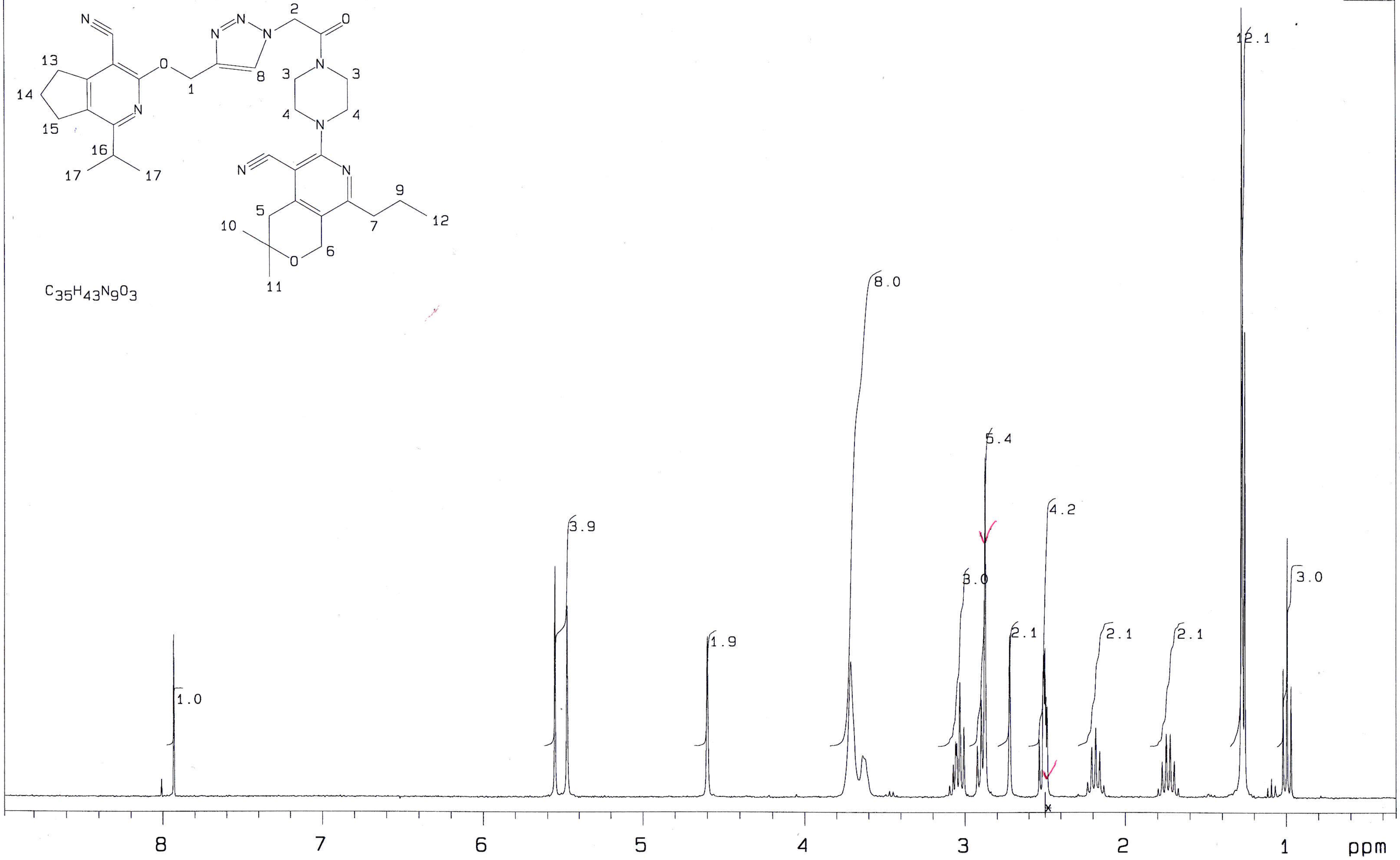

7c

Molecular Structure Research Centre, Yerevan, Armenia, Varian Mercury-300VX

C13 75.465 MHz, nt = 2544, np = 19998, temp = 30.0 C, lb = 1.0, solvent = DMSO/CCl4 1/3

Mar 28 2019

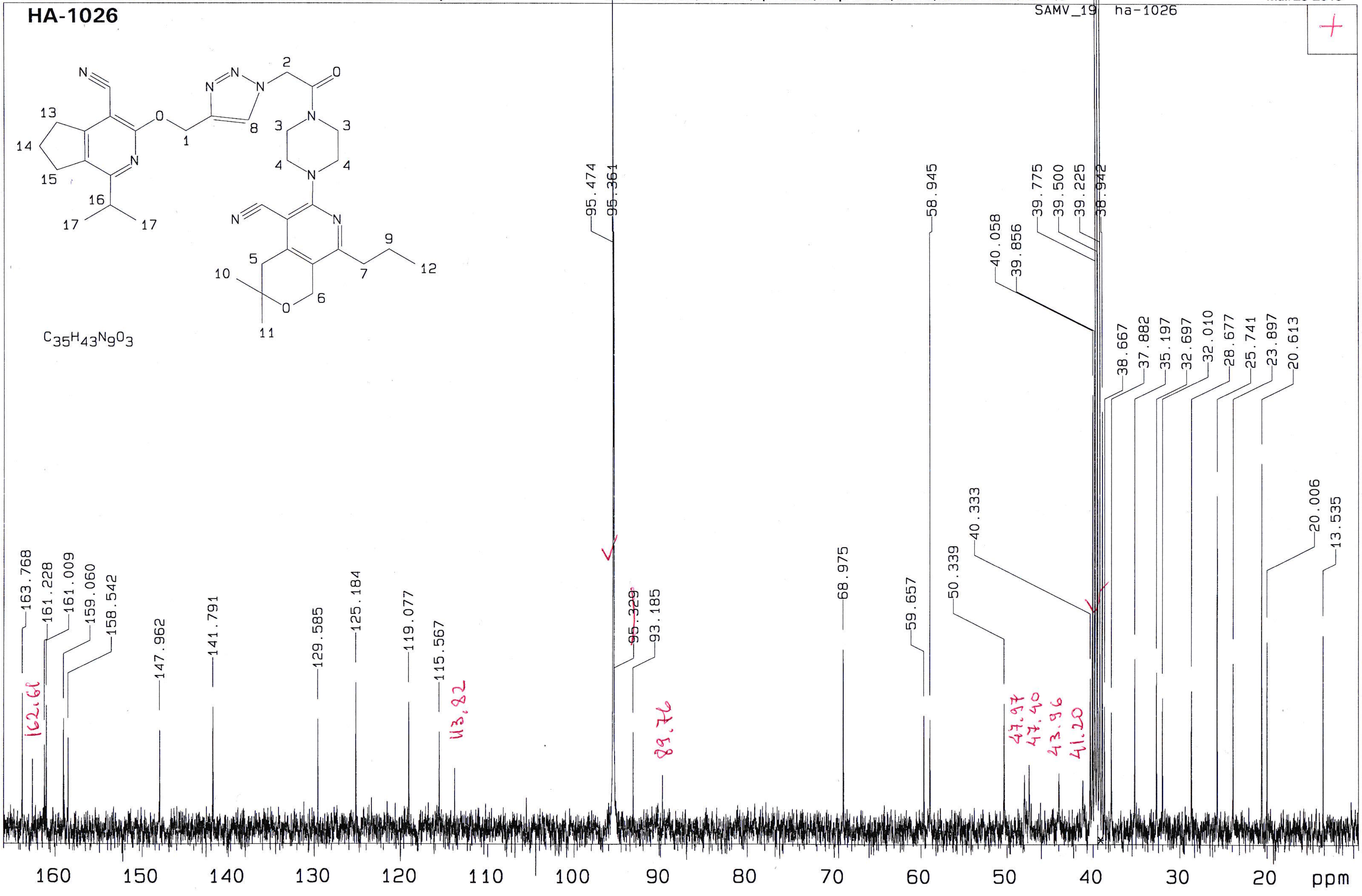

7c

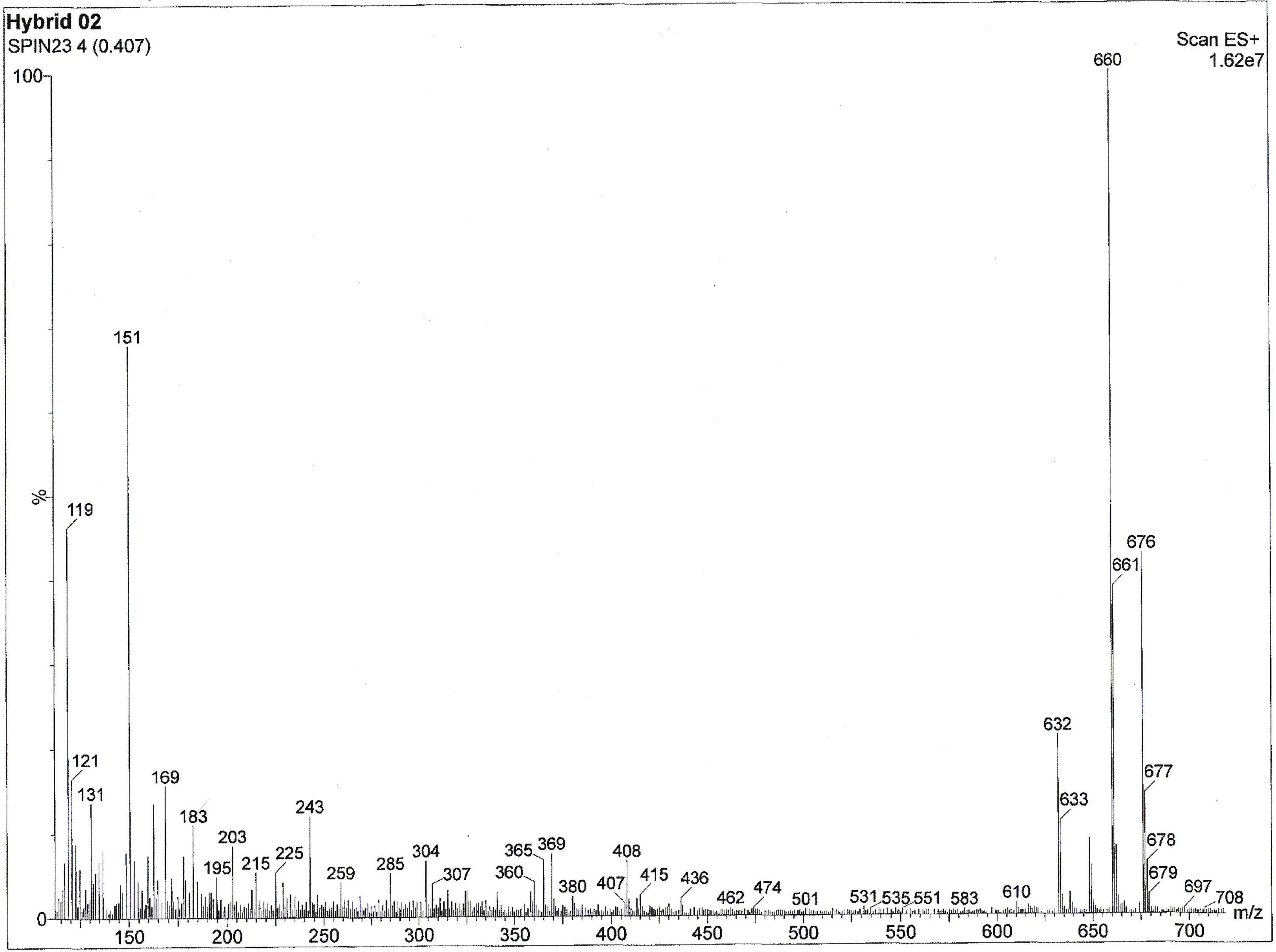
Hybrid 02
SPIN23 4 (0.407)
Scan ES+
1.62e7
100
%
0
119
121
131
151
169
183
195
203
215
225
243
259
285
304
307
360
365
369
380
407
408
415
436
462
474
501
531
535
551
583
610
632
633
660
661
676
677
678
679
697
708
150
200
250
300
350
400
450
500
550
600
650
700
m/z

7c

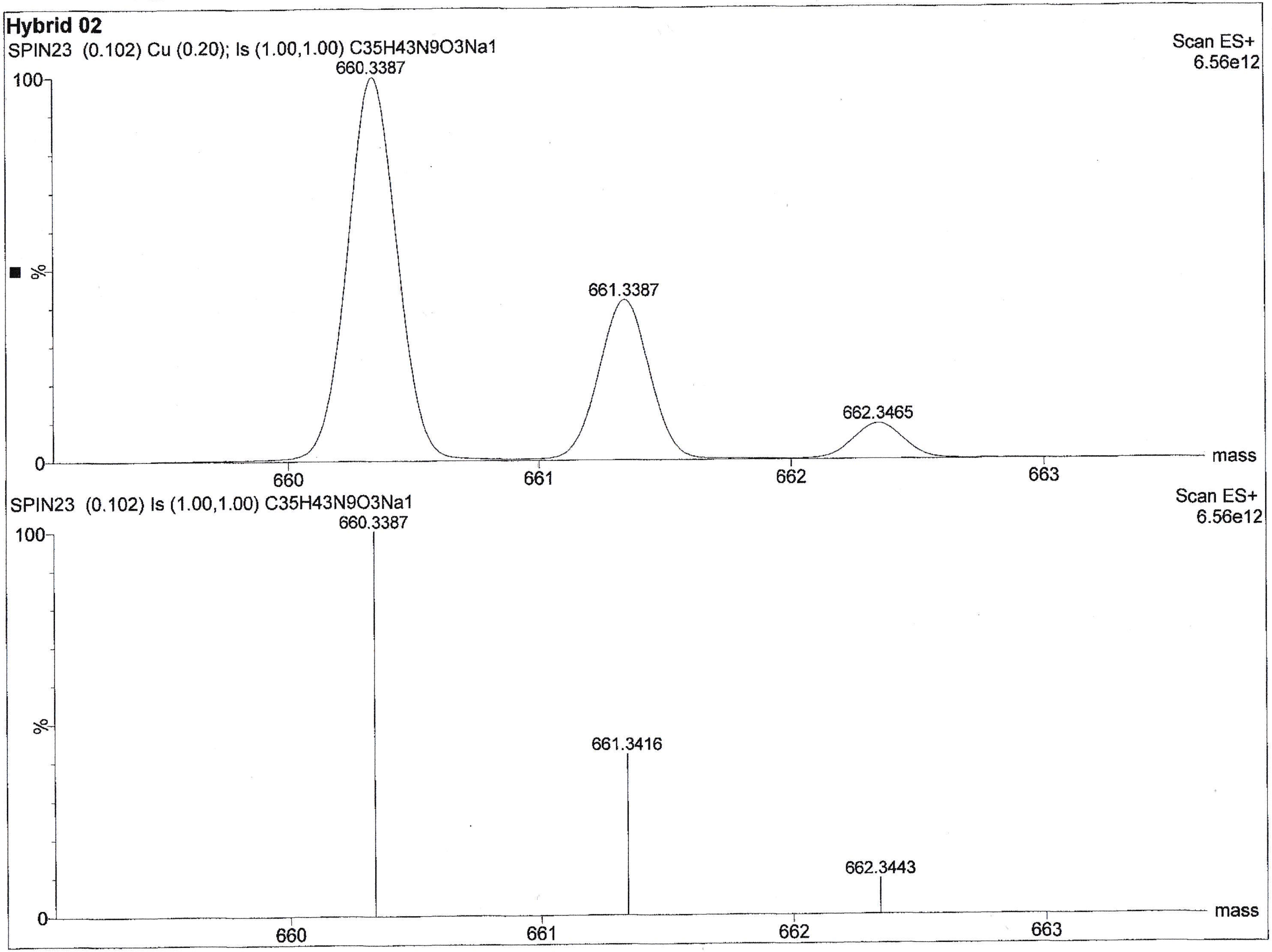
Hybrid 02
SPIN23 (0.102) Cu (0.20); Is (1.00,1.00) C35H43N9O3Na1
Scan ES+
6.56e12
660.3387
661.3387
662.3465
100
%
0
660
661
662
663
mass
SPIN23 (0.102) Is (1.00,1.00) C35H43N9O3Na1
Scan ES+
6.56e12
660.3387
661.3416
662.3443
100
%
0
660
661
662
663
mass

Molecular Structure Research Centre, Yerevan, Armenia, Varian Mercury-300VX

H1 300.088 MHz, nt = 16, np = 32000, temp = 30.0 C, lb = -0.2, solvent = DMSO/CCl4 1/3

Oct 22 2021

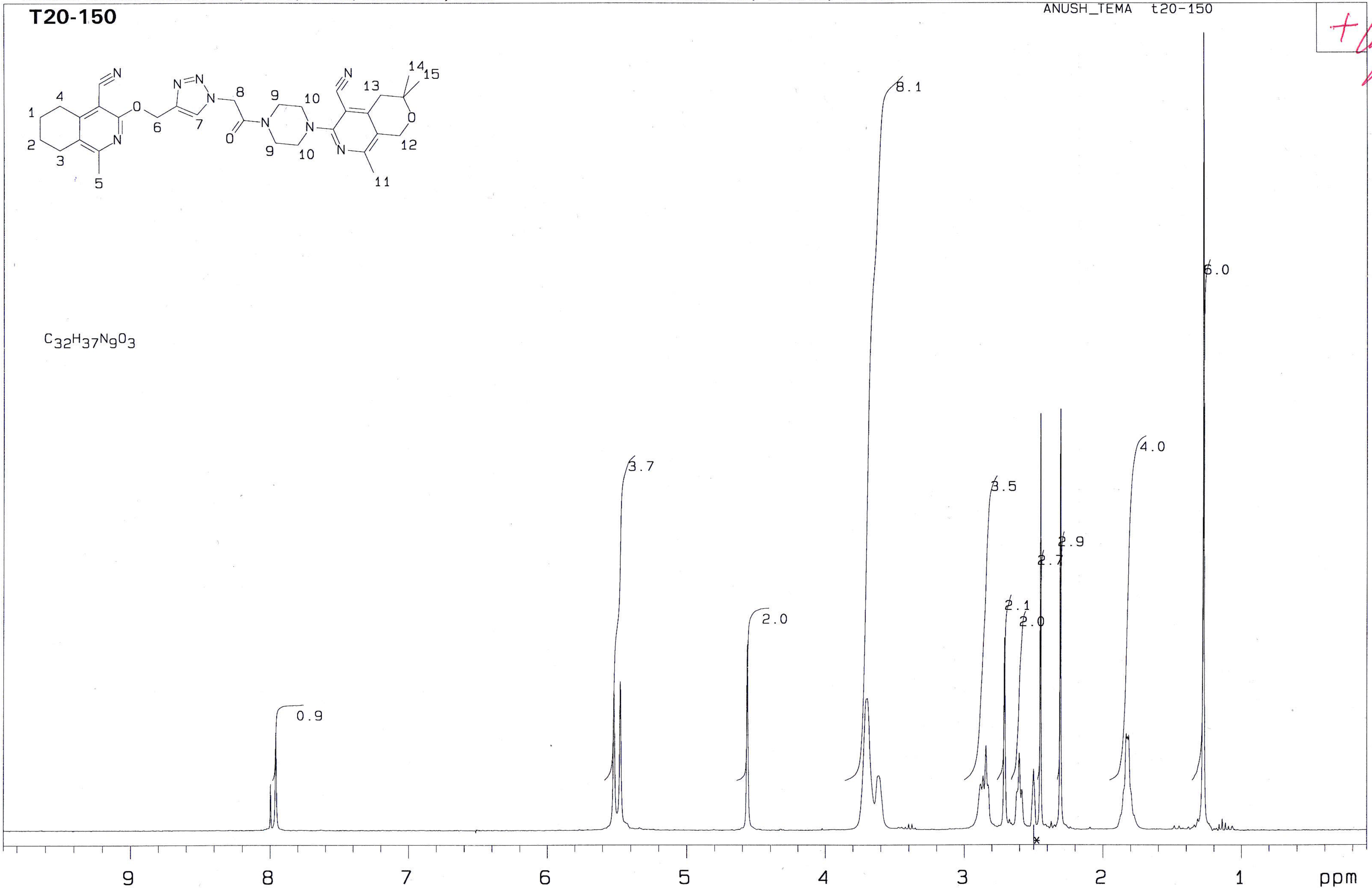

7d

Molecular Structure Research Centre, Yerevan, Armenia, Varian Mercury-300VX C13 75.465 MHz, nt = 1024, np = 19998, temp = 30.0 C, lb = 1.0, solvent = DMSO/CCl4 1/3 Oct 22 2021

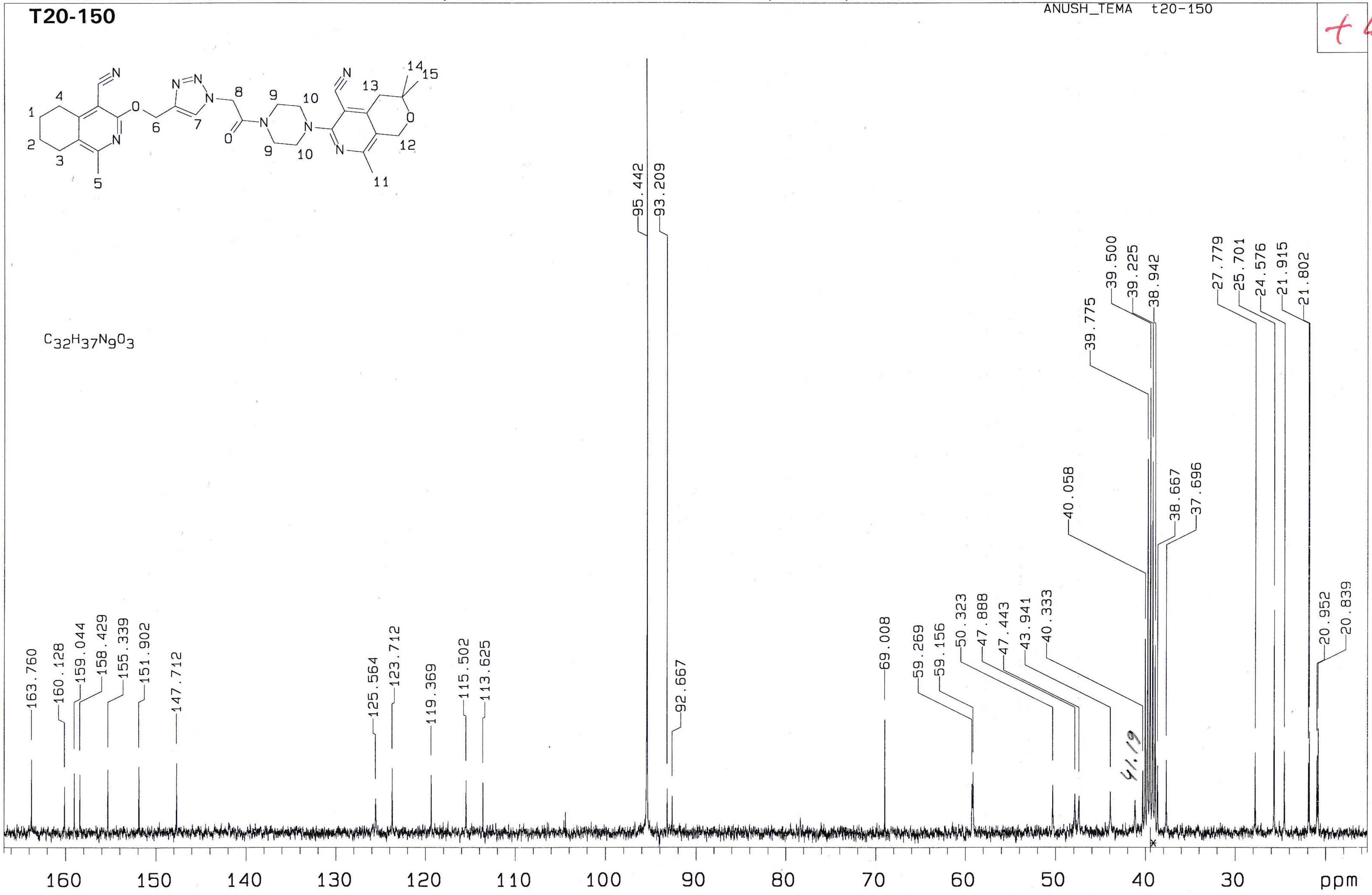

7e

Molecular Structure Research Centre, Yerevan, Armenia, Varian Mercury-300VX

H1 300.088 MHz, nt = 16, np = 32000, temp = 30.0 C, lb = -0.2, solvent = DMSO

Feb 23 2022

NOCI_22 t20-171

T20-171

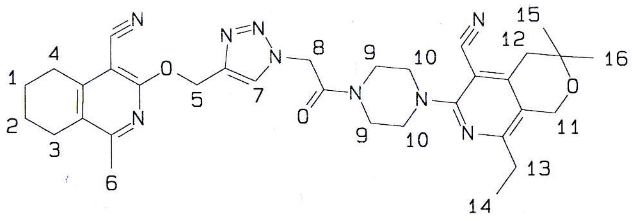

$C_{33}H_{39}N_9O_3$

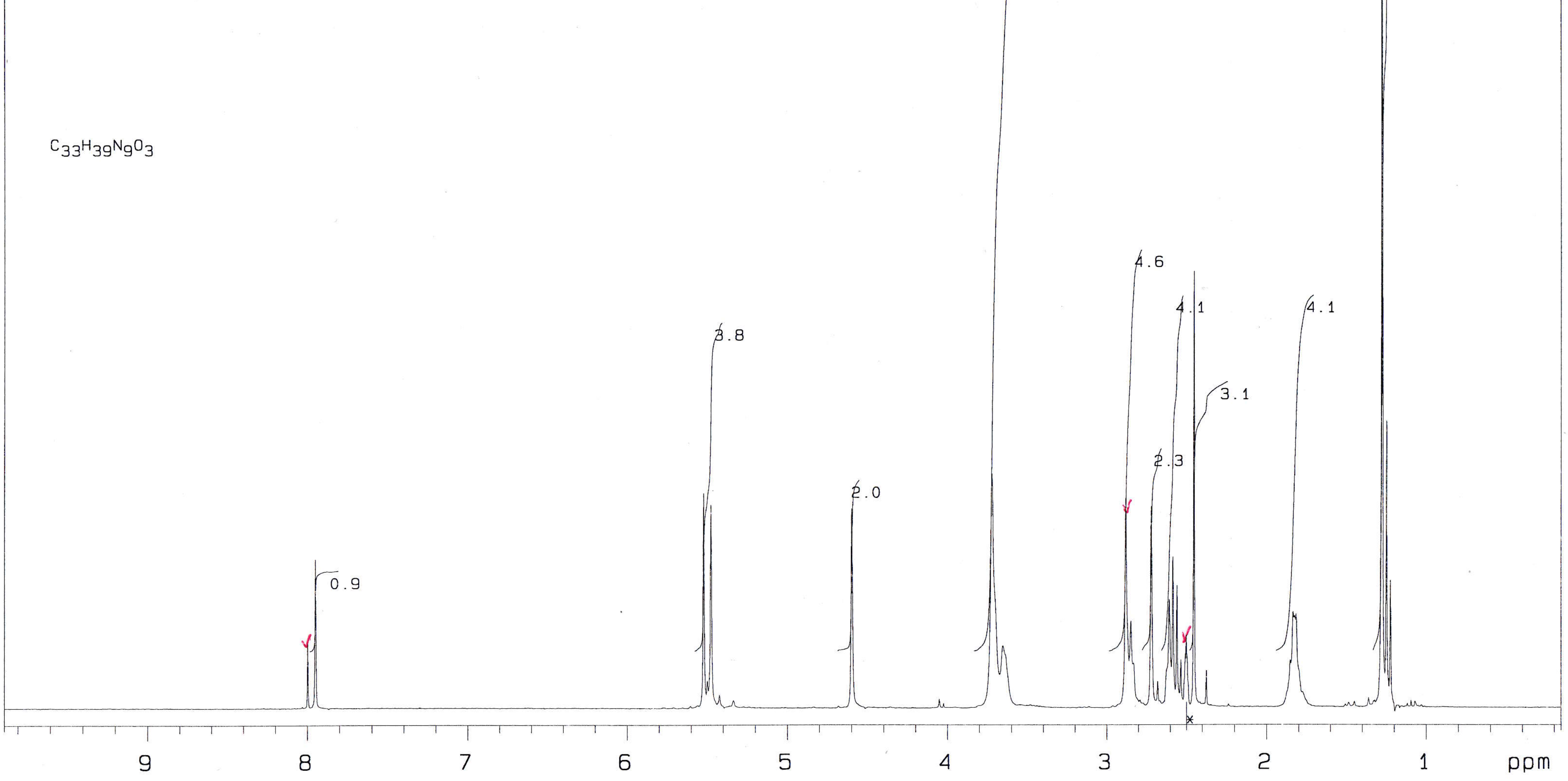

7e

Molecular Structure Research Centre, Yerevan, Armenia, Varian Mercury-300VX

C13 75.465 MHz, nt = 720, np = 19998, temp = 30.0 C, lb = 1.0, solvent = DMSO/CCl4 1/3

Feb 23 2022

T20-171

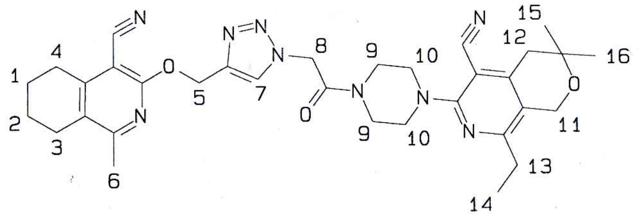

$C_{33}H_{39}N_9O_3$

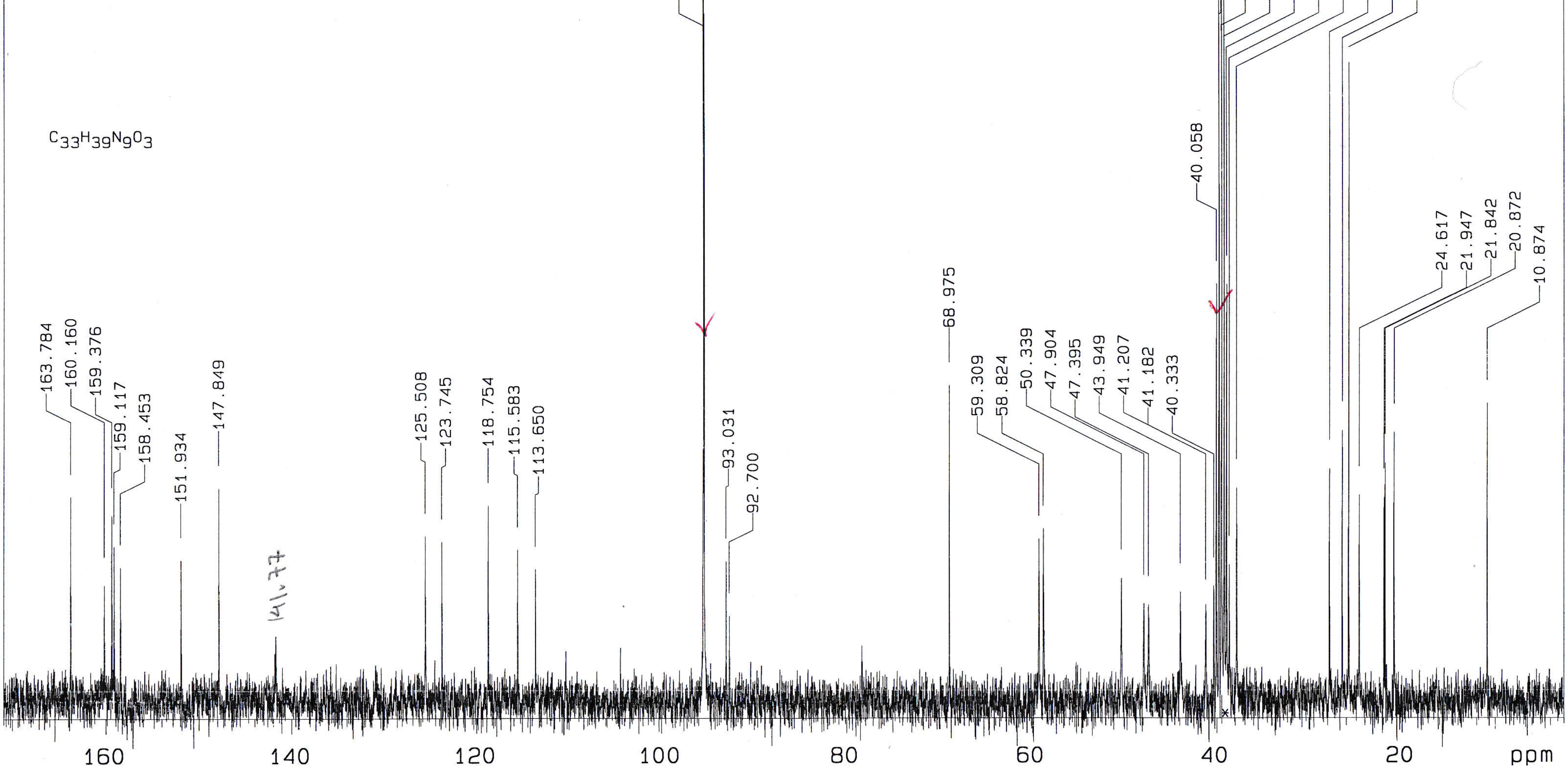

Molecular Structure Research Centre, Yerevan, Armenia, Varian Mercury-300VX

H1 300.088 MHz, nt = 16, np = 32000, temp = 30.0 C, lb = -0.2, solvent = DMSO

Feb 16 2022

T20-169

ANUSH_TEMA t20-169

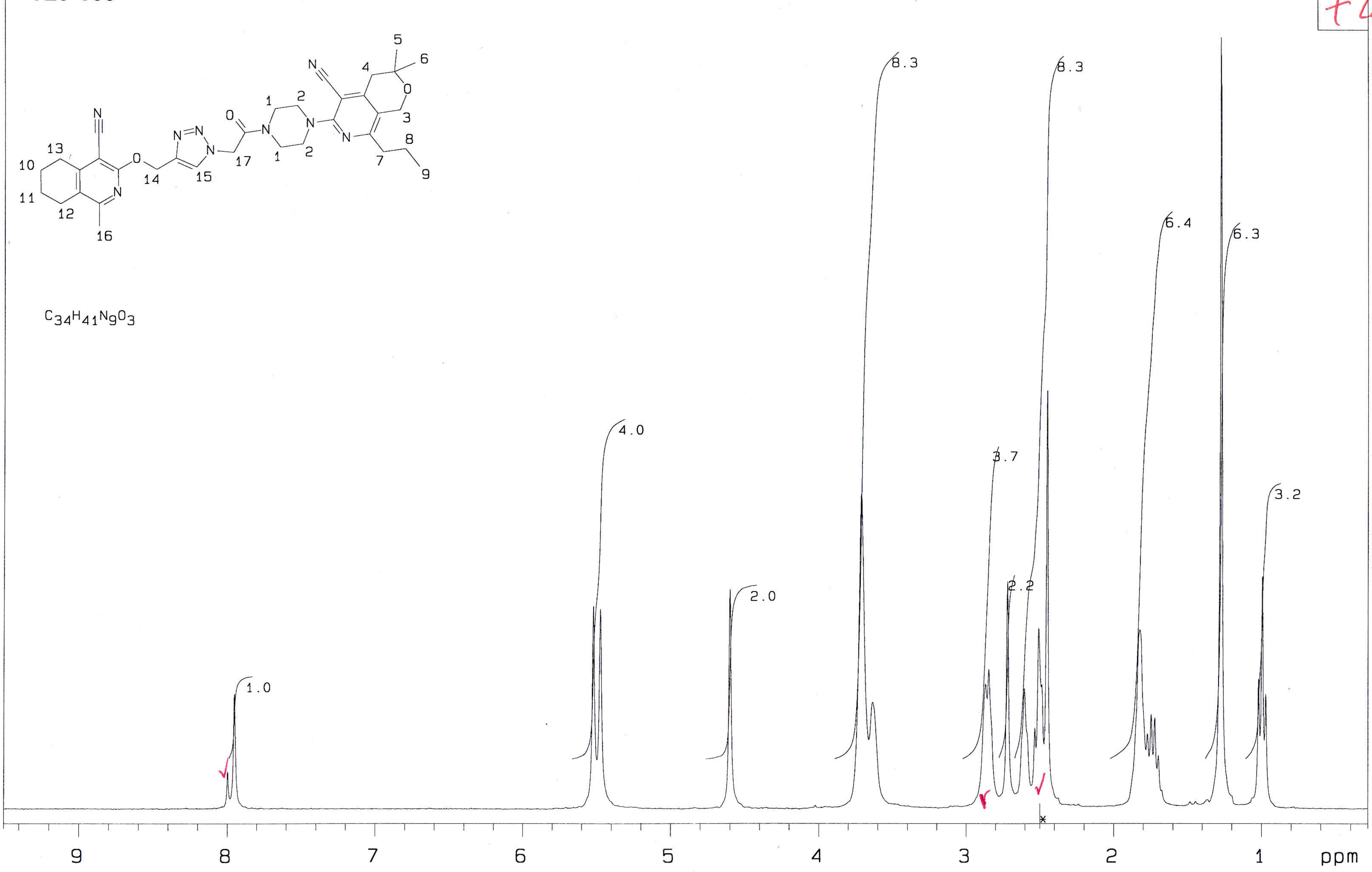

7f

Molecular Structure Research Centre, Yerevan, Armenia, Varian Mercury-300VX

C13 75.465 MHz, nt = 720, np = 19998, temp = 30.0 C, lb = 1.0, solvent = DMSO/CCl4 1/3

Feb 16 2022

ANUSH_TEMA t20-169

T20-169

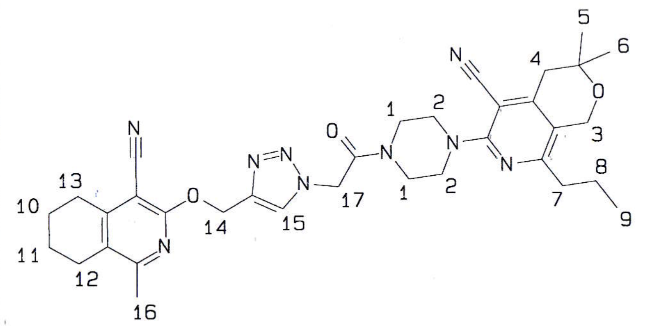

$C_{34}H_{41}N_9O_3$

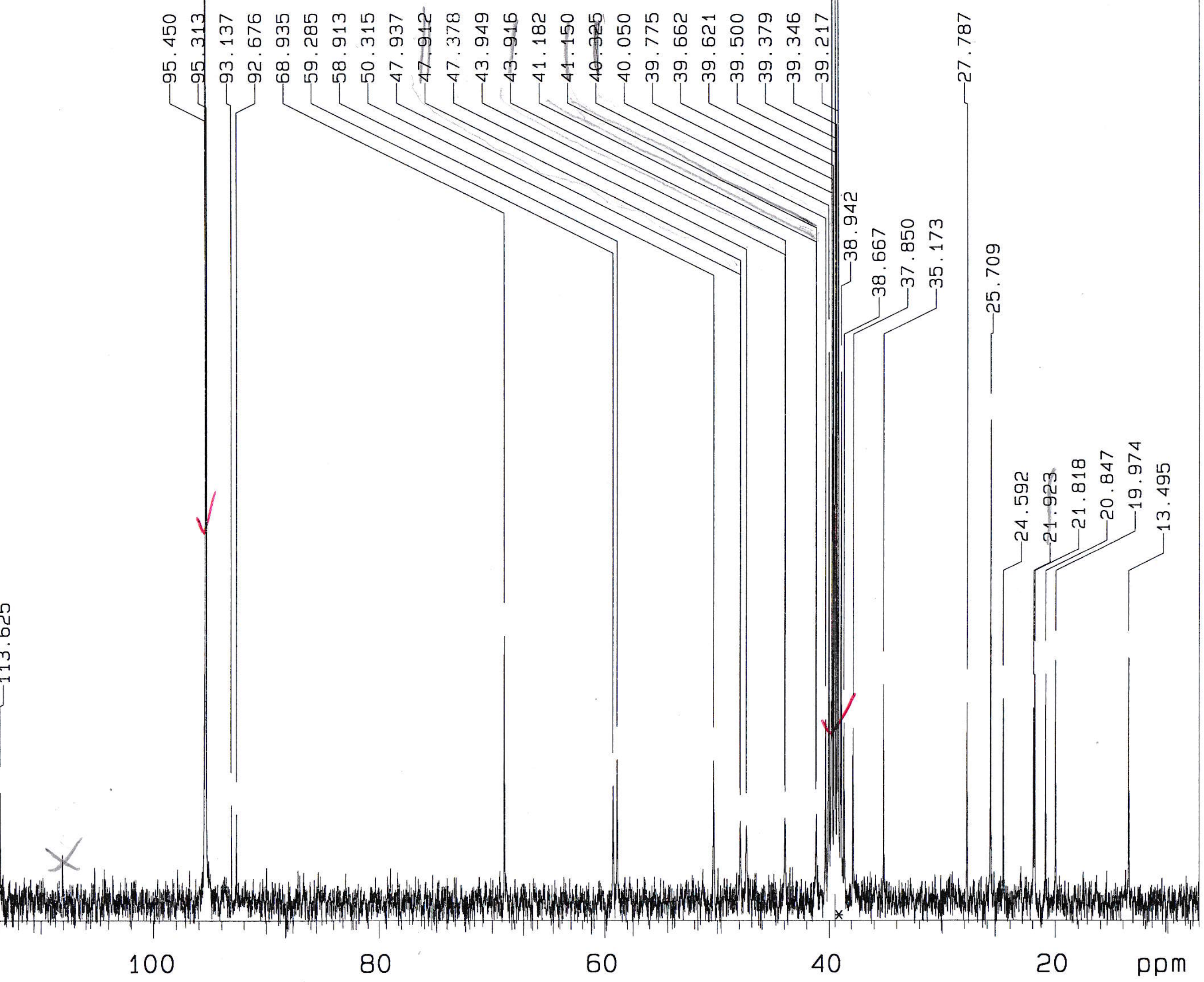

Molecular Structure Research Centre, Yerevan, Armenia, Varian Mercury-300VX

H1 300.088 MHz, nt = 16, np = 32000, temp = 30.0 C, lb = -0.2, solvent = DMSO/CCl4 1/3

May 13 2019

7g

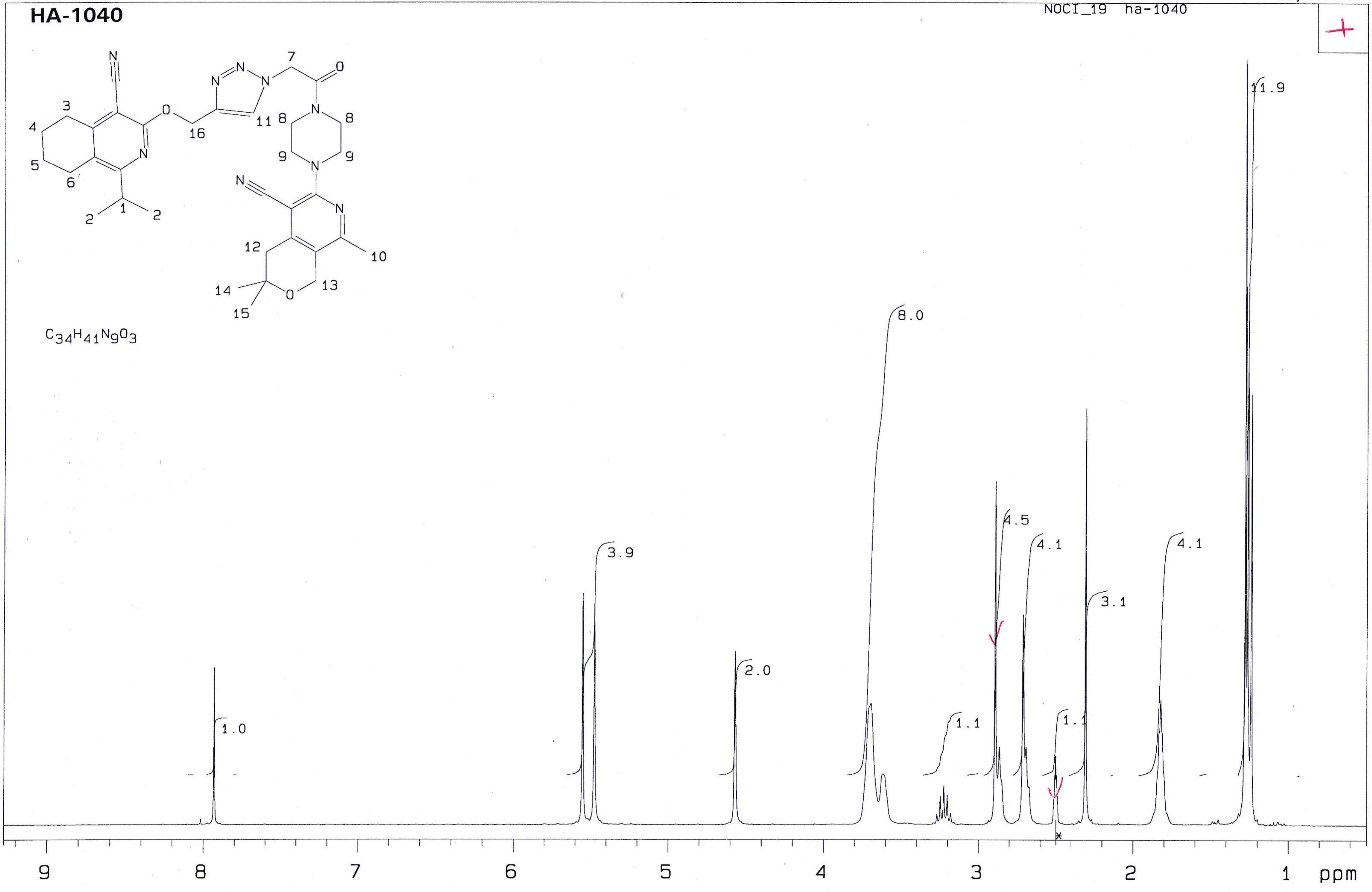

7g

Molecular Structure Research Centre, Yerevan, Armenia, Varian Mercury-300VX

C13 75.465 MHz, nt=512, np=19998, temp=30.0 C, lb=1.0, solvent=DMSO/CCl4 1/3

May 13 2019

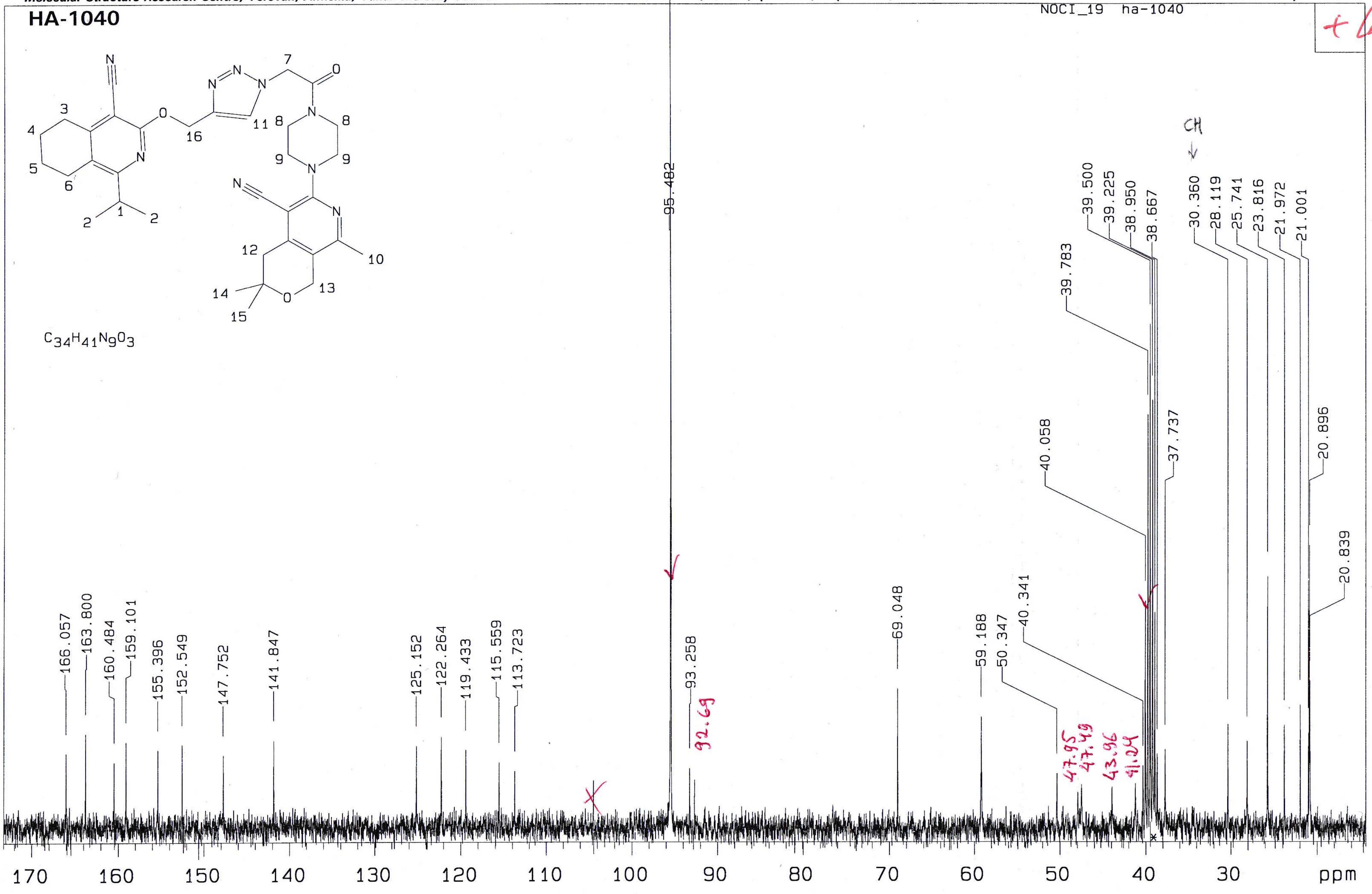

78

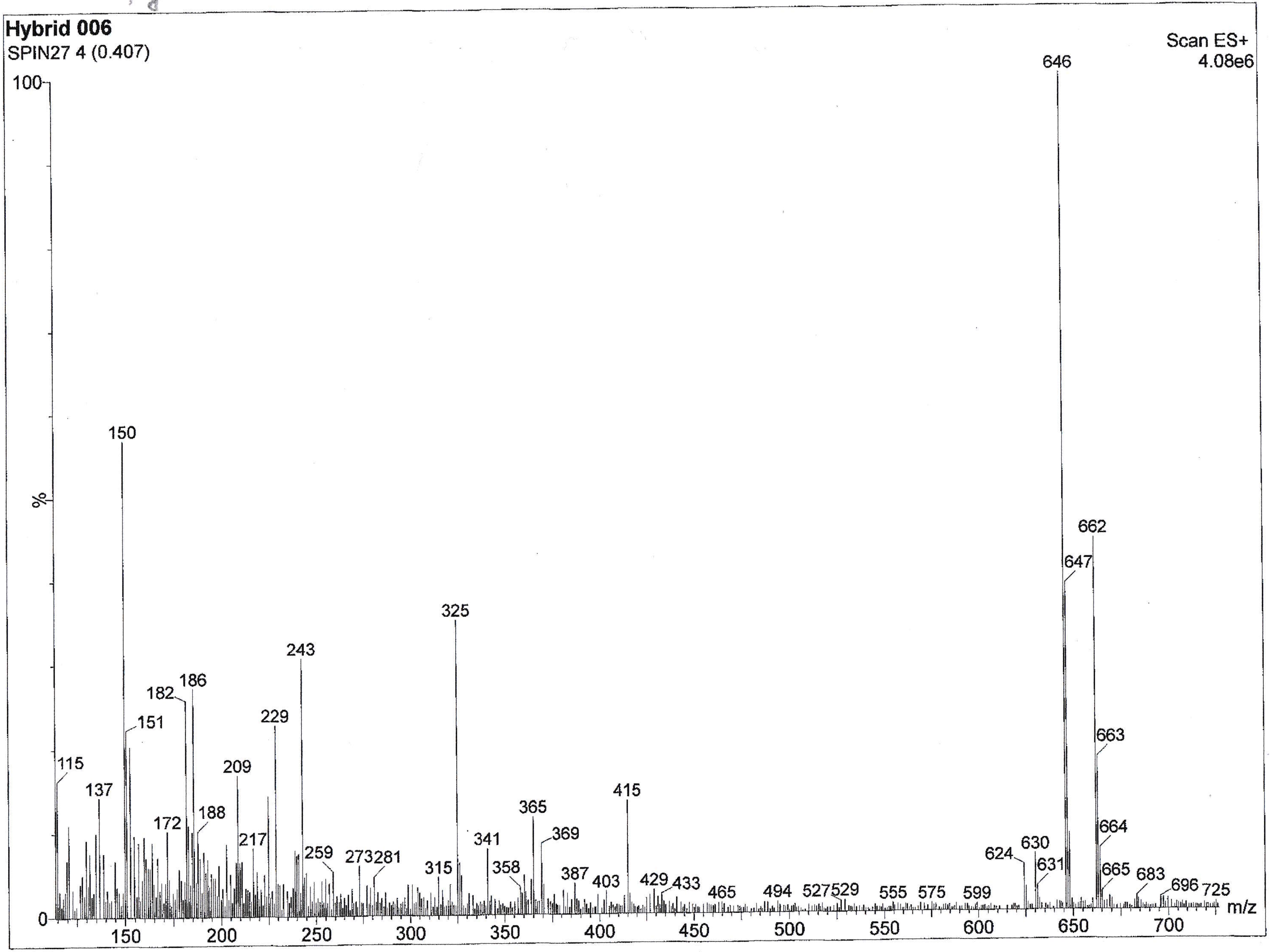

Hybrid 006
SPIN27 4 (0.407)
Scan ES+
4.08e6
100
%
0
646
662
647
663
664
665
683
696
725
624
630
631
599
575
555
527529
494
465
433
429
415
403
387
369
365
358
341
325
315
273281
259
243
229
217
209
188
186
182
172
151
150
137
115
150
200
250
300
350
400
450
500
550
600
650
700
m/z

7g

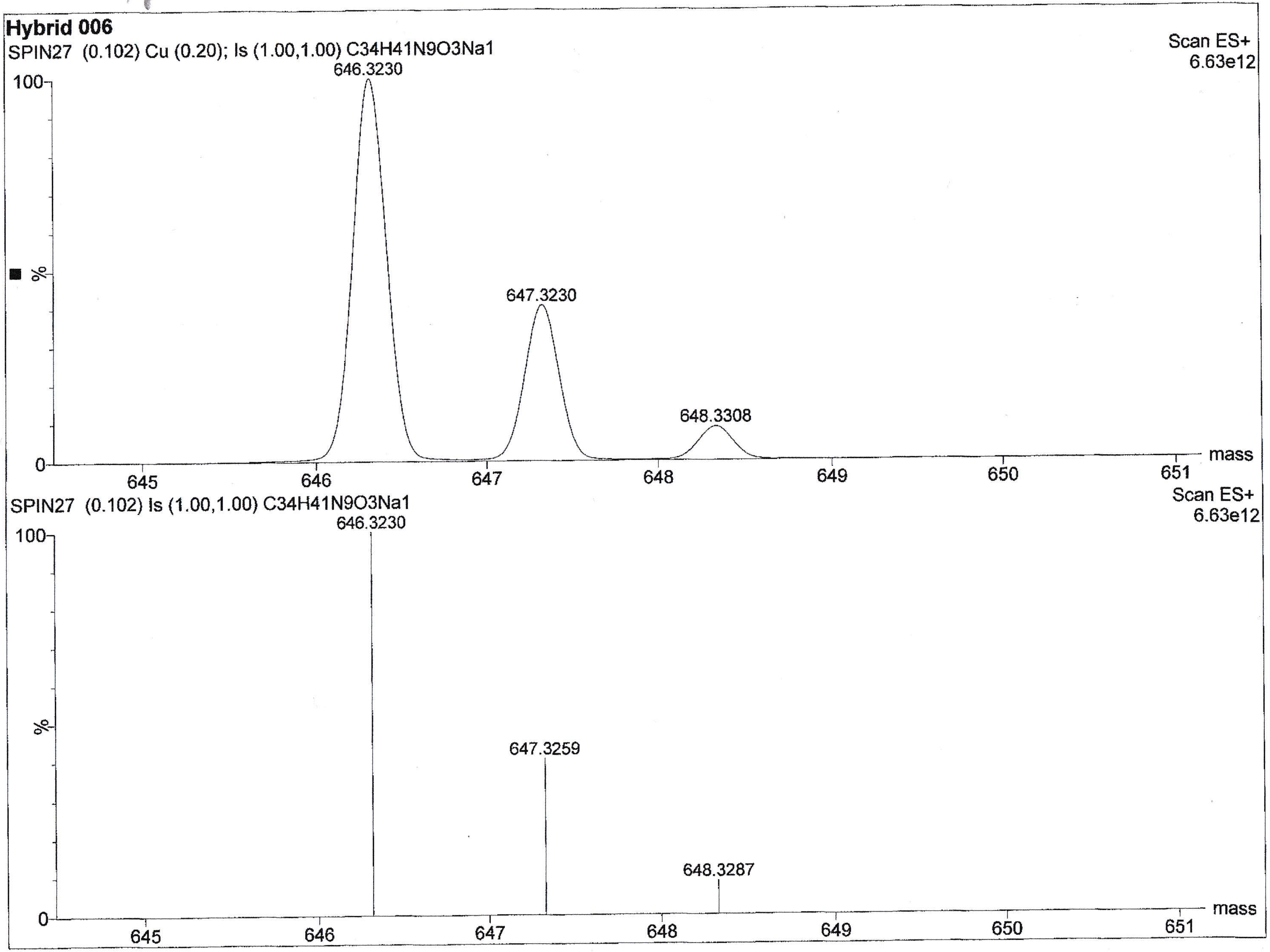

Hybrid 006
SPIN27 (0.102) Cu (0.20); Is (1.00,1.00) C34H41N9O3Na1
Scan ES+
6.63e12
100
%
0
646.3230
647.3230
648.3308
645
646
647
648
649
650
651
mass
SPIN27 (0.102) Is (1.00,1.00) C34H41N9O3Na1
Scan ES+
6.63e12
100
%
0
646.3230
647.3259
648.3287
645
646
647
648
649
650
651
mass

7i

Molecular Structure Research Centre, Yerevan, Armenia, Varian Mercury-300VX

H1 300.088 MHz, nt = 16, np = 32000, temp = 30.0 C, lb = -0.2, solvent = DMSO/CCl4 1/3

Mar 2 2022

NOCI_22 t20-170

+ [illegible]

T20-170

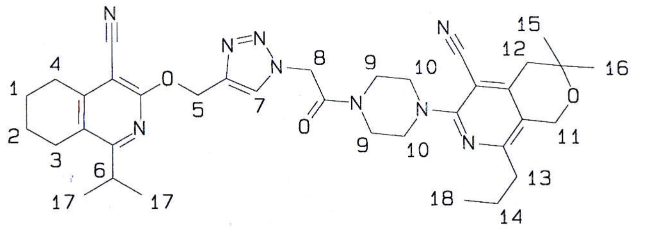

$C_{36}H_{45}N_9O_3$

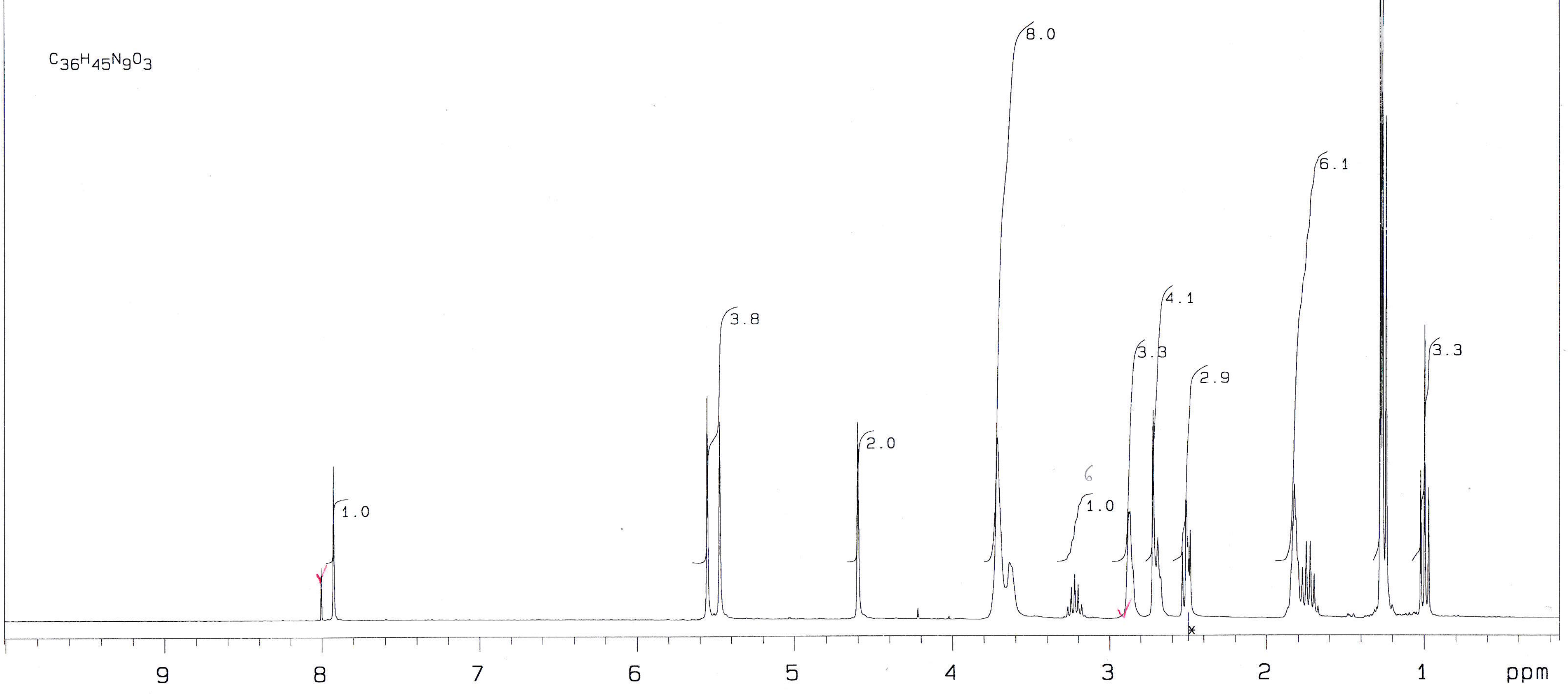

7i

Molecular Structure Research Centre, Yerevan, Armenia, Varian Mercury-300VX

C13 75.465 MHz, nt=784, np=19998, temp=30.0 C, lb=1.0, solvent=DMSO/CCl4 1/3

Mar 2 2022

NOCI_22 t20-170

T20-170

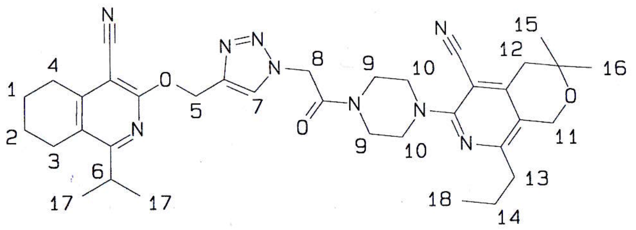

$C_{36}H_{45}N_9O_3$

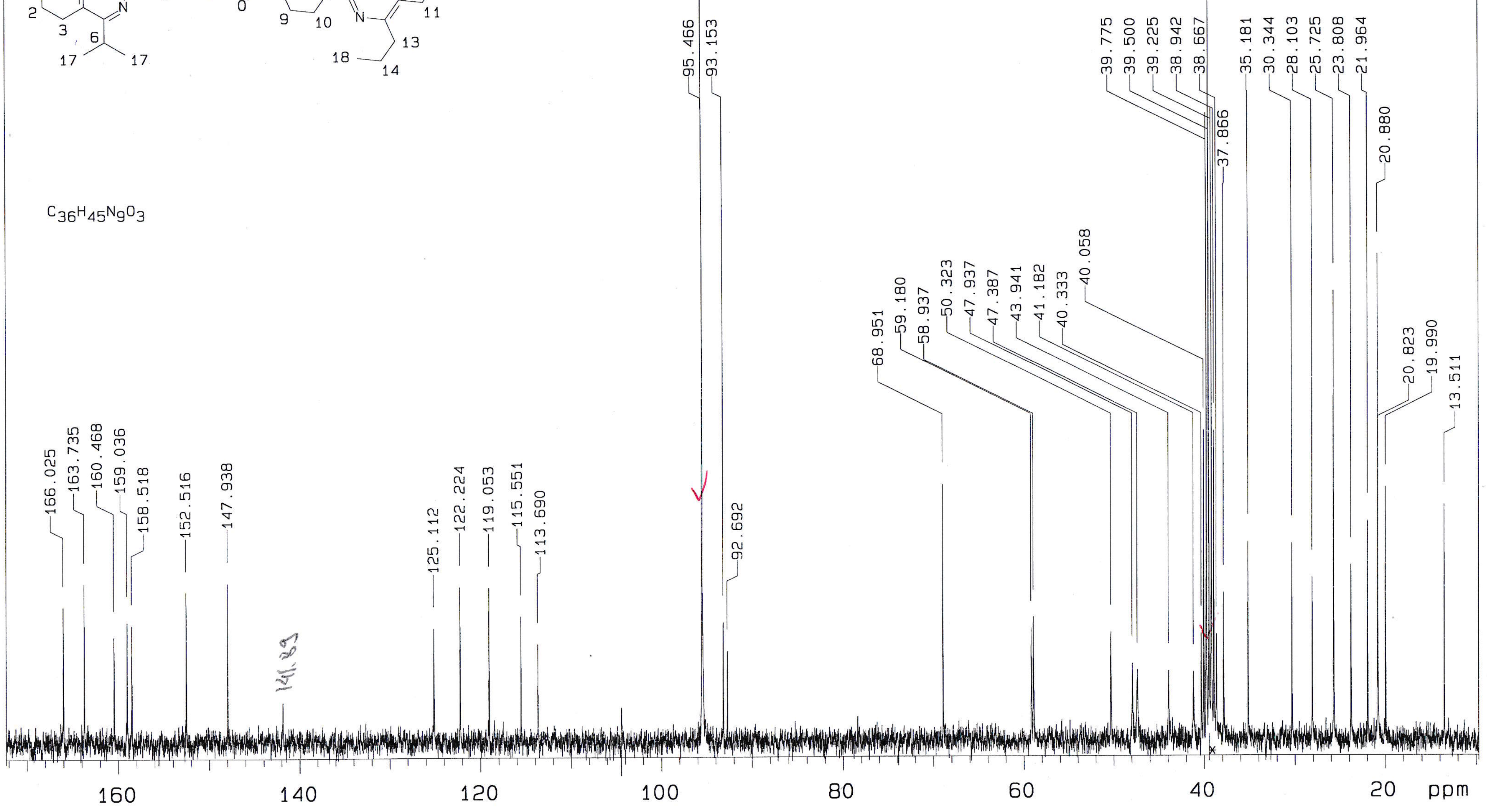

7j

Molecular Structure Research Centre, Yerevan, Armenia, Varian Mercury-300VX

H1 300.088 MHz, nt = 16, np = 32000, temp = 30.0 C, lb = -0.2, solvent = DMSO/CCl4 1/3

Apr 17 2019

SAMV_19 ha-1033

HA-1033

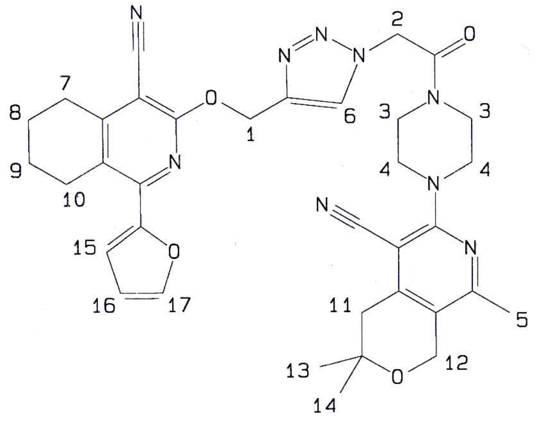

$C_{35}H_{37}N_9O_4$

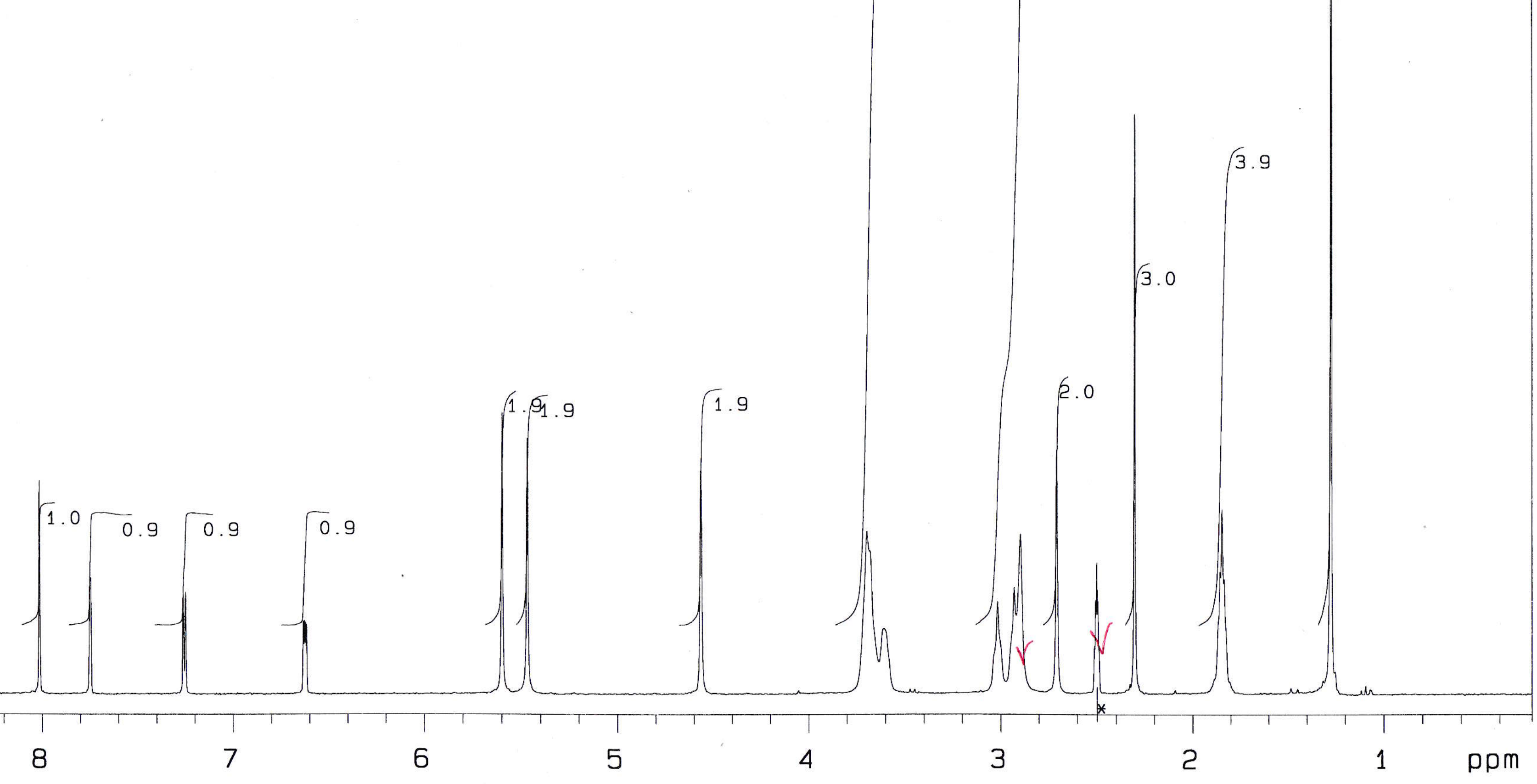

7i

Molecular Structure Research Centre, Yerevan, Armenia, Varian Mercury-300VX

C13 75.465 MHz, nt = 528, np = 19998, temp = 30.0 C, lb = 1.0, solvent = DMSO/CCl4 1/3

Apr 17 2019

HA-1033

SAMV_19 ha-1033

$C_{35}H_{37}N_9O_4$

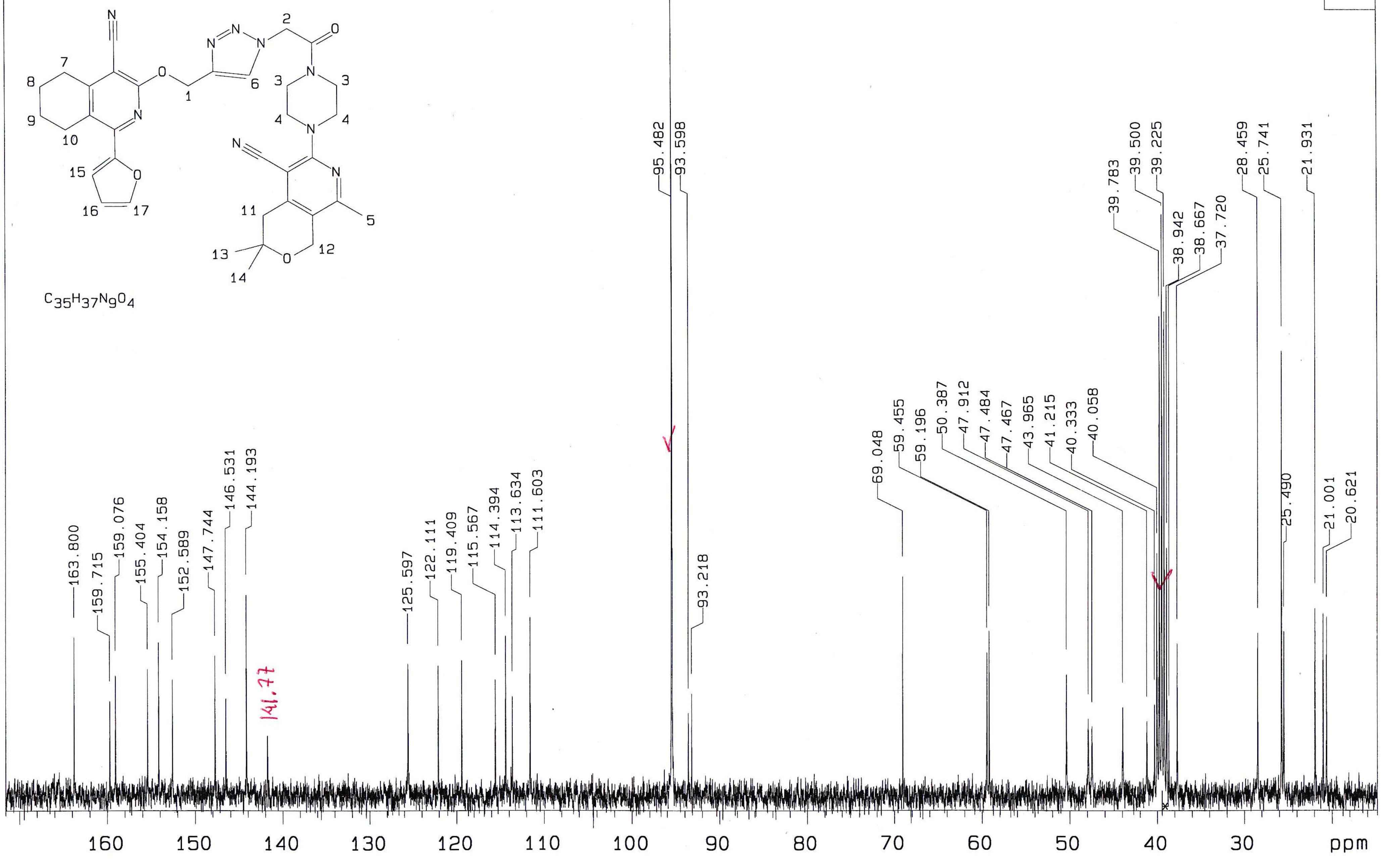

7j

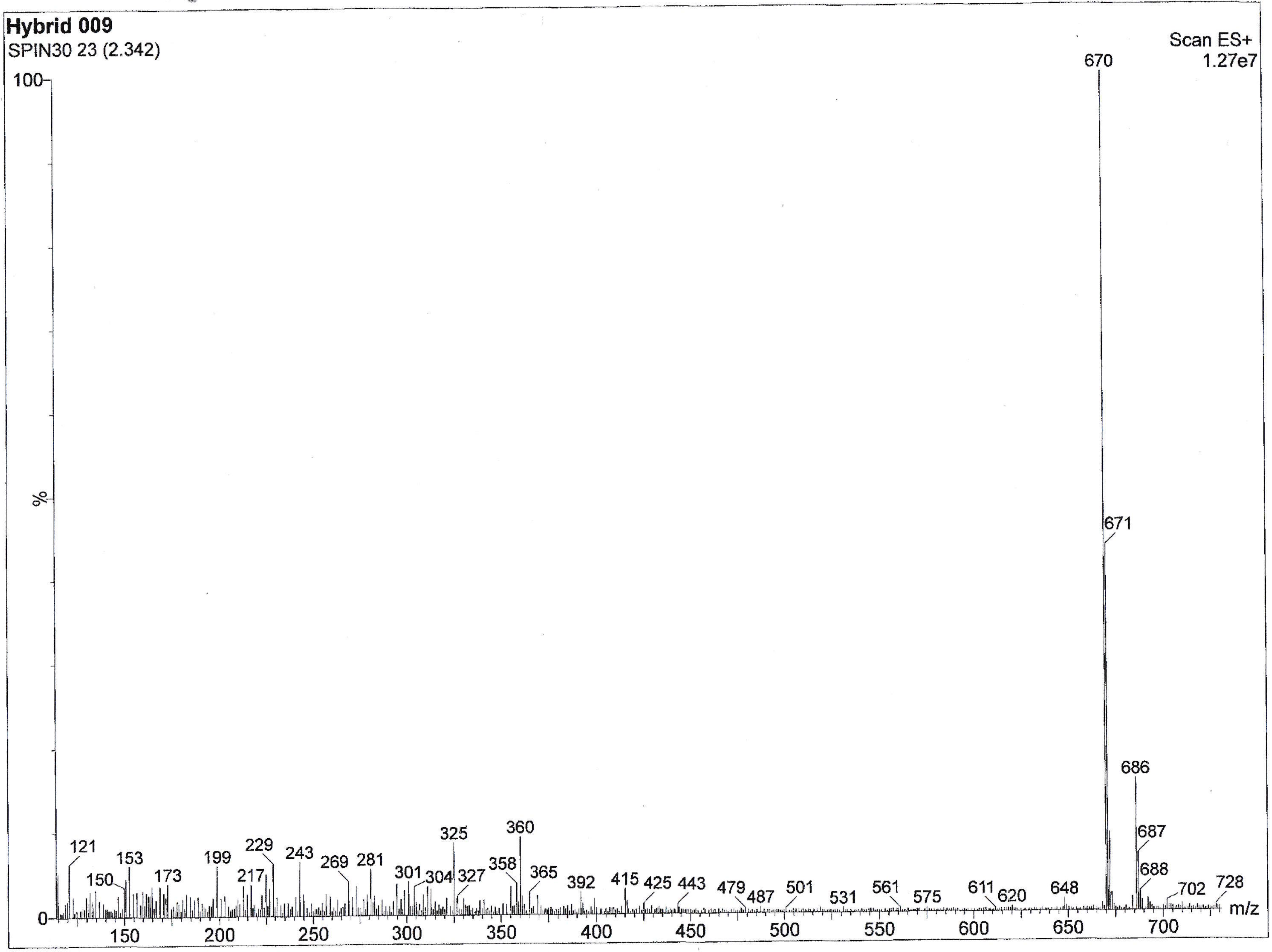

Hybrid 009
SPIN30 23 (2.342)
Scan ES+
1.27e7
100
%
0
670
671
686
687
688
702
728
121
150
153
173
199
217
229
243
269
281
301
304
325
327
358
360
365
392
415
425
443
479
487
501
531
561
575
611
620
648
150
200
250
300
350
400
450
500
550
600
650
700
m/z

7j

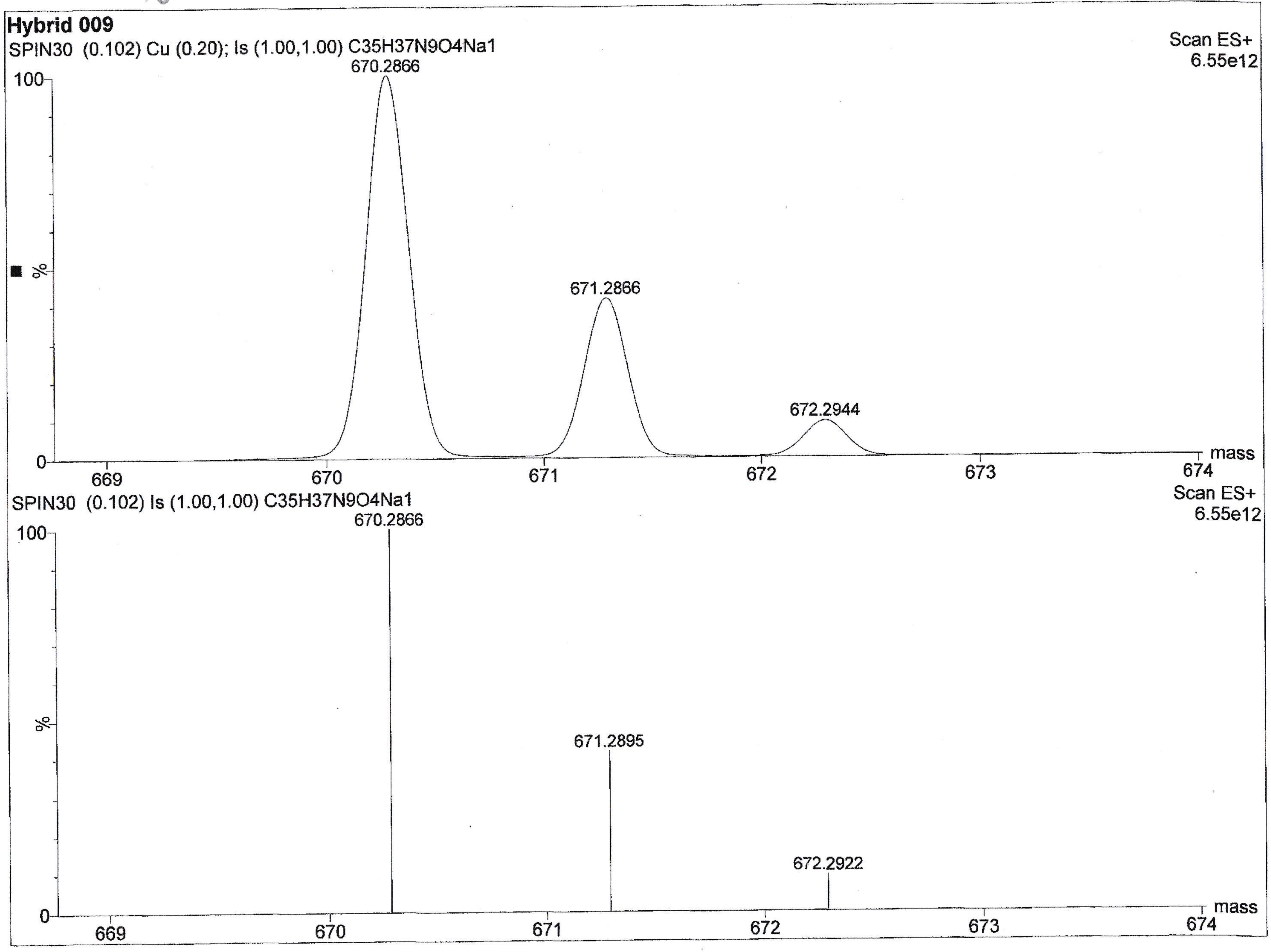
Hybrid 009
SPIN30 (0.102) Cu (0.20); Is (1.00,1.00) C35H37N9O4Na1
Scan ES+
6.55e12
100
%
0
670.2866
671.2866
672.2944
669
670
671
672
673
674
mass
SPIN30 (0.102) Is (1.00,1.00) C35H37N9O4Na1
Scan ES+
6.55e12
100
%
0
670.2866
671.2895
672.2922
669
670
671
672
673
674
mass

7k

Molecular Structure Research Centre, Yerevan, Armenia, Varian Mercury-300VX

H1 300.088 MHz, nt = 16, np = 32000, temp = 30.0 C, lb = -0.2, solvent = DMSO/CCl4 1/3

Feb 11 2022

ANUSH_TEMA t20-166

T20-166

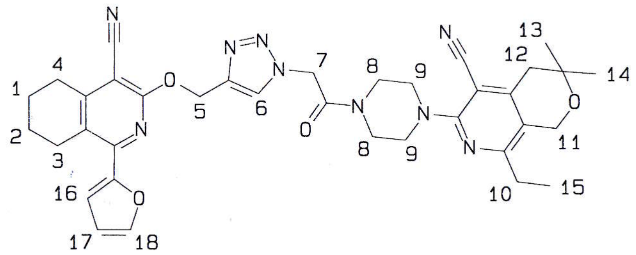

$C_{36}H_{39}N_9O_4$

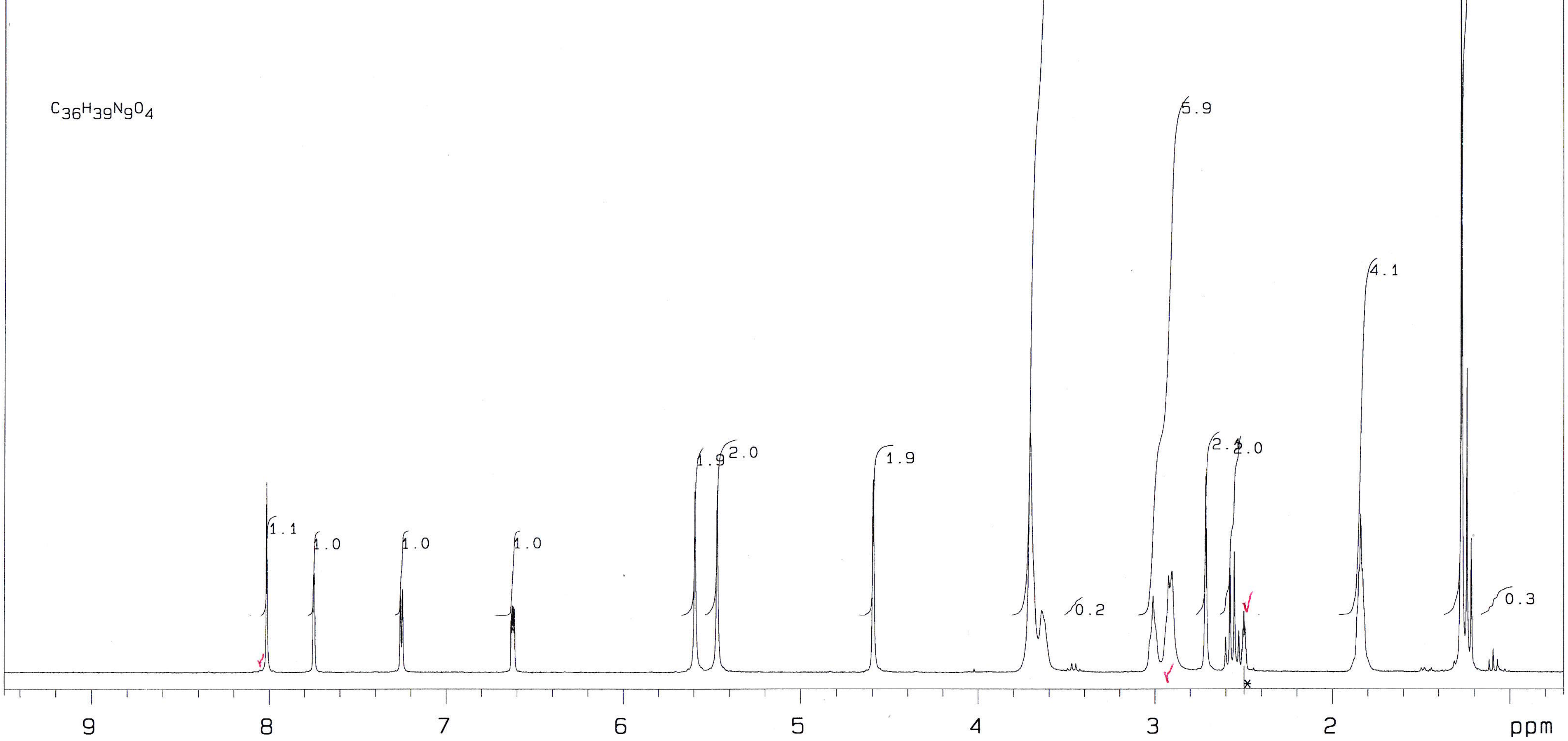

+

7K

Molecular Structure Research Centre, Yerevan, Armenia, Varian Mercury-300VX

C13 75.465 MHz, nt = 560, np = 19998, temp = 30.0 C, lb = 1.0, solvent = DMSO/CCl4 1/3

Feb 11 2022

T20-166

ANUSH_TEMA t20-166

$C_{36}H_{39}N_9O_4$

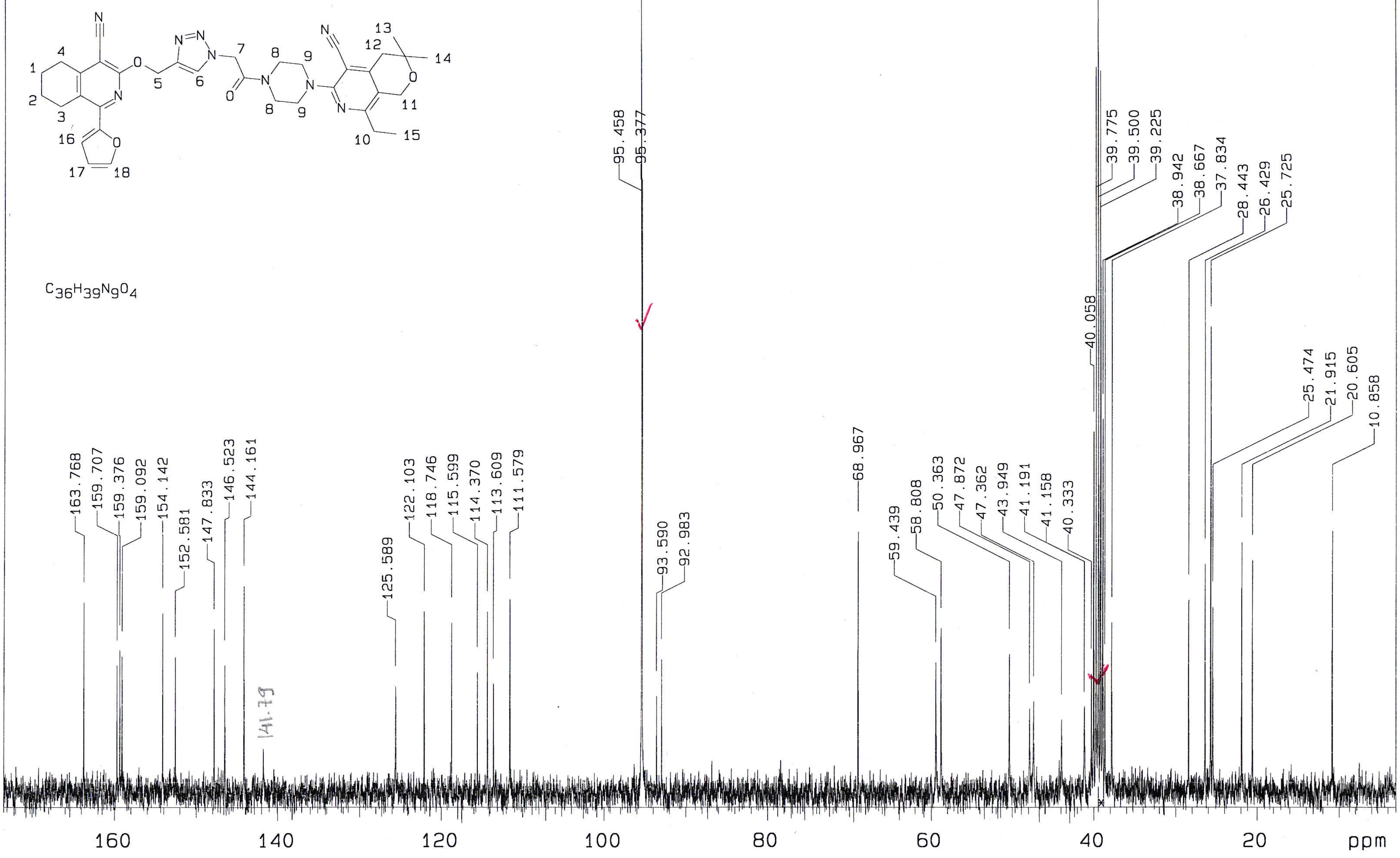

7e

Molecular Structure Research Centre, Yerevan, Armenia, Varian Mercury-300VX

H1 300.088 MHz, nt = 16, np = 32000, temp = 30.0 C, lb = -0.2, solvent = DMSO/CCl4 1/3

Mar 21 2019

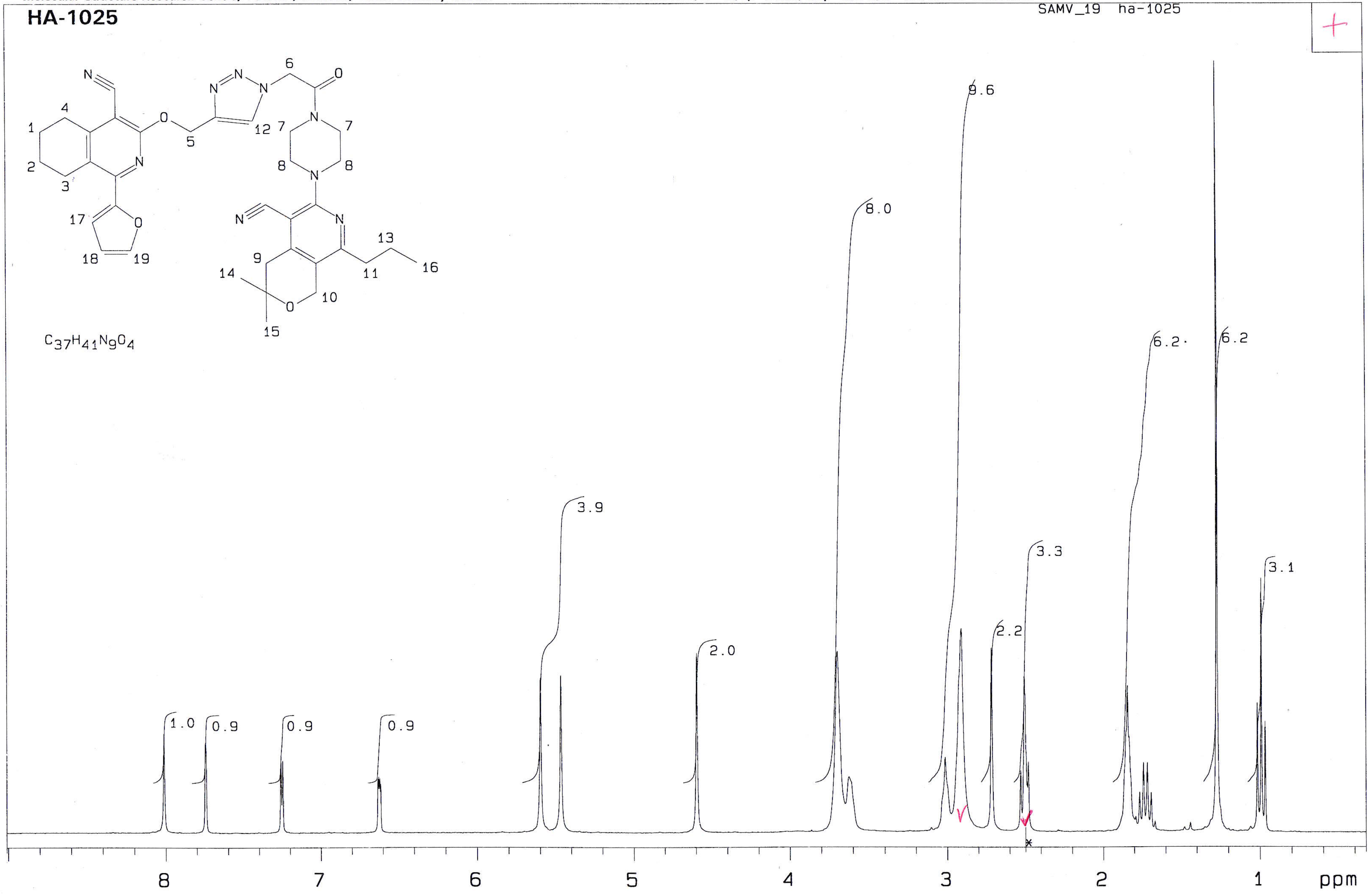

7l

Molecular Structure Research Centre, Yerevan, Armenia, Varian Mercury-300VX

C13 75.465 MHz, nt = 1296, np = 19998, temp = 30.0 C, lb = 1.0, solvent = DMSO/CCl4 1/3

Mar 21 2019

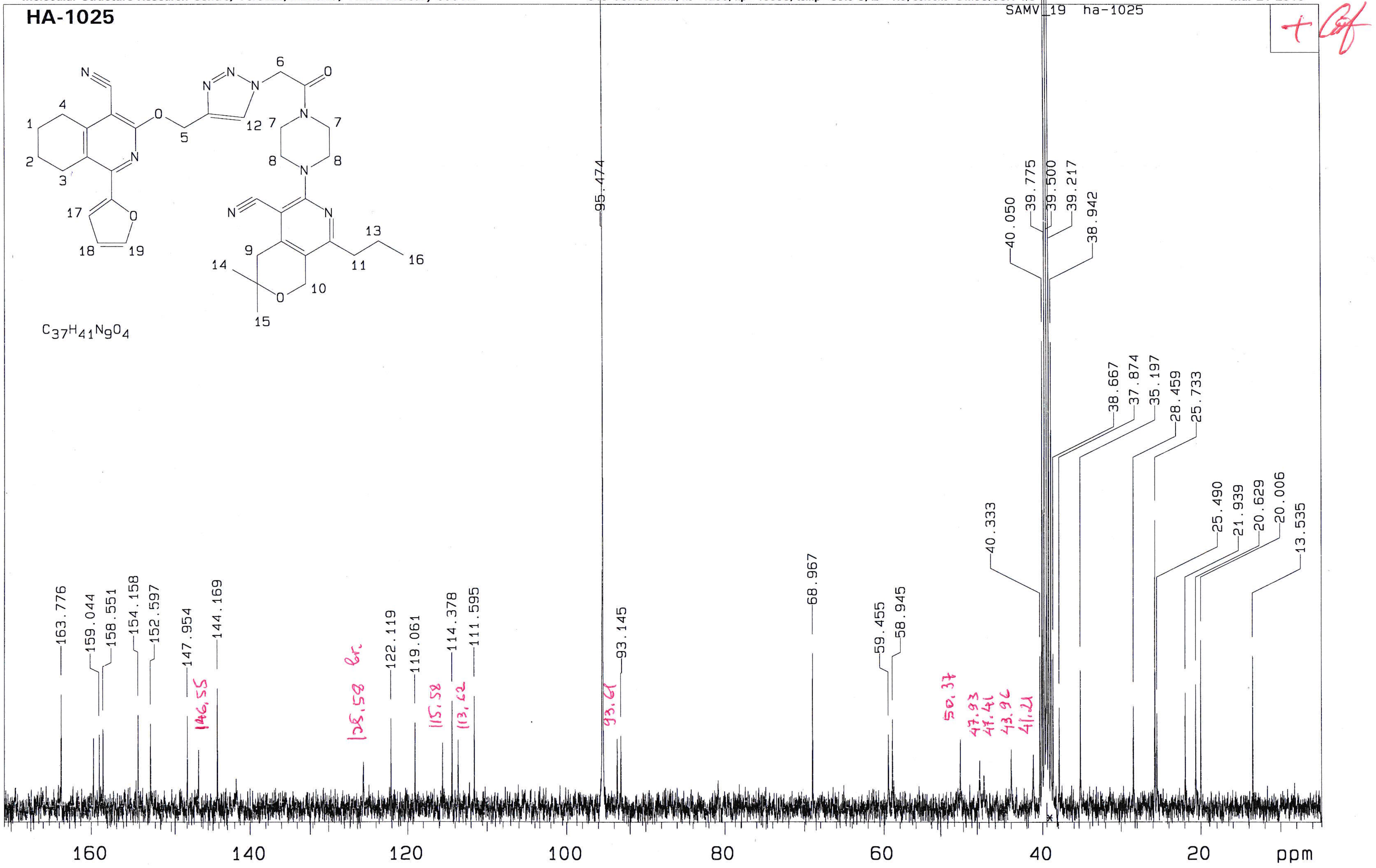

7e

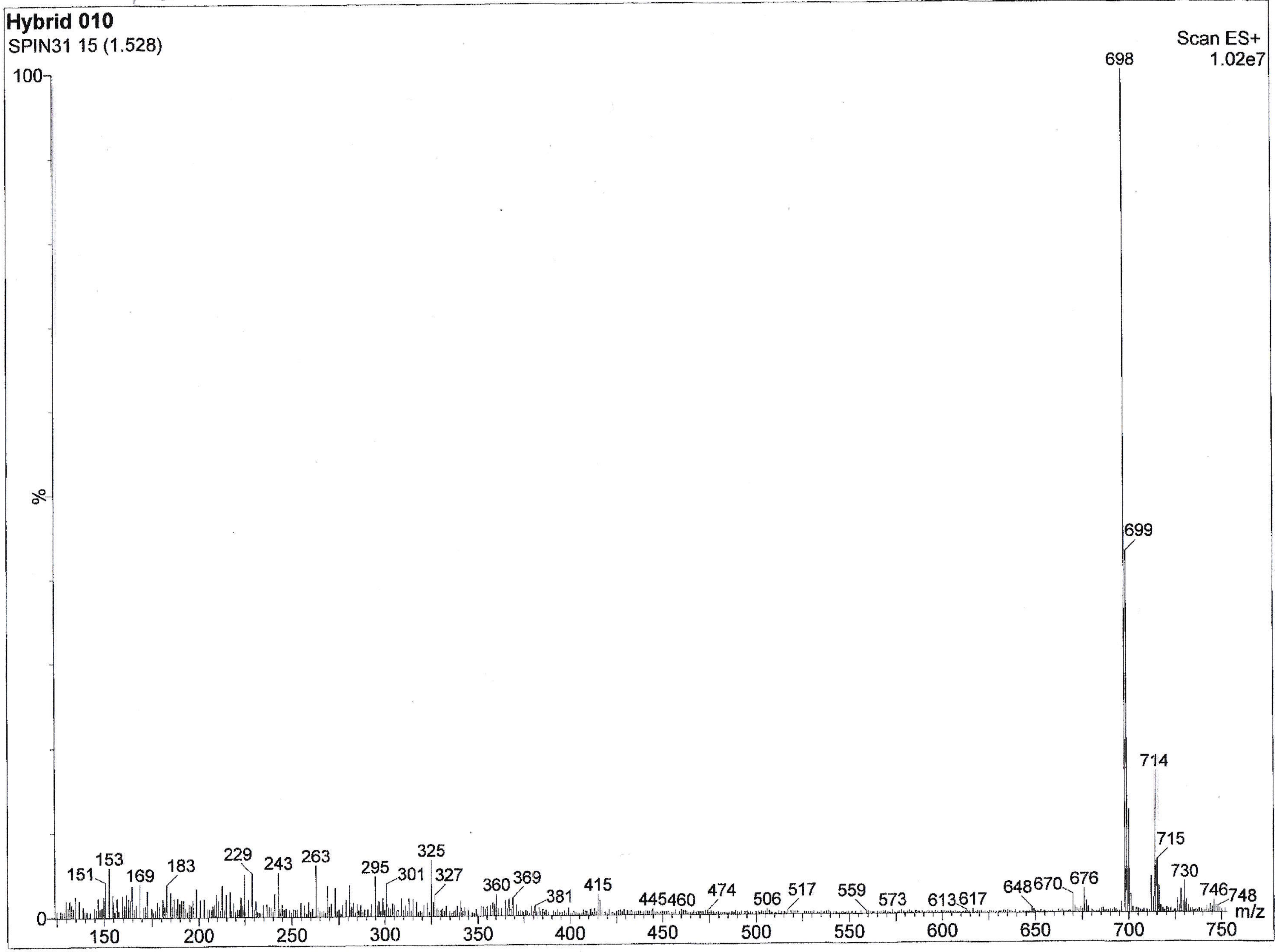

Hybrid 010
SPIN31 15 (1.528)
Scan ES+
1.02e7
100
%
0
698
699
714
715
730
746
748
151
153
169
183
229
243
263
295
301
325
327
360
369
381
415
445460
474
506
517
559
573
613617
648670
676
150
200
250
300
350
400
450
500
550
600
650
700
750
m/z

7e

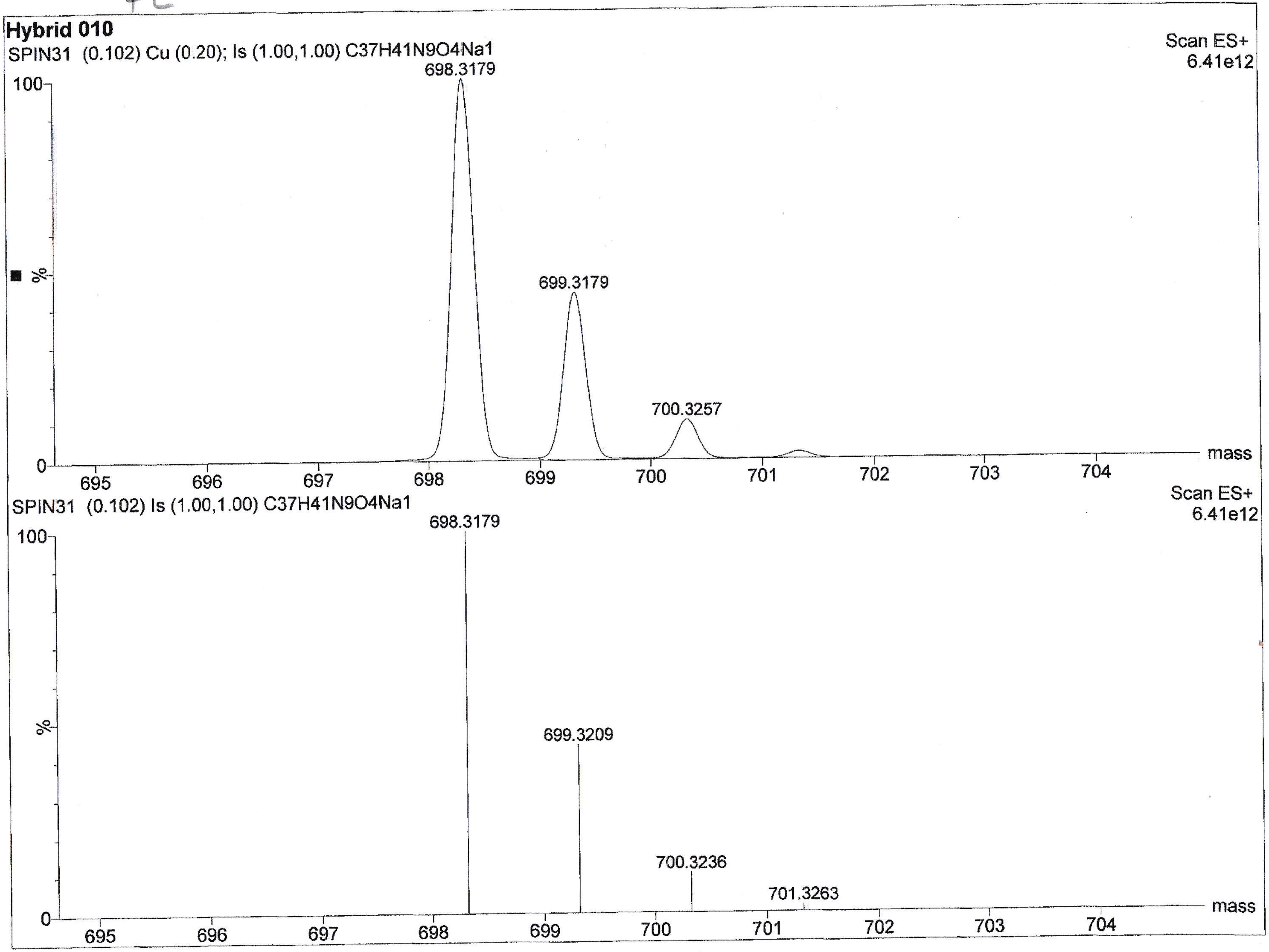

Hybrid 010
SPIN31 (0.102) Cu (0.20); Is (1.00,1.00) C37H41N9O4Na1
Scan ES+
6.41e12
100
%
0
698.3179
699.3179
700.3257
695
696
697
698
699
700
701
702
703
704
mass
SPIN31 (0.102) Is (1.00,1.00) C37H41N9O4Na1
Scan ES+
6.41e12
100
%
0
698.3179
699.3209
700.3236
701.3263
695
696
697
698
699
700
701
702
703
704
mass

7m

Molecular Structure Research Centre, Yerevan, Armenia, Varian Mercury-300VX

H1 300.088 MHz, nt=16, np=32000, temp=30.0 C, lb=-0.2, solvent=DMSO/CCl4 1/3

Apr 12 2019

SAMV_19 ha-1032

HA-1032

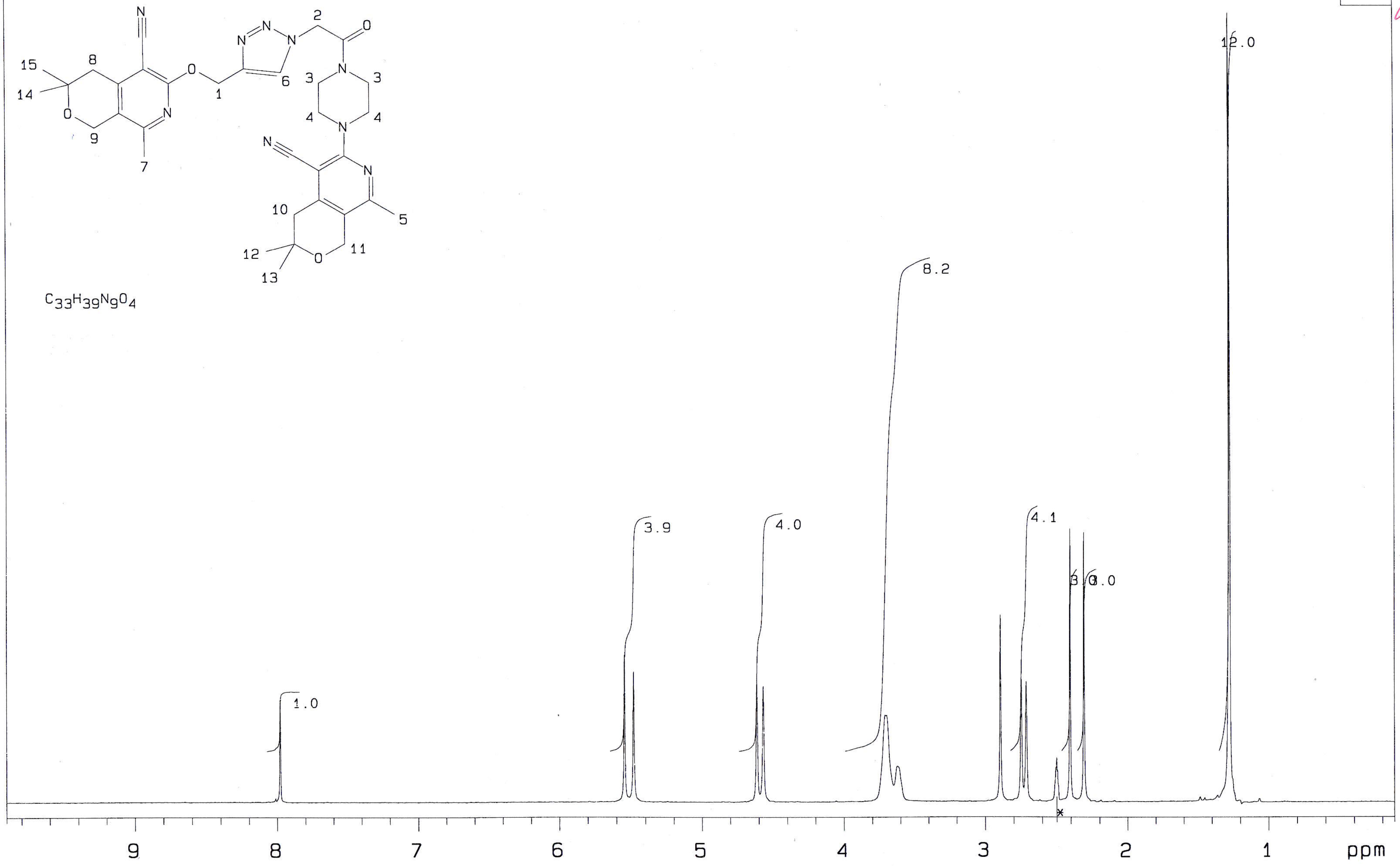

$C_{33}H_{39}N_9O_4$

7m

Molecular Structure Research Centre, Yerevan, Armenia, Varian Mercury-300VX

C13 75.465 MHz, nt=656, np=19998, temp=30.0 C, lb=1.0, solvent=DMSO/CCl4 1/3

Apr 12 2019

HA-1032

SAMV_19 ha-1032

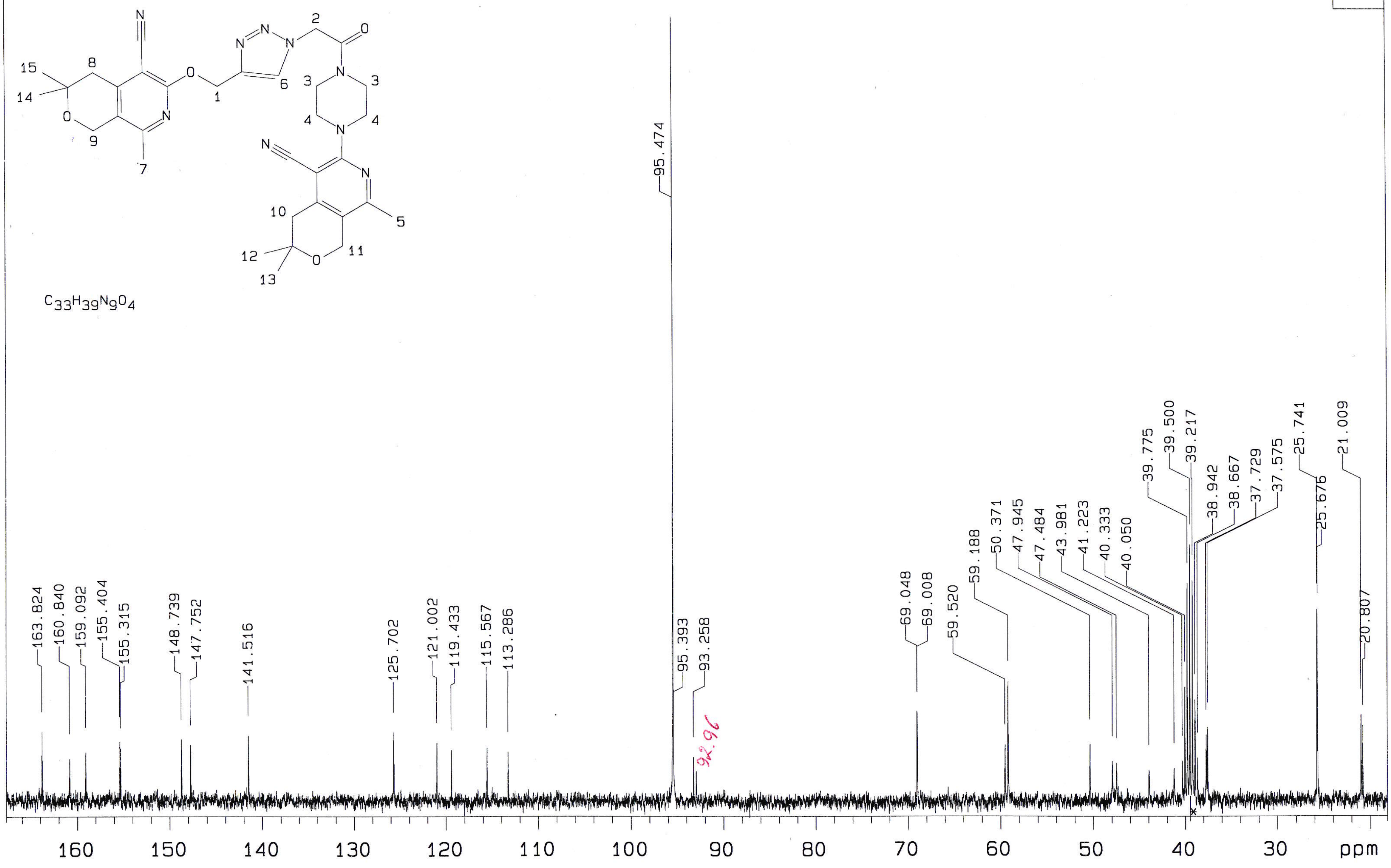

$C_{33}H_{39}N_9O_4$

7 m

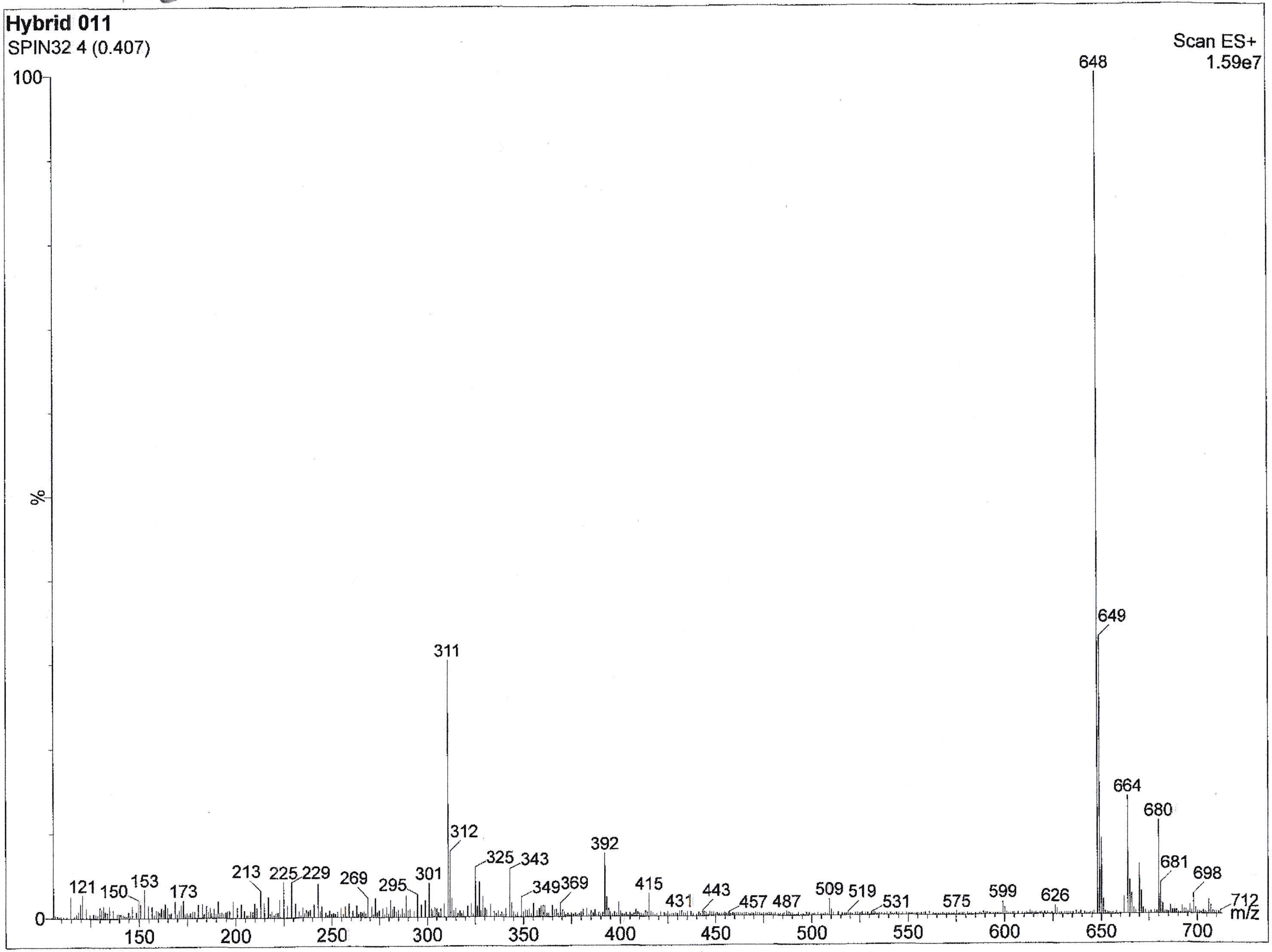

Hybrid 011
SPIN32 4 (0.407)
Scan ES+
1.59e7
100
%
0
648
649
311
664
680
681
698
712
312
392
325
343
349
369
415
431
443
457
487
509
519
531
575
599
626
121
150
153
173
213
225
229
269
295
301
150
200
250
300
350
400
450
500
550
600
650
700
m/z

7 m

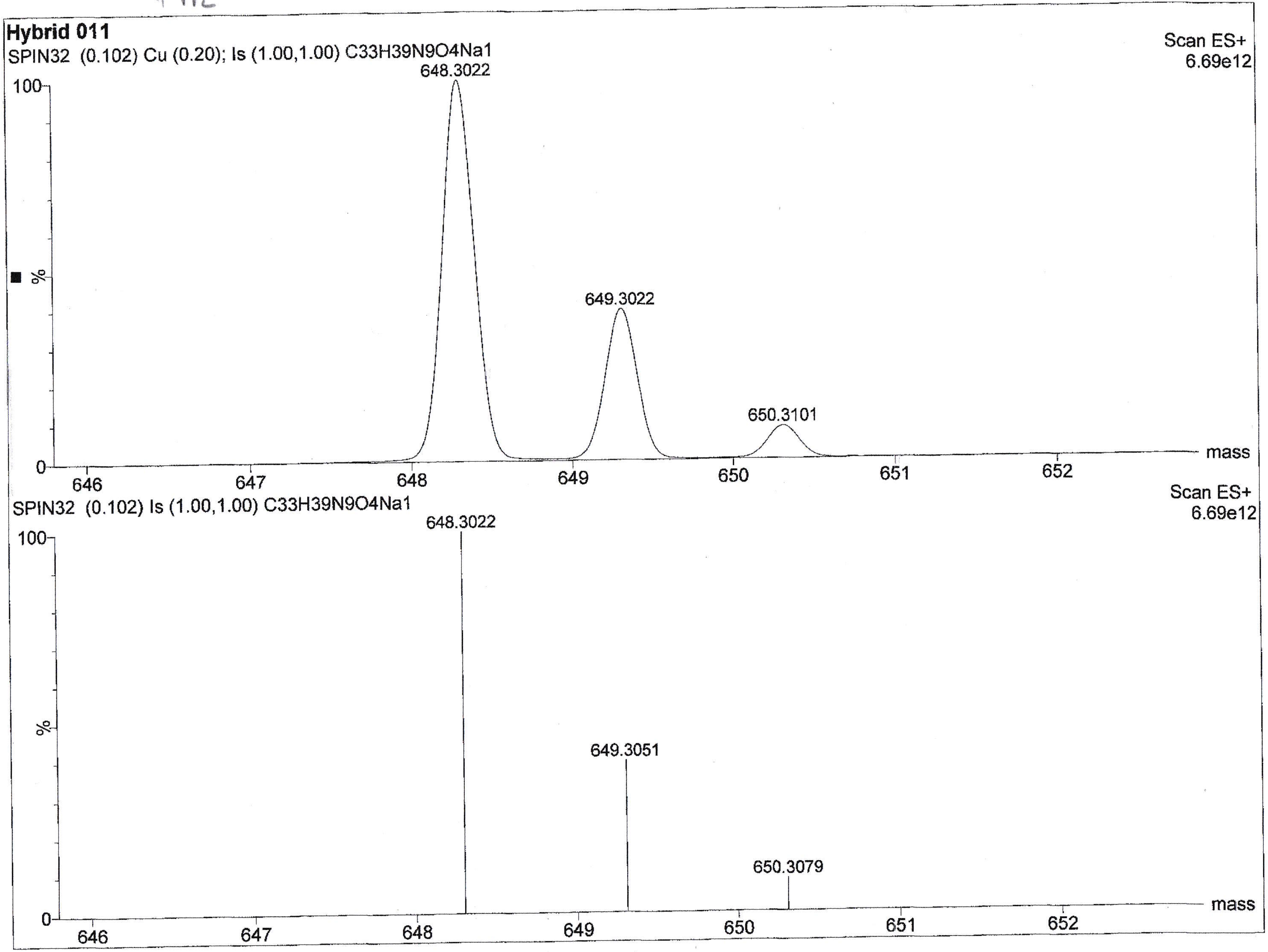
Hybrid 011
SPIN32 (0.102) Cu (0.20); Is (1.00,1.00) C33H39N9O4Na1
Scan ES+
6.69e12
648.3022
649.3022
650.3101
100
%
0
646
647
648
649
650
651
652
mass
SPIN32 (0.102) Is (1.00,1.00) C33H39N9O4Na1
Scan ES+
6.69e12
648.3022
649.3051
650.3079
100
%
0
646
647
648
649
650
651
652
mass

7n

Molecular Structure Research Centre, Yerevan, Armenia, Varian Mercury-300VX

H1 300.088 MHz, nt=16, np=32000, temp=30.0 C, lb=-0.2, solvent=DMSO/CCl4 1/3

Mar 11 2022

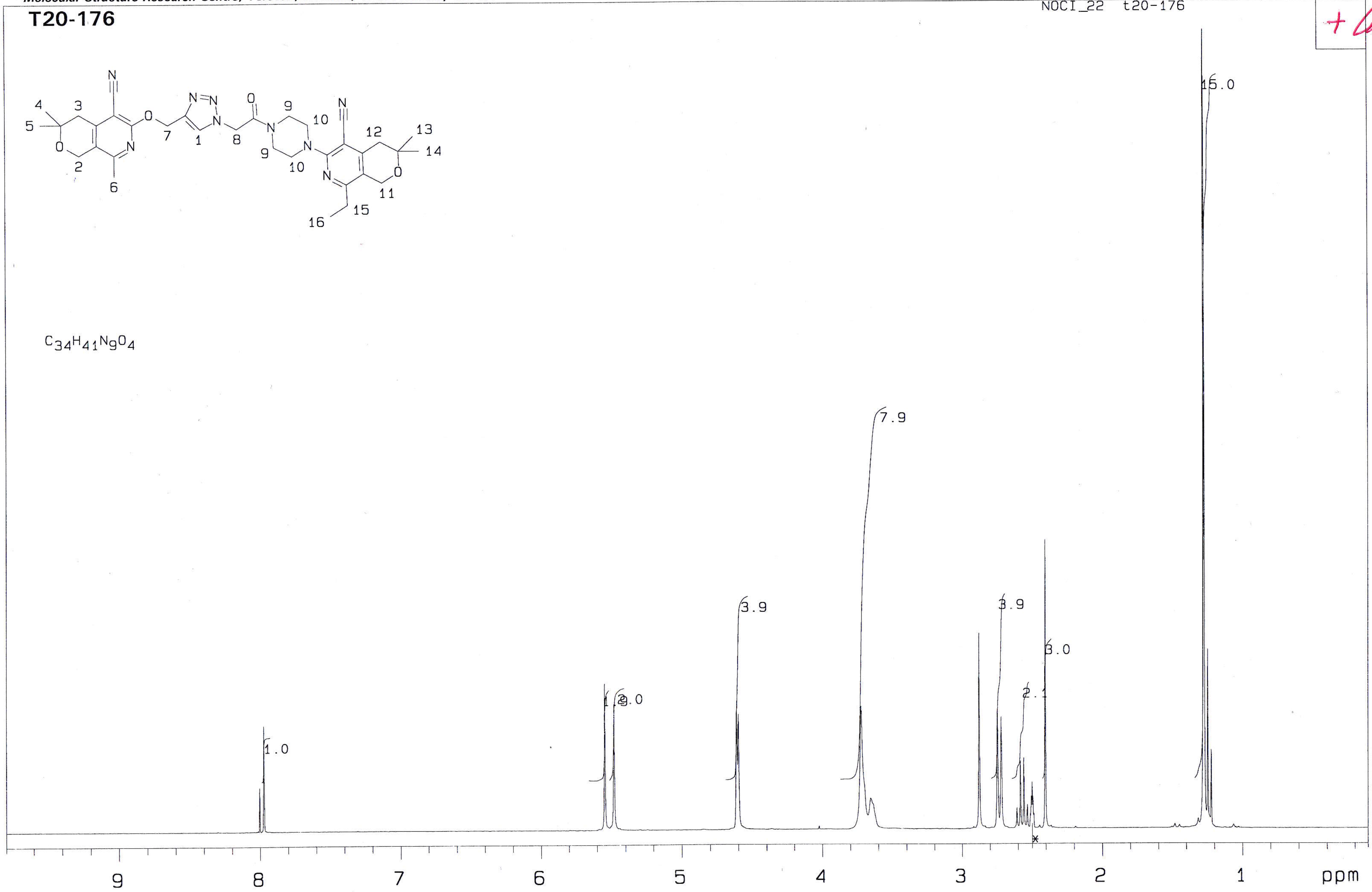

7n

Molecular Structure Research Centre, Yerevan, Armenia, Varian Mercury-300VX

C13 75.465 MHz, nt = 480, np = 19998, temp = 30.0 C, lb = 1.0, solvent = DMSO/CCl4 1/3

Mar 11 2022

NOCI_22 t20-176

T20-176

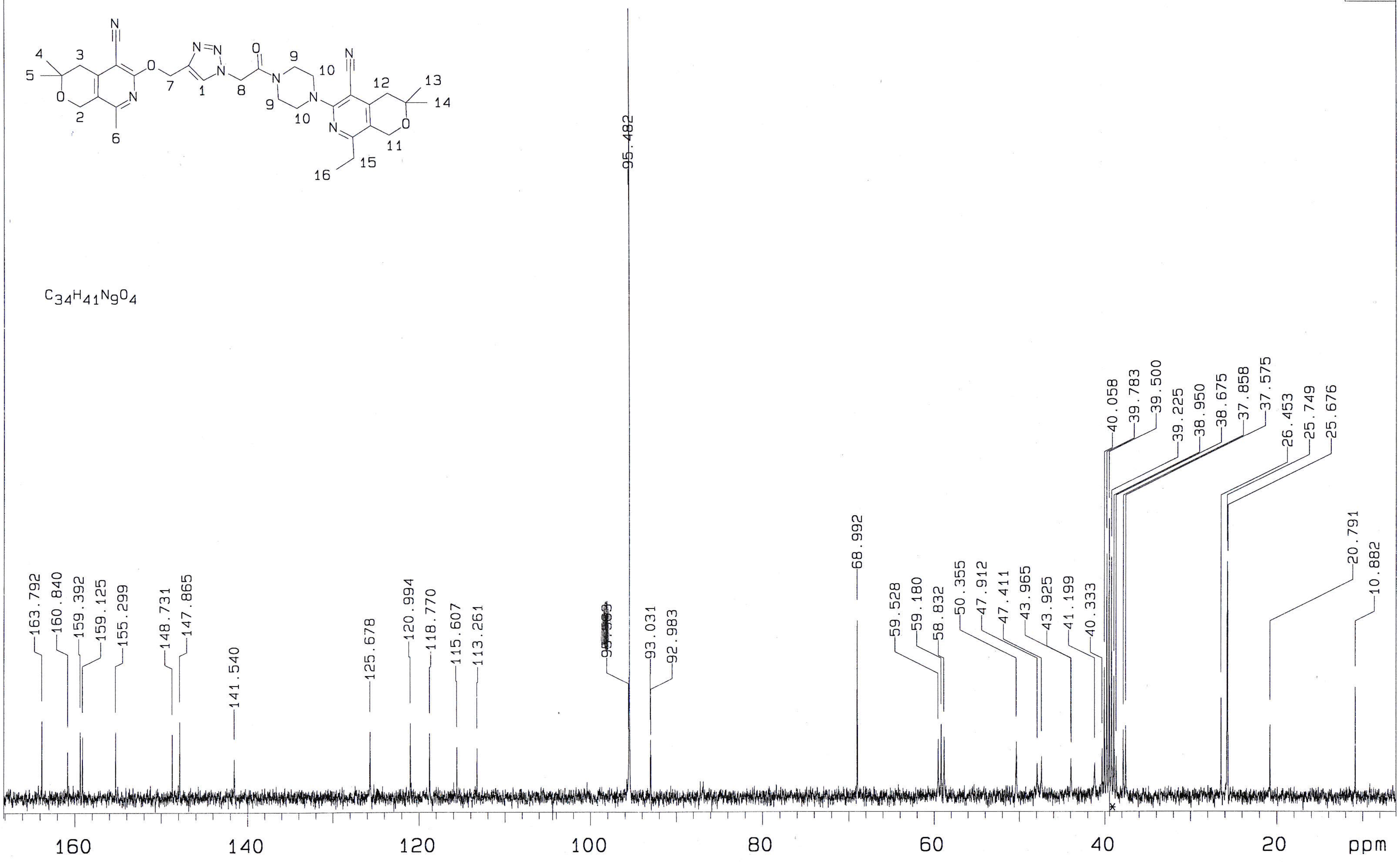

7p

Molecular Structure Research Centre, Yerevan, Armenia, Varian Mercury-300VX H1 300.088 MHz, nt = 16, np = 32000, temp = 30.0 C, lb = -0.2, solvent = DMSO/CCl4 1/3 **Feb 10 2022**

ANUSH_TEMA t20-165

T20-165

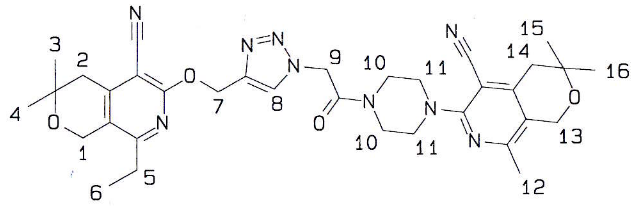

$C_{34}H_{41}N_9O_4$

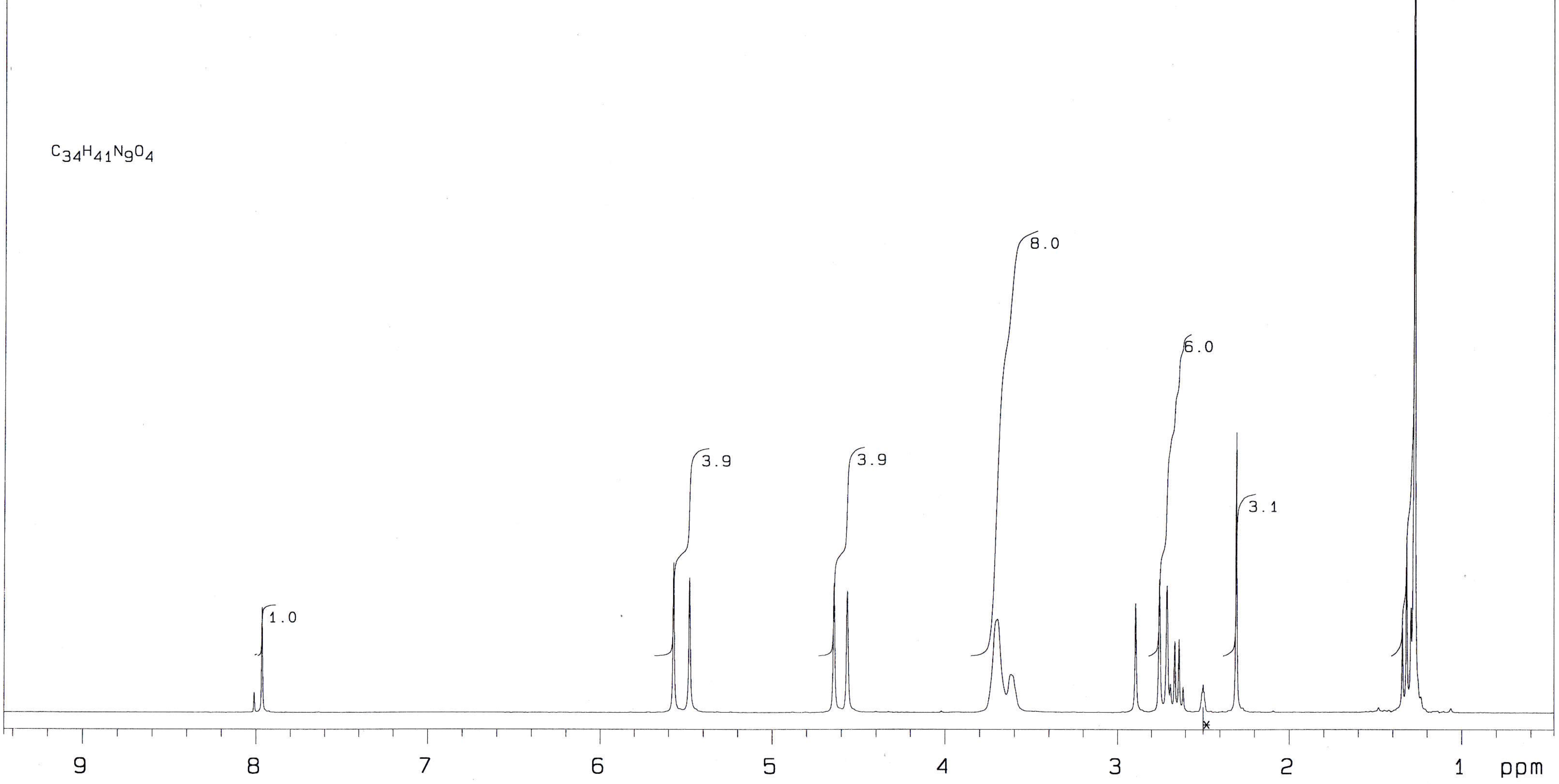

7p

Molecular Structure Research Centre, Yerevan, Armenia, Varian Mercury-300VX

C13 75.465 MHz, nt=512, np=19998, temp=30.0 C, lb=1.0, solvent=DMSO/CCl4 1/3

Feb 10 2022

T20-165

ANUSH_TEMA t20-165

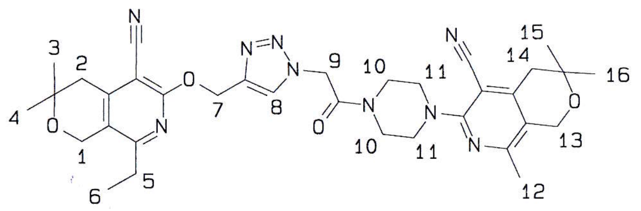

$C_{34}H_{41}N_9O_4$

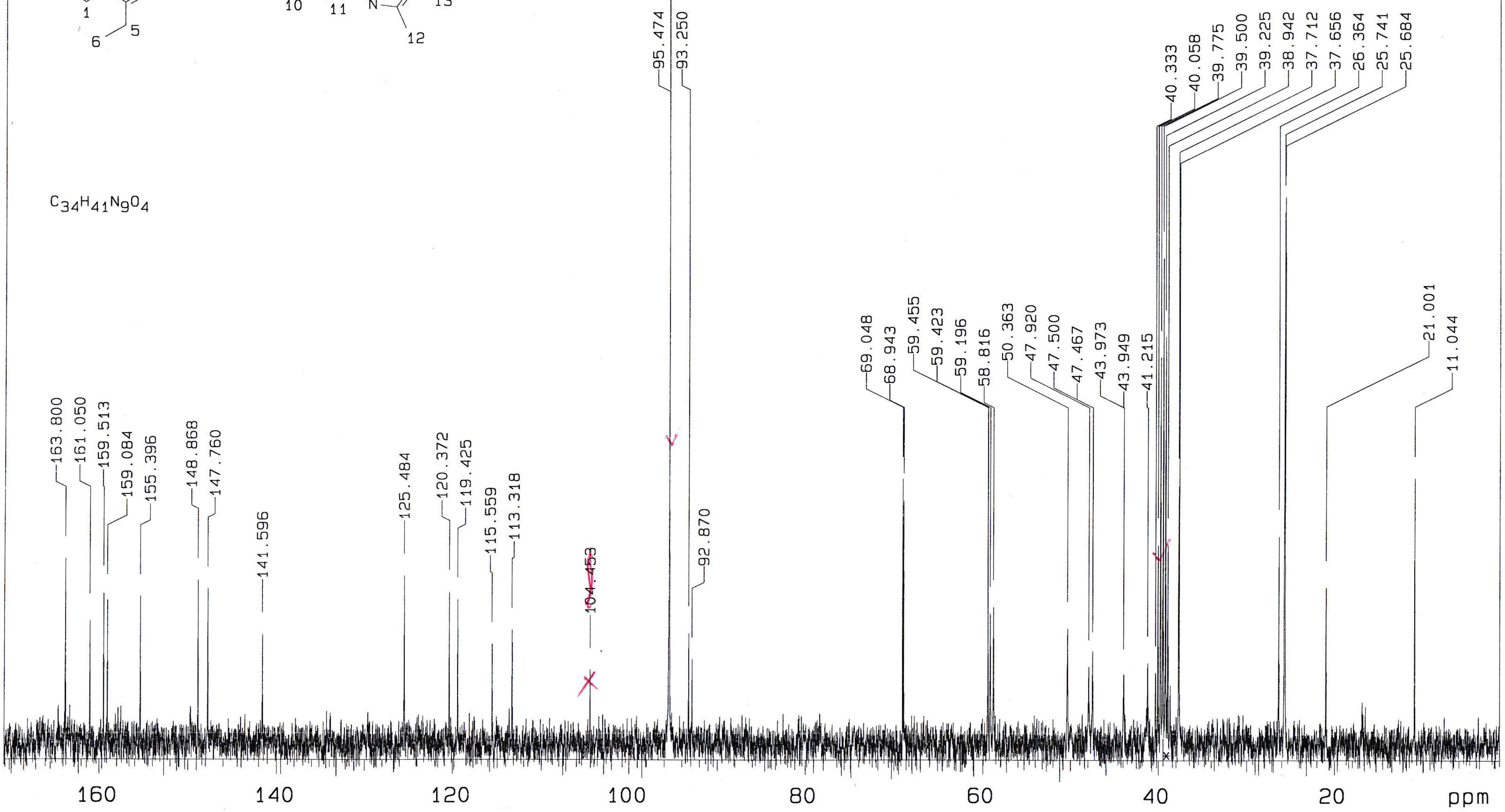

Molecular Structure Research Centre, Yerevan, Armenia, Varian Mercury-300VX

H1 300.088 MHz, nt=16, np=32000, temp=30.0 C, lb=-0.2, solvent=DMSO/CCl4 1/3

Mar 7 2019

7g

NOCI_19 ha-1018

HA-1018

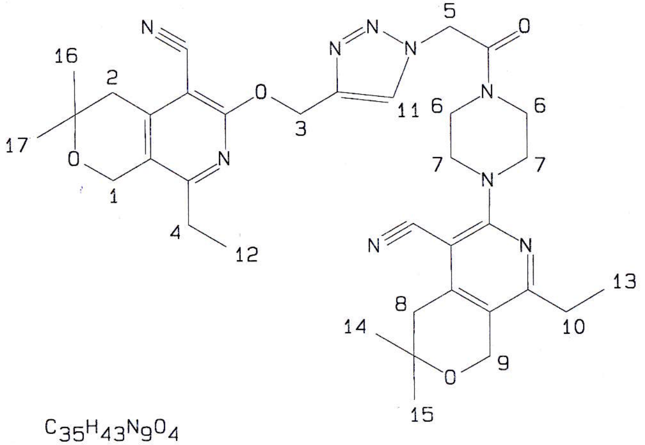

$C_{35}H_{43}N_9O_4$

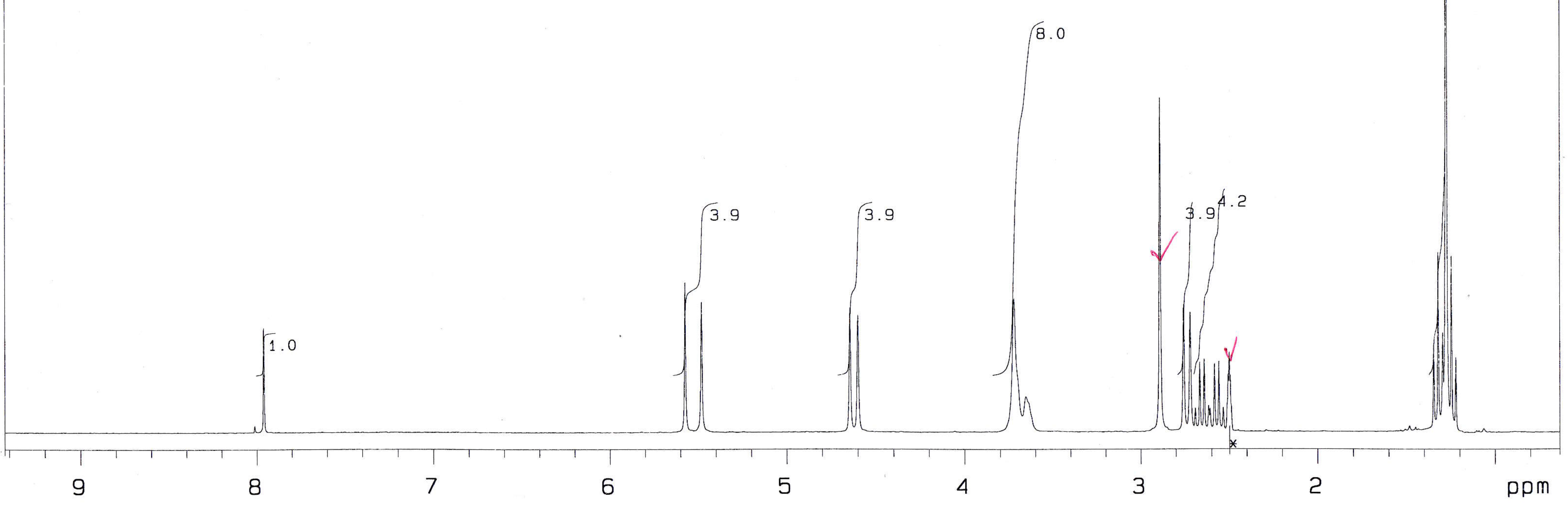

7g

Molecular Structure Research Centre, Yerevan, Armenia, Varian Mercury-300VX

C13 75.465 MHz, nt = 1008, np = 19998, temp = 30.0 C, lb = 1.0, solvent = DMSO/CCl4 1/3

Mar 7 2019

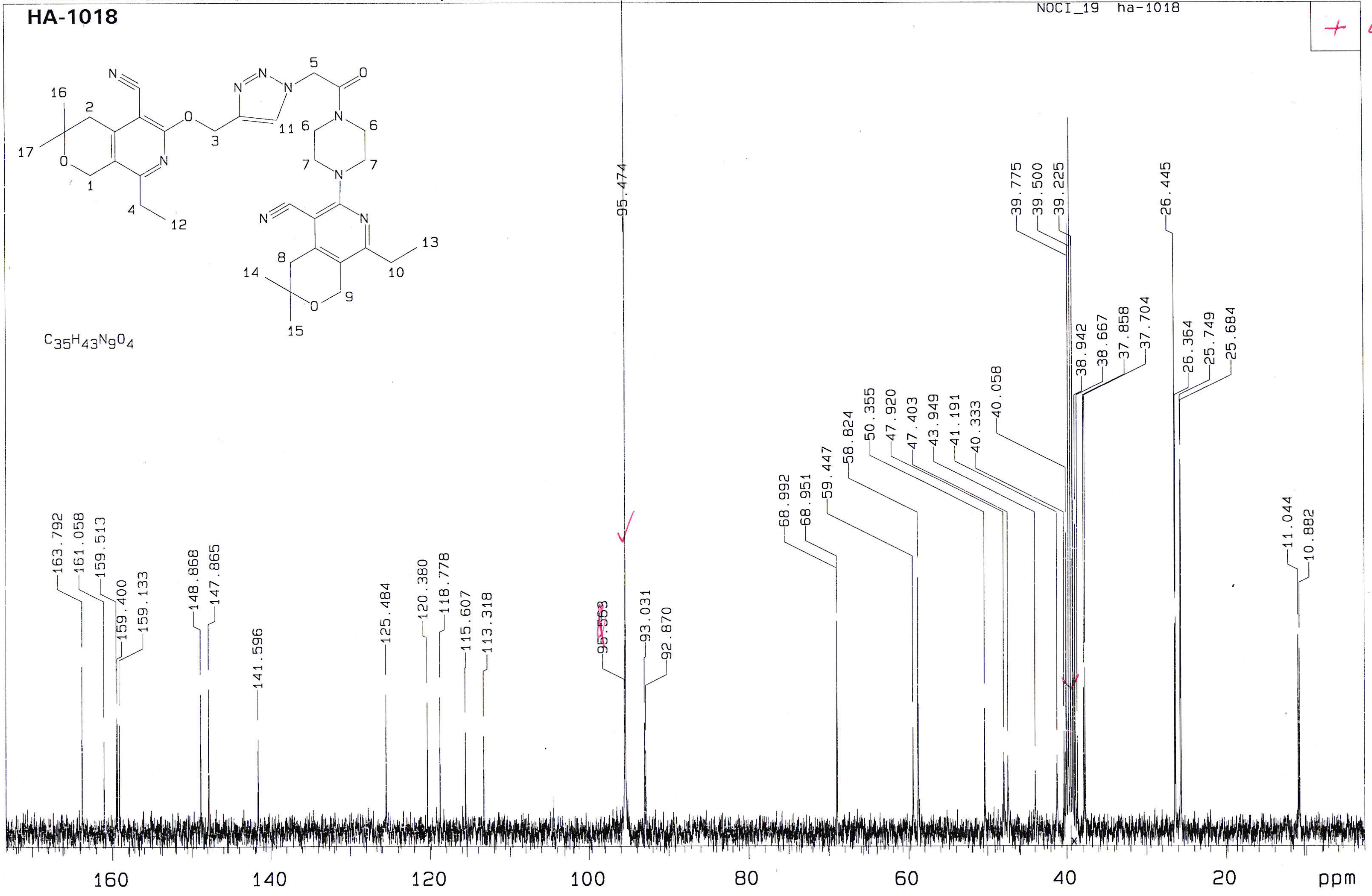

7 g

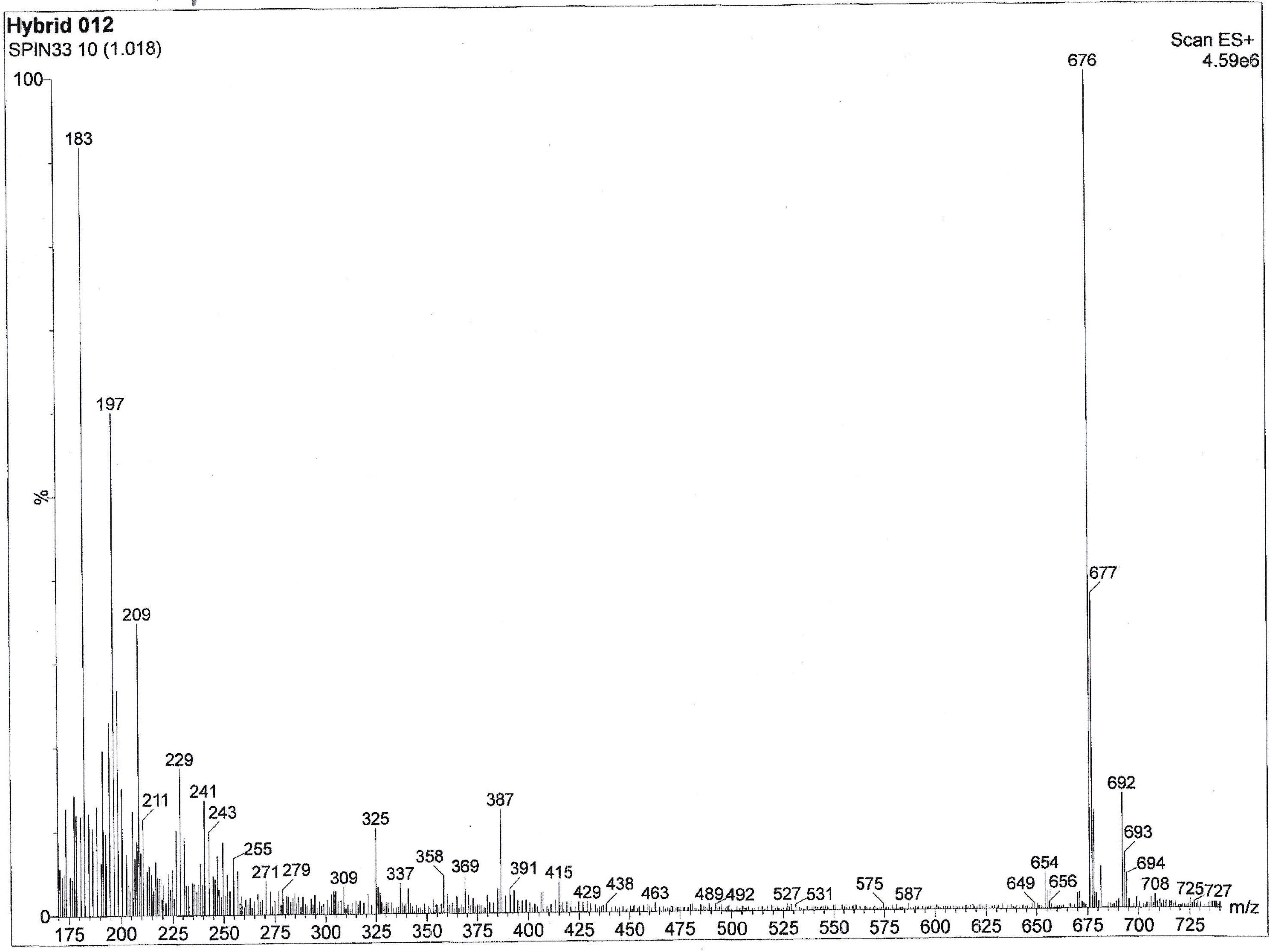
Hybrid 012
SPIN33 10 (1.018)
Scan ES+
4.59e6
100
%
0
676
183
197
209
677
229
692
211
241
243
387
325
693
255
358
654
694
271
279
369
391
415
309
337
649
656
708
725727
438
575
429
463
489492
527 531
587
175 200 225 250 275 300 325 350 375 400 425 450 475 500 525 550 575 600 625 650 675 700 725
m/z

7 g

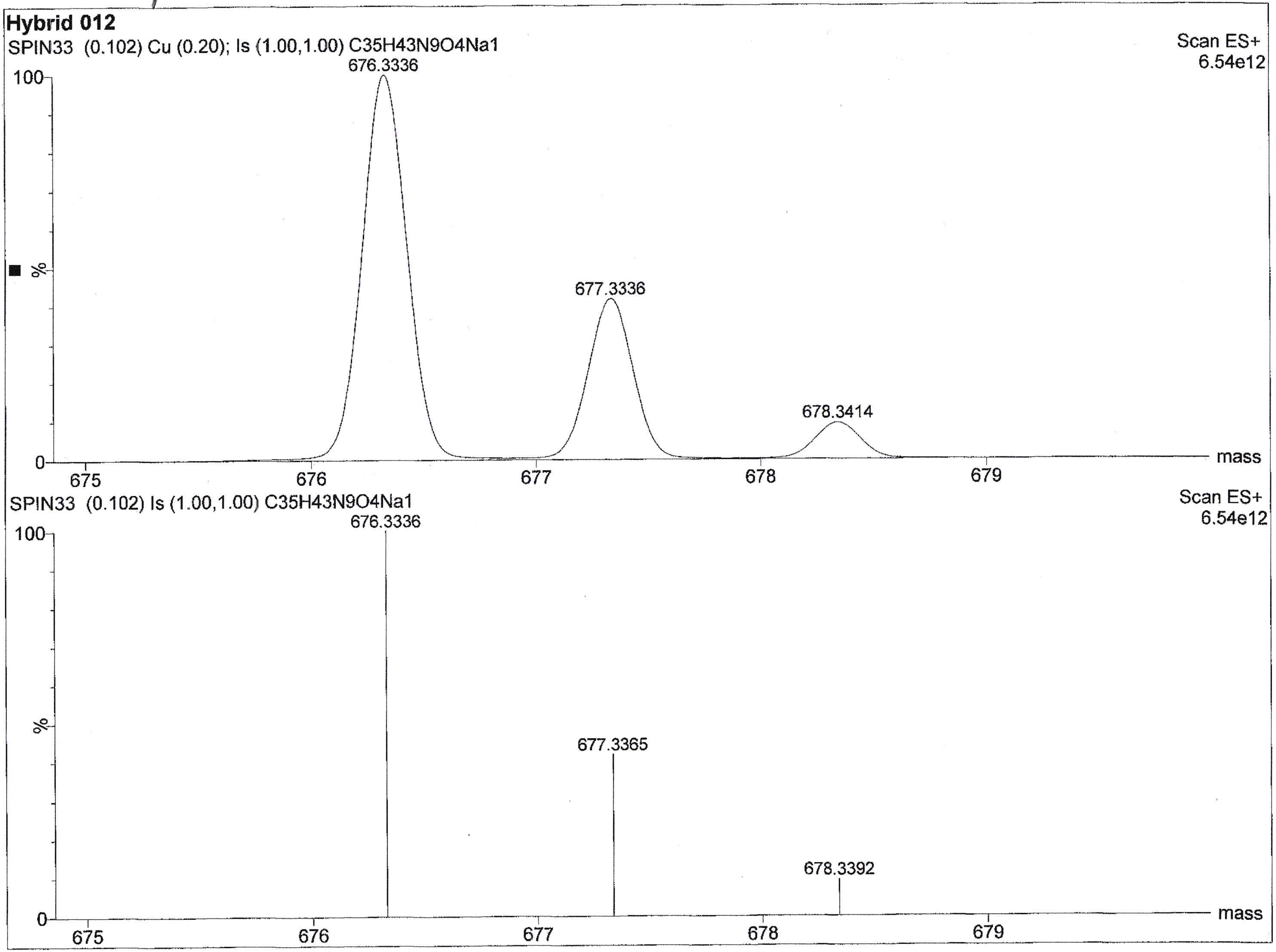
Hybrid 012
SPIN33 (0.102) Cu (0.20); Is (1.00,1.00) C35H43N9O4Na1
Scan ES+
6.54e12
676.3336
677.3336
678.3414
100
%
0
675
676
677
678
679
mass
SPIN33 (0.102) Is (1.00,1.00) C35H43N9O4Na1
Scan ES+
6.54e12
676.3336
677.3365
678.3392
100
%
0
675
676
677
678
679
mass

7z

Molecular Structure Research Centre, Yerevan, Armenia, Varian Mercury-300VX

H1 300.088 MHz, nt = 16, np = 32000, temp = 30.0 C, lb = -0.2, solvent = DMSO/CCl4 1/3

Mar 13 2019

SAMV_19 ha-1020

HA-1020

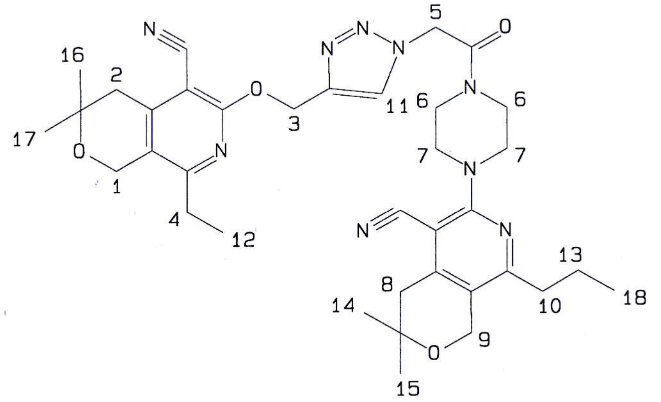

$C_{36}H_{45}N_9O_4$

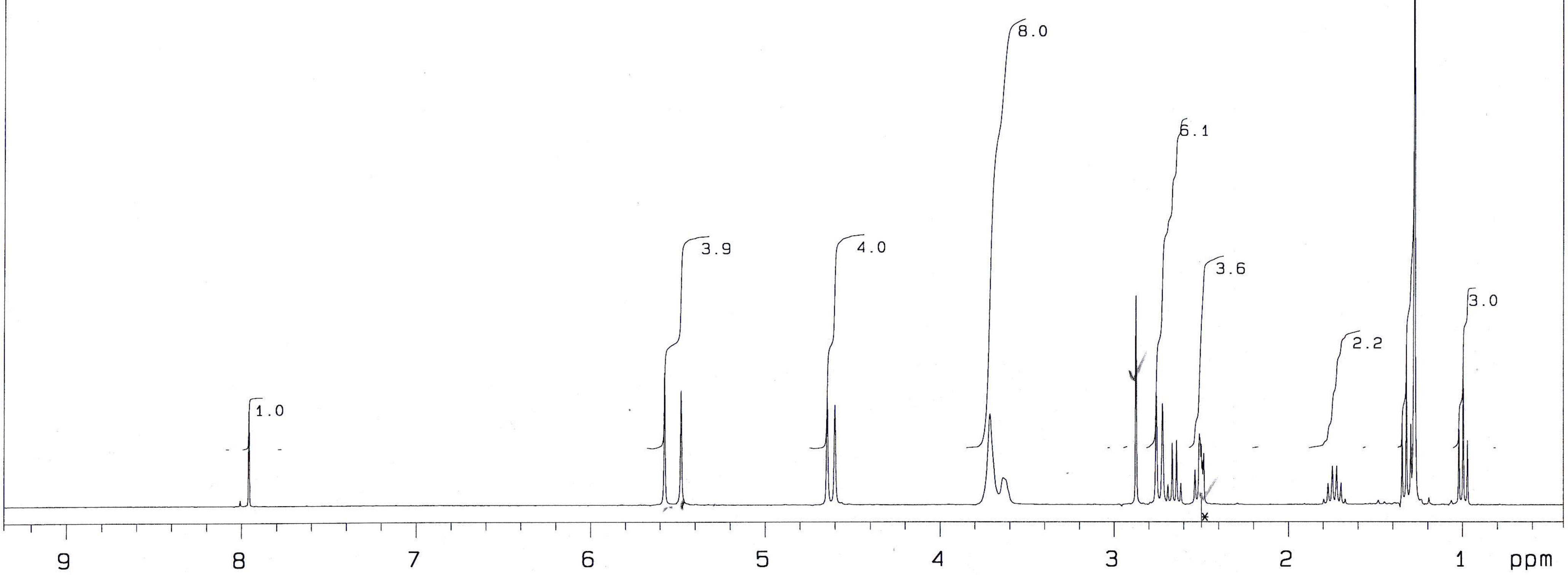

7r

Molecular Structure Research Centre, Yerevan, Armenia, Varian Mercury-300VX C13 75.465 MHz, nt=816, np=19998, temp=30.0 C, lb=1.0, solvent=DMSO/CCl4 1/3 Mar 13 2019

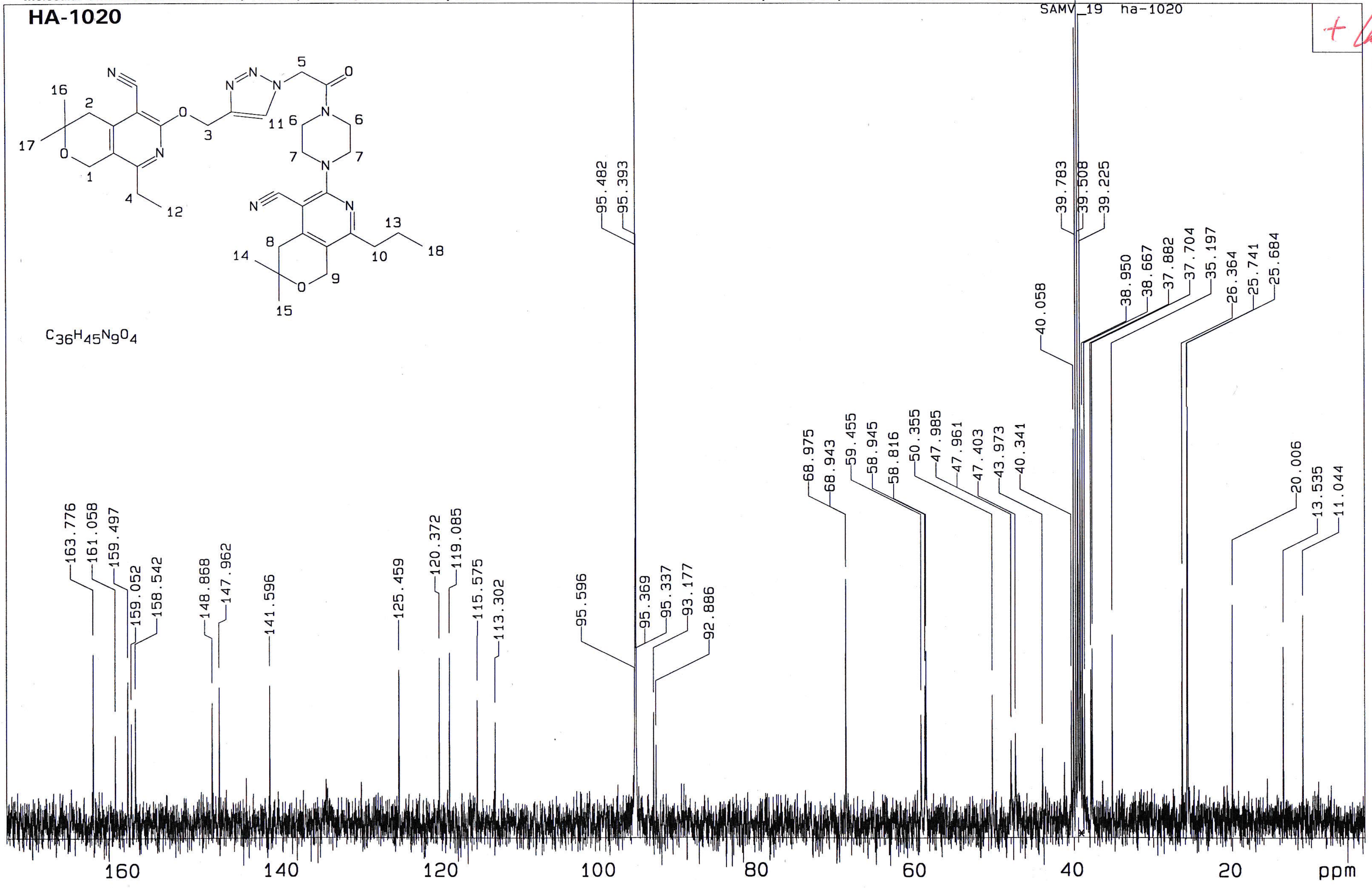

72

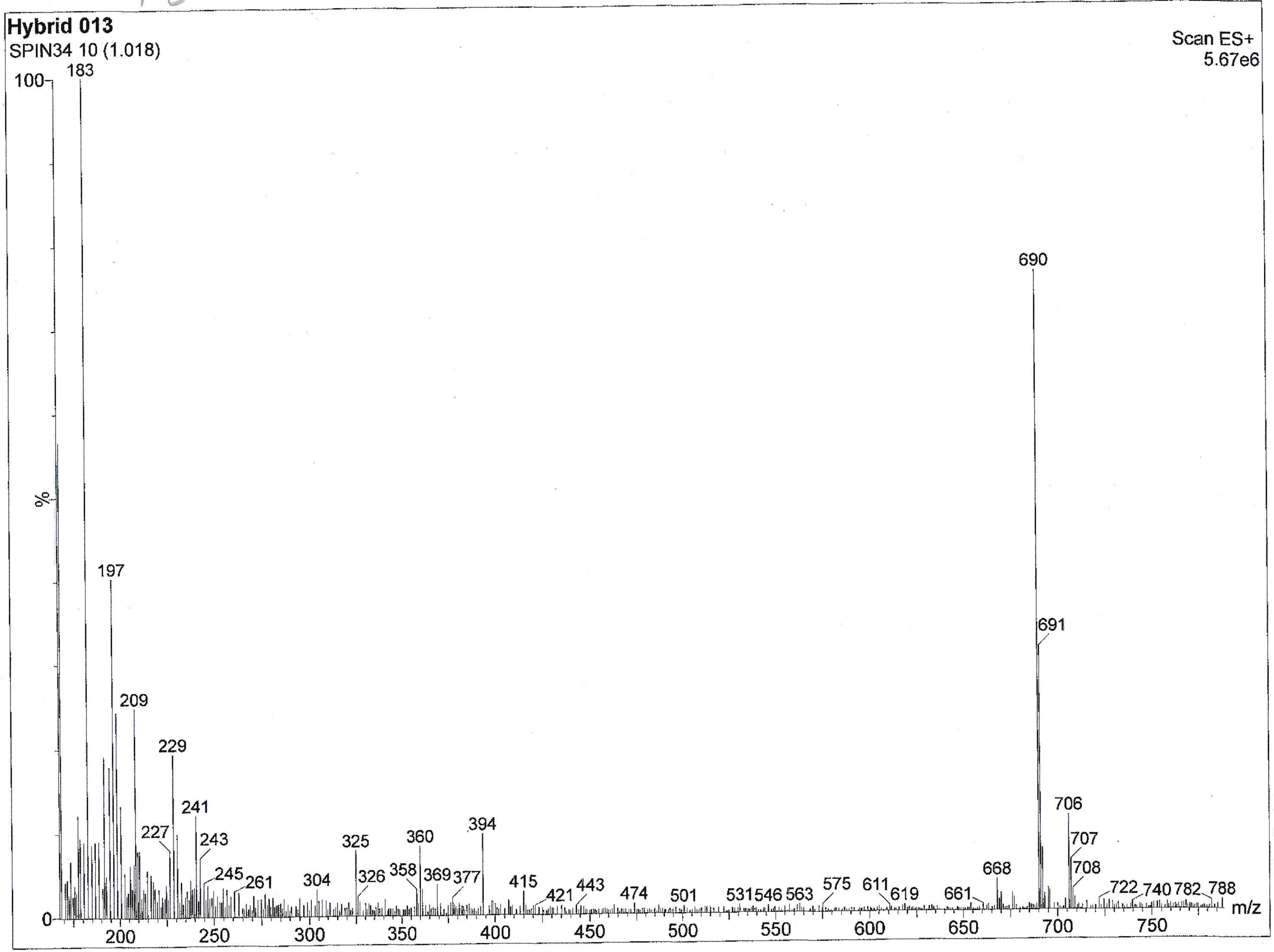
Hybrid 013
SPIN34 10 (1.018)
Scan ES+
5.67e6
100
%
0
183
197
209
227
229
241
243
245
261
304
325
326
358
360
369
377
394
415
421
443
474
501
531
546
563
575
611
619
661
668
690
691
706
707
708
722
740
782
788
200
250
300
350
400
450
500
550
600
650
700
750
m/z

7c

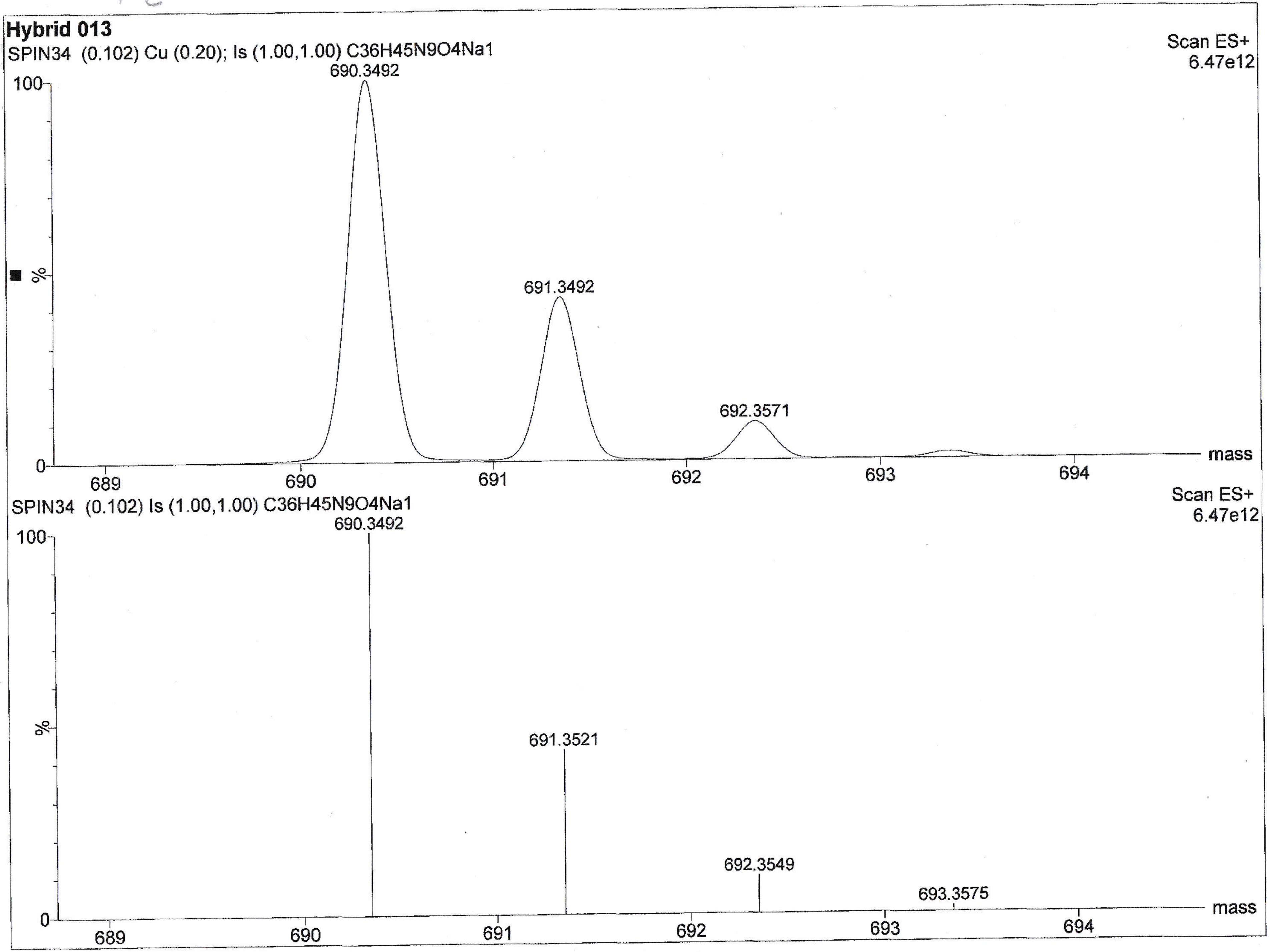
Hybrid 013
SPIN34 (0.102) Cu (0.20); Is (1.00,1.00) C36H45N9O4Na1
Scan ES+
6.47e12
690.3492
691.3492
692.3571
100
%
0
mass
689
690
691
692
693
694
SPIN34 (0.102) Is (1.00,1.00) C36H45N9O4Na1
Scan ES+
6.47e12
690.3492
691.3521
692.3549
693.3575
100
%
0
mass
689
690
691
692
693
694

7s

Molecular Structure Research Centre, Yerevan, Armenia, Varian Mercury-300VX | H1 300.088 MHz, nt = 16, np = 32000, temp = 30.0 C, lb = -0.2, solvent = DMSO/CCl4 1/3 | Apr 30 2019

SAMV_19 ha-1038

HA-1038

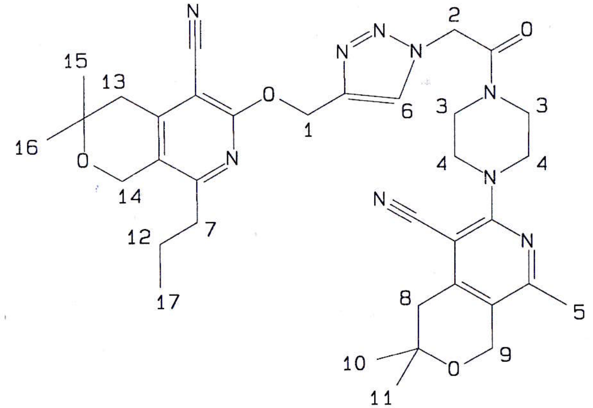

$C_{35}H_{43}N_9O_4$

653.7742

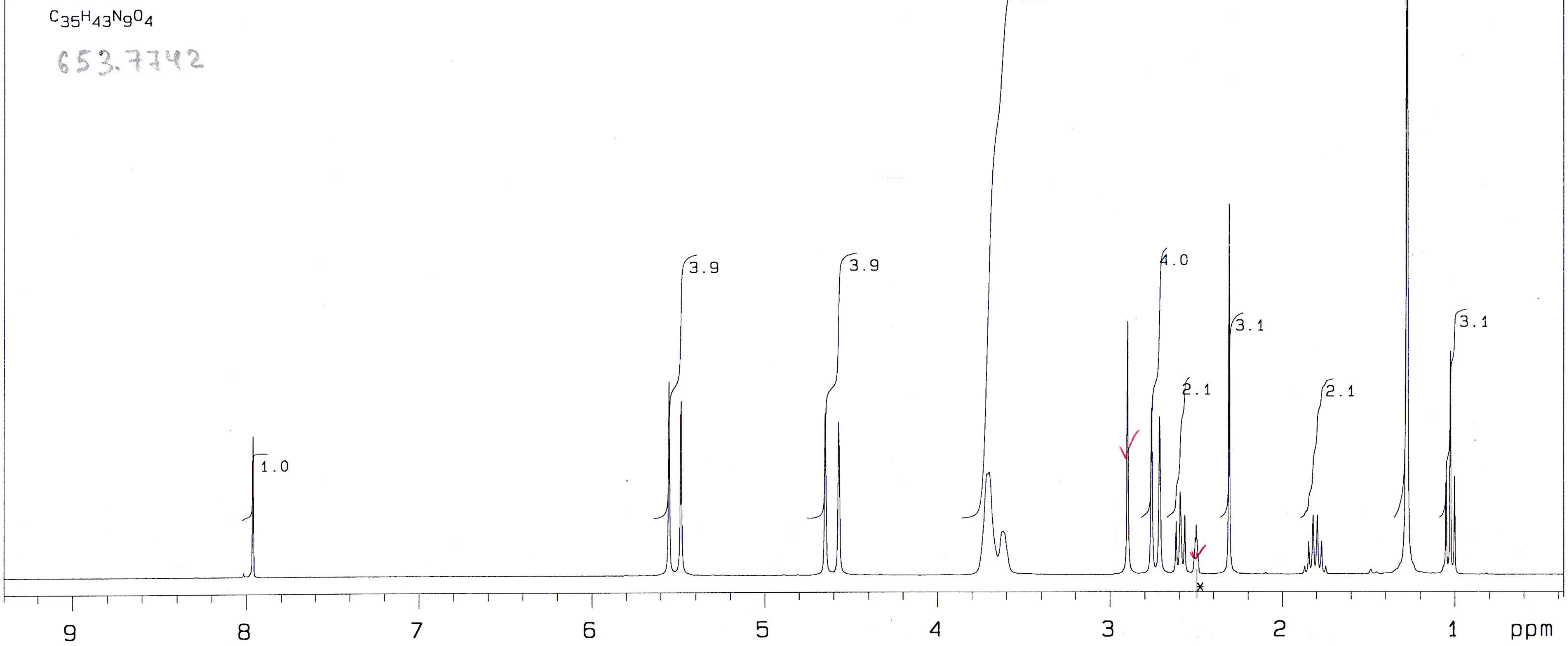

7s

Molecular Structure Research Centre, Yerevan, Armenia, Varian Mercury-300VX — C13 75.465 MHz, nt=896, np=19998, temp=30.0 C, lb=1.0, solvent=DMSO/CCl4 1/3 — Apr 30 2019

SAMV_19 ha-1038

HA-1038

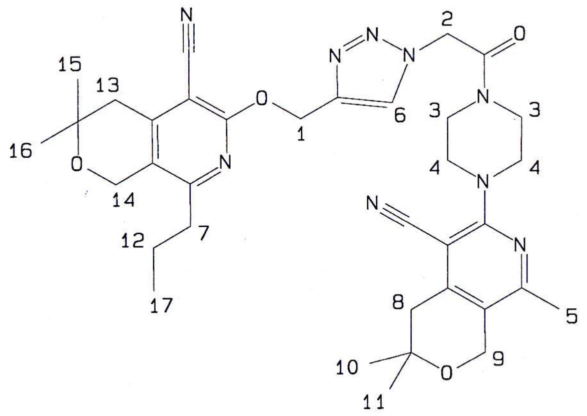

$C_{35}H_{43}N_9O_4$

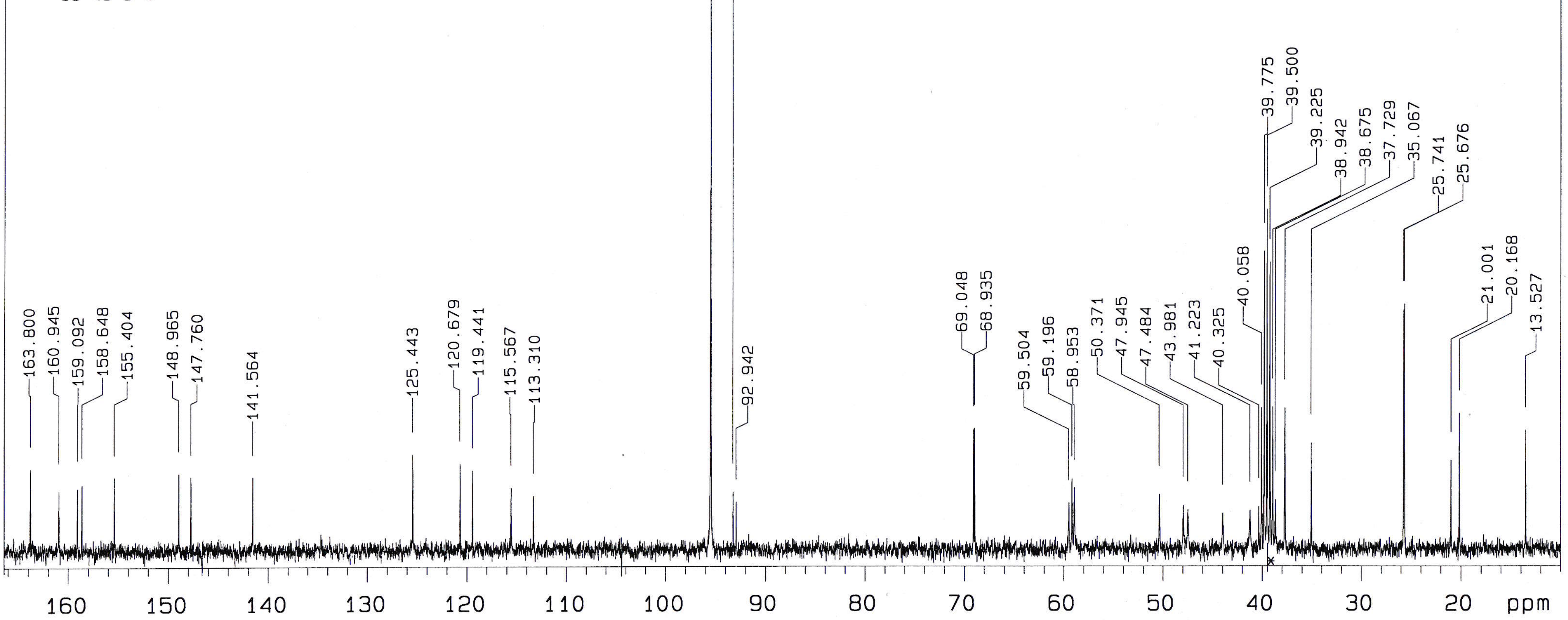

7s

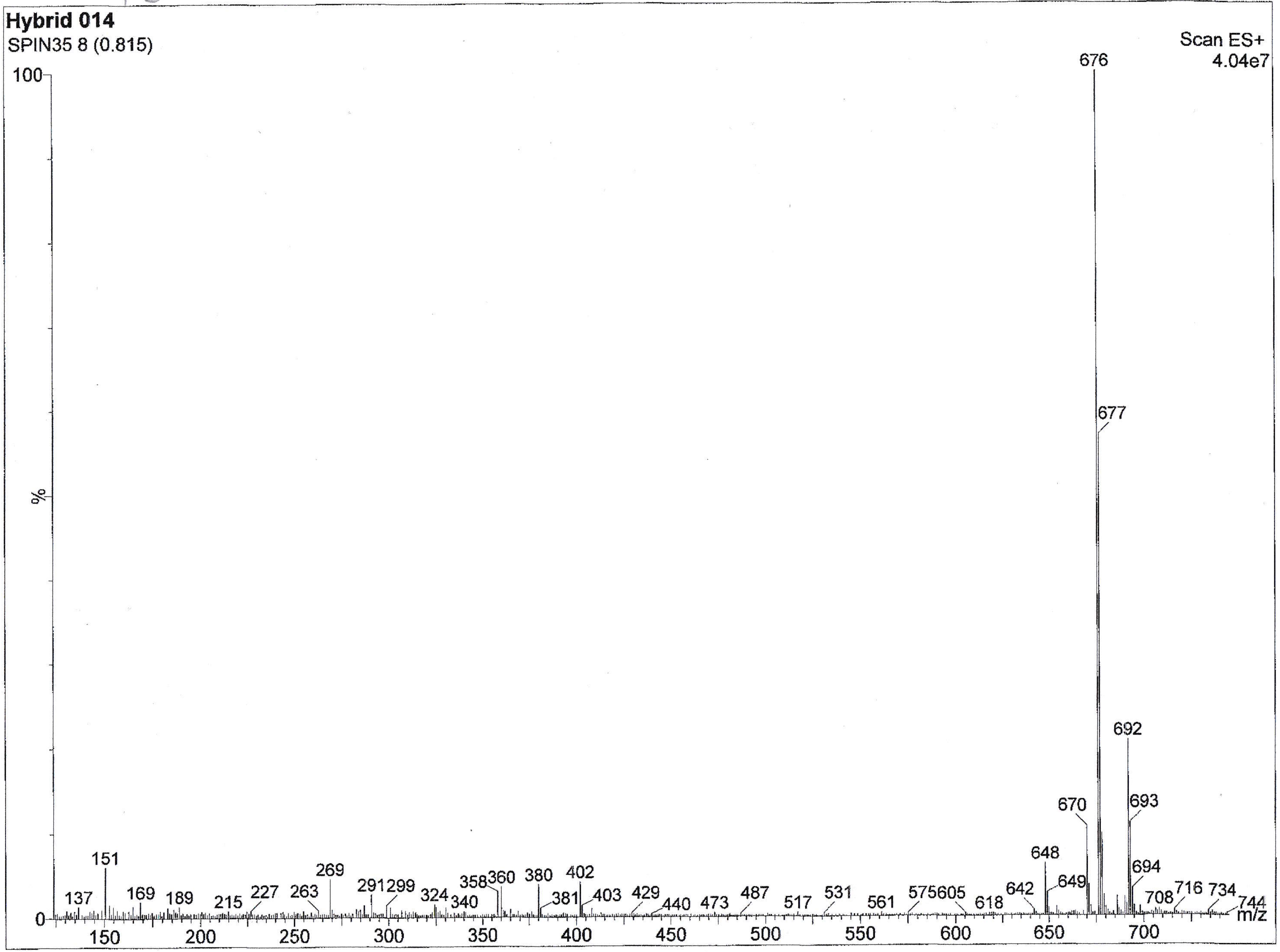

Hybrid 014
SPIN35 8 (0.815)
Scan ES+
4.04e7
100
%
0
676
677
692
693
670
648
649
694
151
137
169
189
215
227
263
269
291
299
324
340
358
360
380
381
402
403
429
440
473
487
517
531
561
575
605
618
642
708
716
734
744
150
200
250
300
350
400
450
500
550
600
650
700
m/z

7s

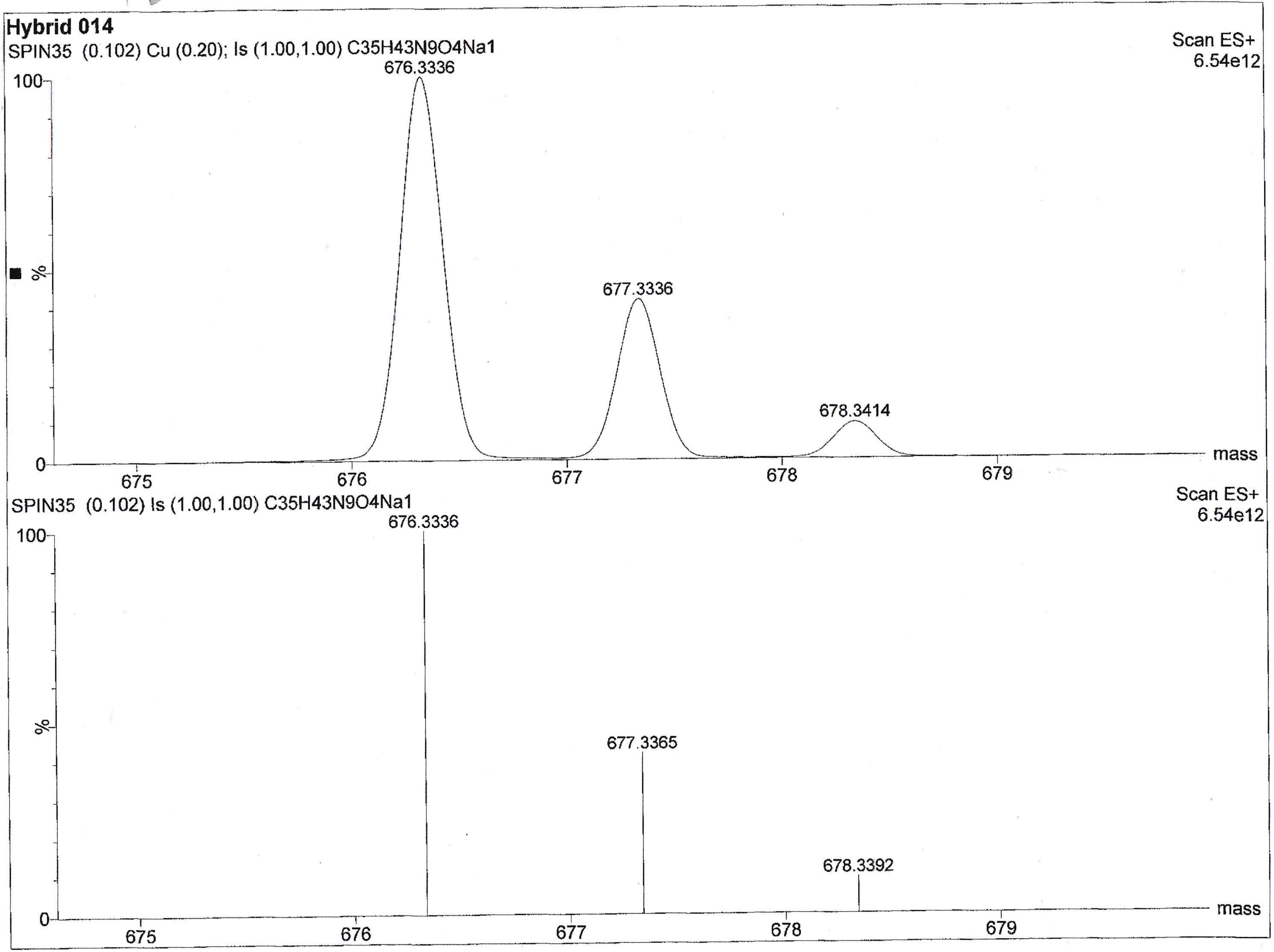
Hybrid 014
SPIN35 (0.102) Cu (0.20); Is (1.00,1.00) C35H43N9O4Na1
Scan ES+
6.54e12
100
%
0
676.3336
677.3336
678.3414
675
676
677
678
679
mass
SPIN35 (0.102) Is (1.00,1.00) C35H43N9O4Na1
Scan ES+
6.54e12
100
%
0
676.3336
677.3365
678.3392
675
676
677
678
679
mass

7t

Molecular Structure Research Centre, Yerevan, Armenia, Varian Mercury-300VX

H1 300.088 MHz, nt = 16, np = 32000, temp = 30.0 C, lb = -0.2, solvent = DMSO/CCl4 1/3

Mar 3 2022

NOCI_22 t20-172

T20-172

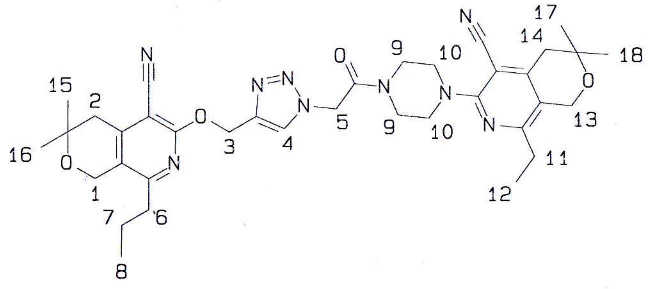

$C_{36}H_{45}N_9O_4$

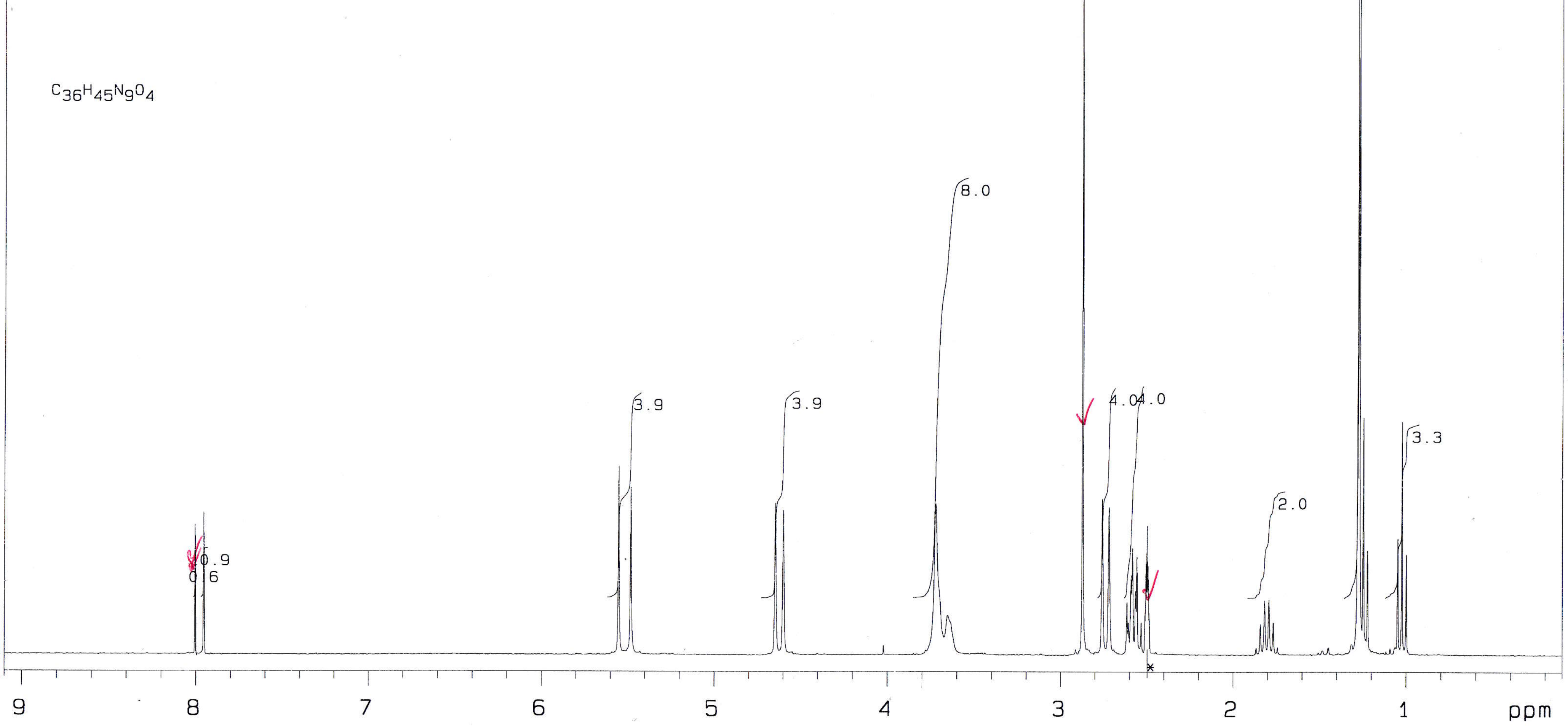

7t

Molecular Structure Research Centre, Yerevan, Armenia, Varian Mercury-300VX

C13 75.465 MHz, nt = 720, np = 19998, temp = 30.0 C, lb = 2.0, solvent = DMSO/CCl4 1/3

Mar 3 2022

NOCI_22 t20-172

T20-172

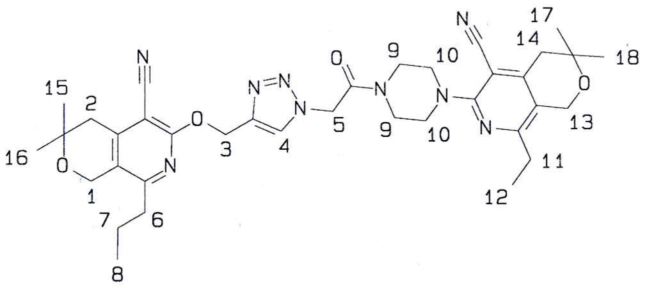

$C_{36}H_{45}N_9O_4$

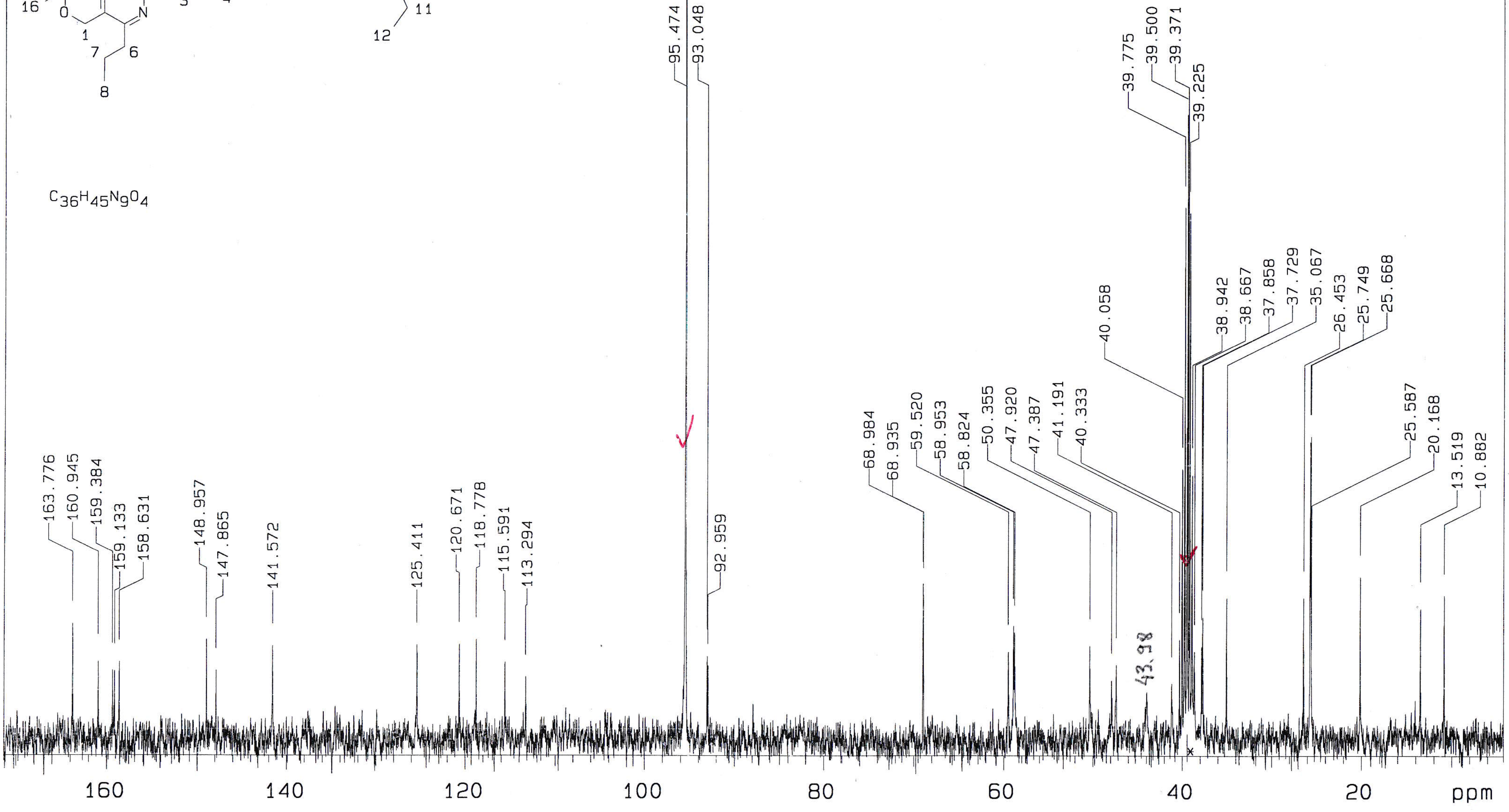

+ [illegible]

7a

Molecular Structure Research Centre, Yerevan, Armenia, Varian Mercury-300VX

H1 300.088 MHz, nt=16, np=32000, temp=30.0 C, lb=-0.2, solvent=DMSO/CCl4 1/3

Apr 5 2019

NOCI_19 ha-1029

HA-1029

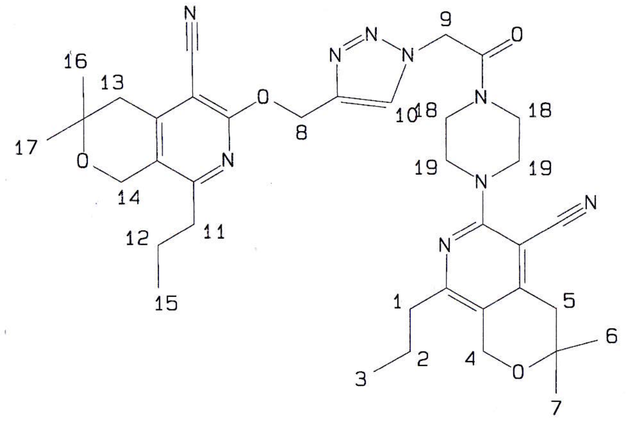

$C_{37}H_{47}N_9O_4$

681.8273

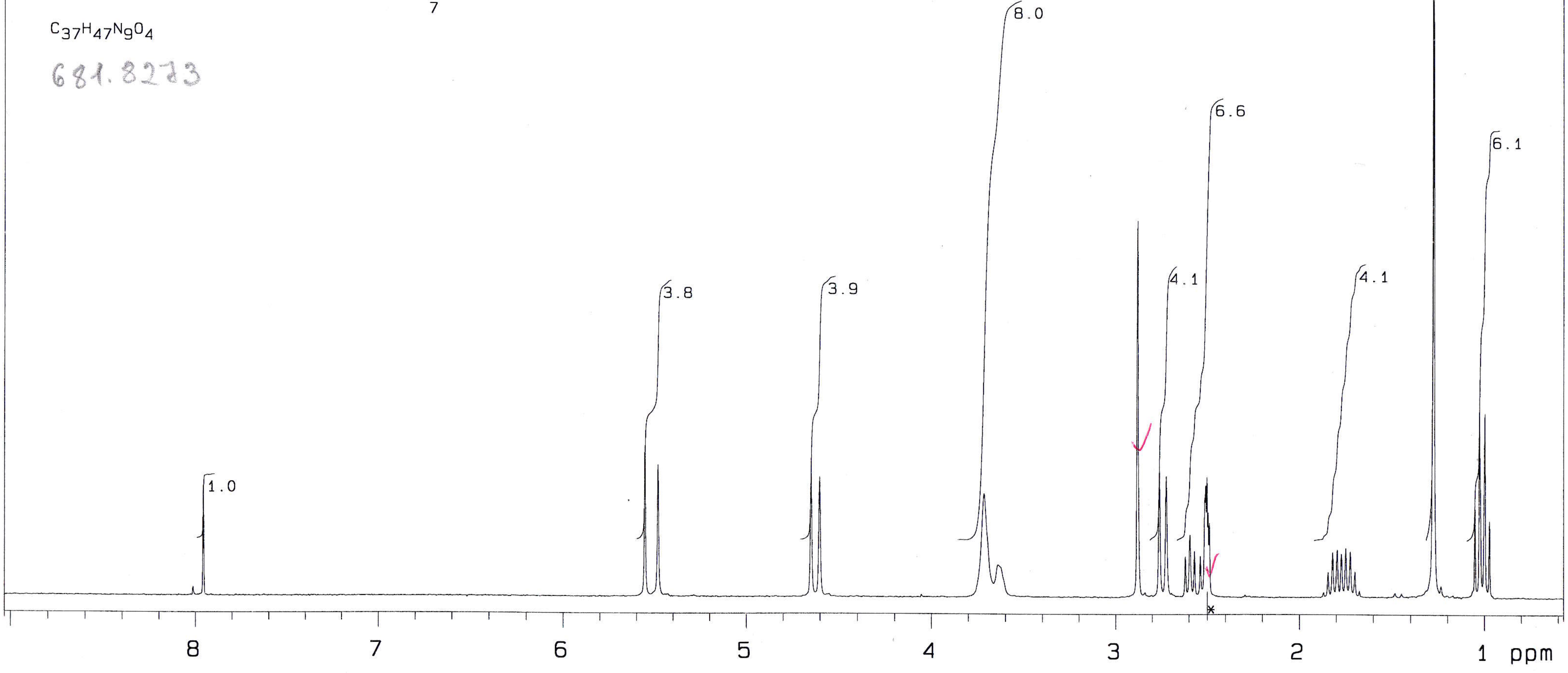

7u

Molecular Structure Research Centre, Yerevan, Armenia, Varian Mercury-300VX

C13 75.465 MHz, nt=672, np=19998, temp=30.0 C, lb=1.0, solvent=DMSO/CCl4 1/3

Apr 5 2019

HA-1029

NOCI_19 ha-1029

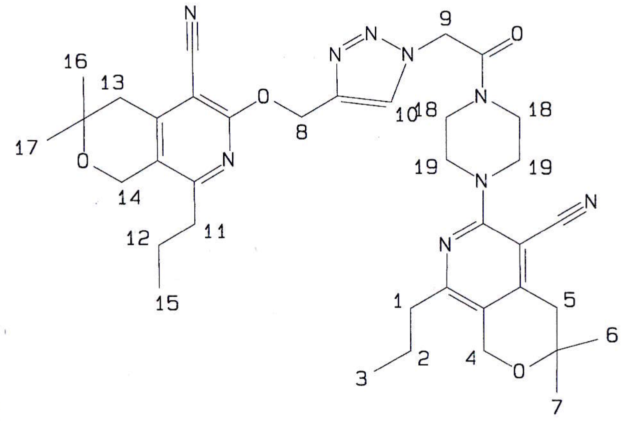

$C_{37}H_{47}N_9O_4$

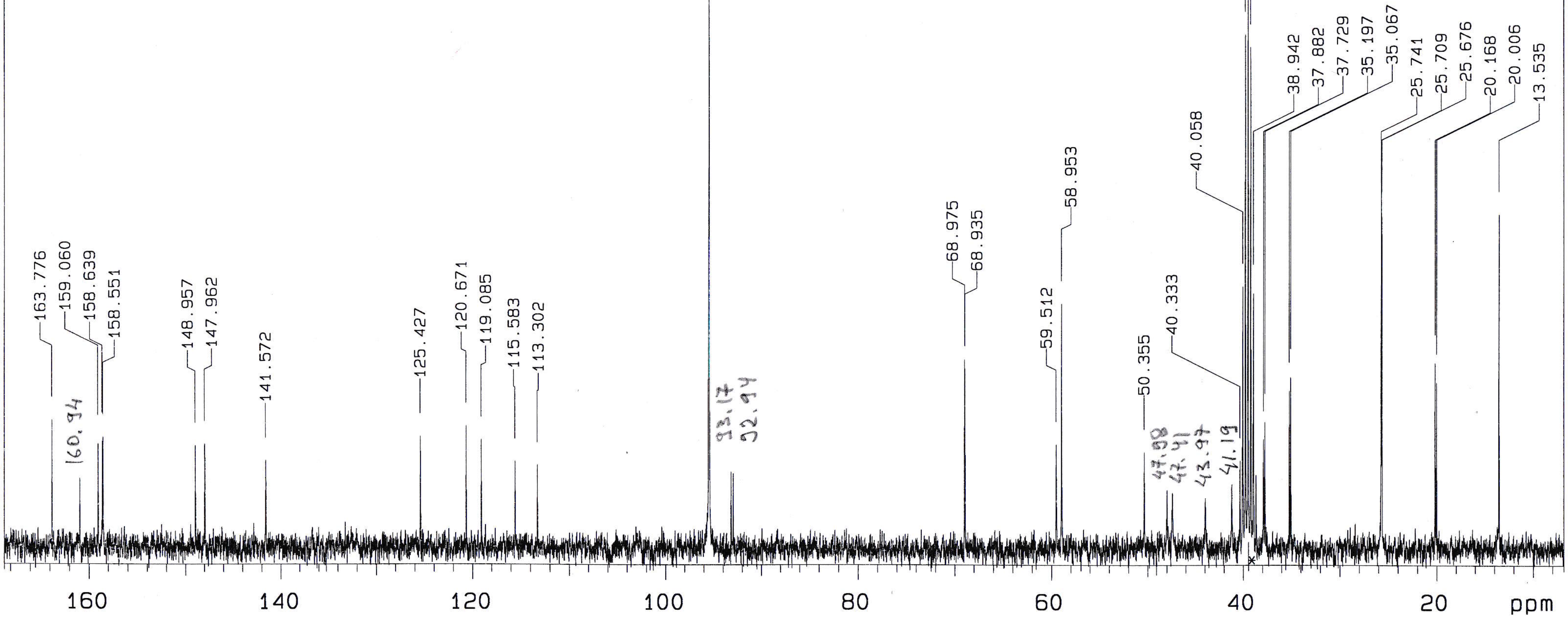

74

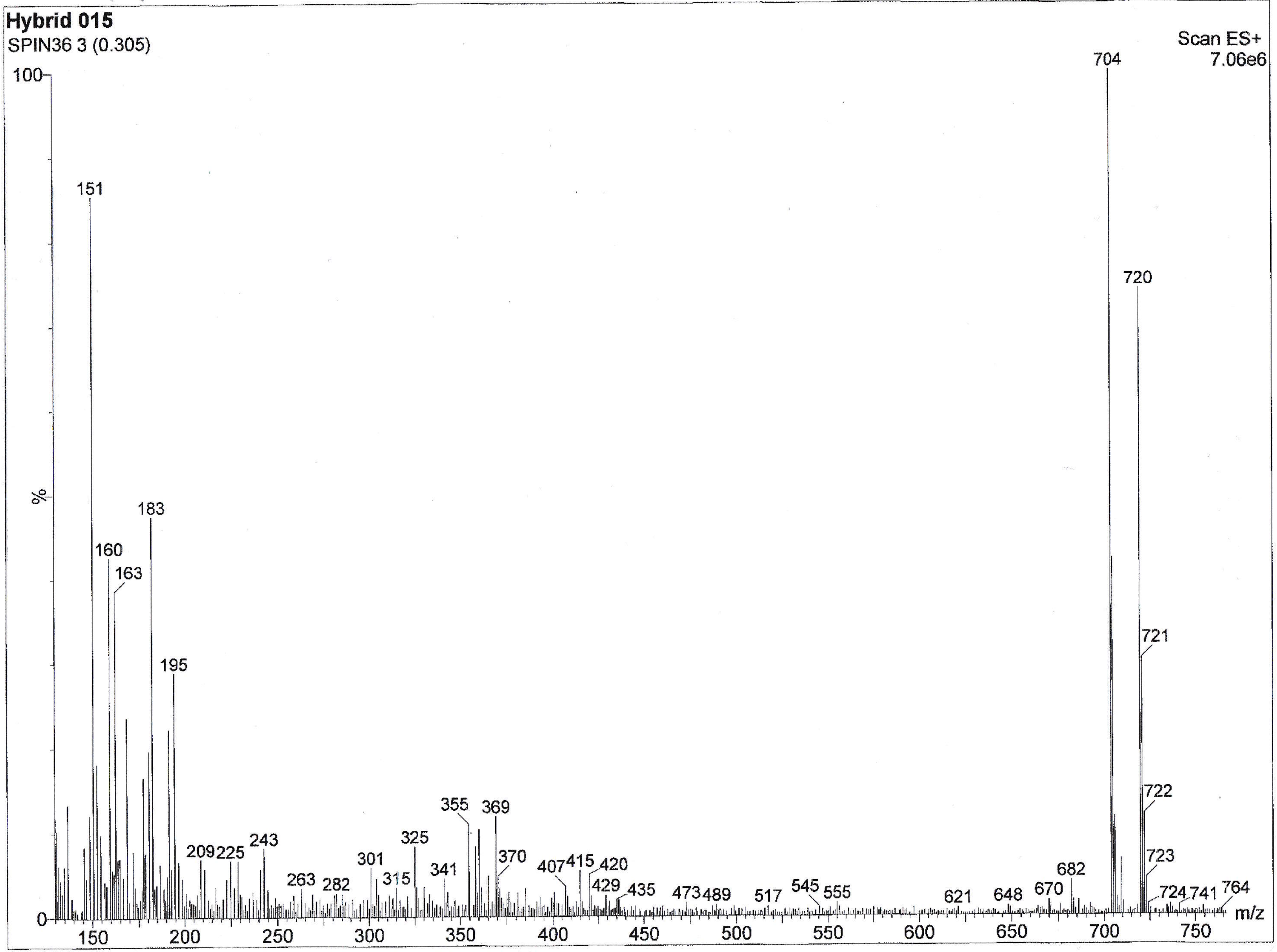
Hybrid 015
SPIN36 3 (0.305)
Scan ES+
7.06e6
100
%
0
704
720
721
722
723
724
741
764
151
160
163
183
195
209
225
243
263
282
301
315
325
341
355
369
370
407
415
420
429
435
473
489
517
545
555
621
648
670
682
150
200
250
300
350
400
450
500
550
600
650
700
750
m/z

74

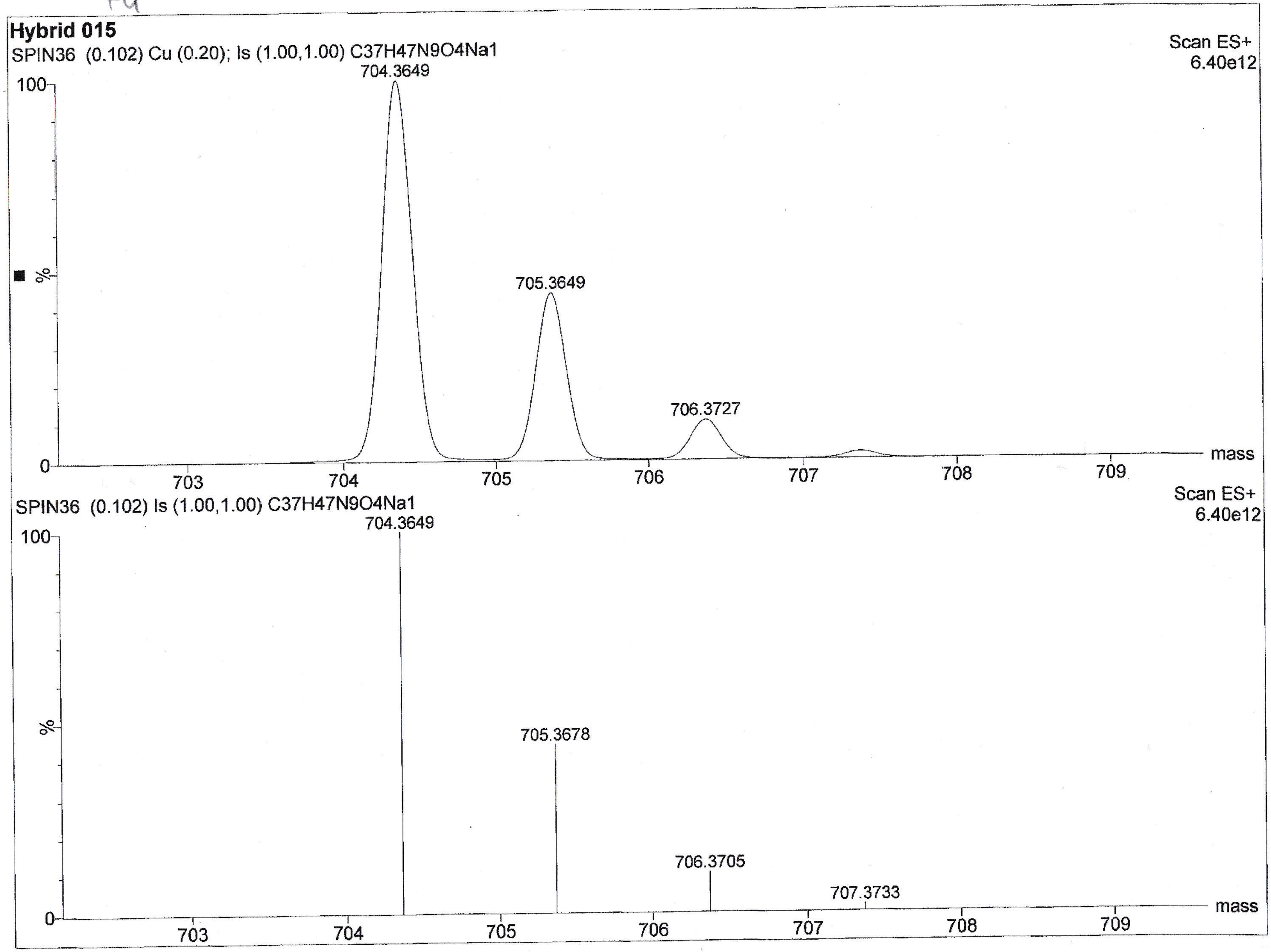

Hybrid 015
SPIN36 (0.102) Cu (0.20); Is (1.00,1.00) C37H47N9O4Na1
Scan ES+
6.40e12
704.3649
705.3649
706.3727
100
%
0
703
704
705
706
707
708
709
mass
SPIN36 (0.102) Is (1.00,1.00) C37H47N9O4Na1
Scan ES+
6.40e12
704.3649
705.3678
706.3705
707.3733
100
%
0
703
704
705
706
707
708
709
mass
